# Effects of Increased Ligand Fields of the Second- and Third-Row Transition Metals on the Preferred Structures of Sandwich Compounds of Stoichiometry C_12_H_12_M

**DOI:** 10.1021/acs.organomet.6c00202

**Published:** 2026-07-31

**Authors:** Yongtao Liu, Haoyu Chen, Jinfeng Luo, Huidong Li, Zhixiang Fan, Qunchao Fan, R. Bruce King, Henry F. Schaefer

**Affiliations:** † School of Science, Key Laboratory of High Performance Scientific Computation, 12598 Xihua University, Chengdu 610039, China; ‡ Center for Computational Quantum Chemistry, 1355 University of Georgia, Athens, Georgia 30602, United States

## Abstract

The lowest-energy sandwich structures for the (C_*n*_H_*n*_)­M­(C_*m*_H_*m*_) (*n* = 5, 6; *m* = 7, 6; *m* + *n* = 12; M = Zr to Pd and Hf to Pt) systems are all singlet or doublet spin state structures, reflecting the increased ligand-field strengths of second- and third-row transition metals relative to first-row transition metals. For Groups 7 and 8, this differs from the first-row transition metals, for which the lowest energy C_12_H_12_M sandwich structures for iron and manganese are high-spin triplet and quartet-spin state dibenzene-metal (η^6^-C_6_H_6_)_2_M (M = Fe, Mn) structures, respectively. For palladium and platinum, the lowest-energy C_12_H_12_M (M = Pd, Pt) structures have two dihapto benzene ligands coordinating to the central metal atom, each through only one of their three C=C double bonds. This contrasts with the nickel C_12_H_12_Ni system, in which a singlet spin state (η^6^-C_6_H_6_)­Ni­(η^2^-C_6_H_6_) structure having one hexahapto and one dihapto benzene ligand is strongly energetically preferred. Similar doublet-spin state (η^6^-C_6_H_6_)­M­(η^2^-C_6_H_6_) (M = Co, Rh, Ir) structures are energetically preferred for the three Group 9 metals. The energetically preferred structures for all of the early transition metals are low-spin (η^6^-C_6_H_6_)_2_M and (η^5^-C_5_H_5_)­M­(η^7^-C_7_H_7_) structures, in which all 12 ring carbon atoms are bonded to the central metal atom.

## Graphical abstract

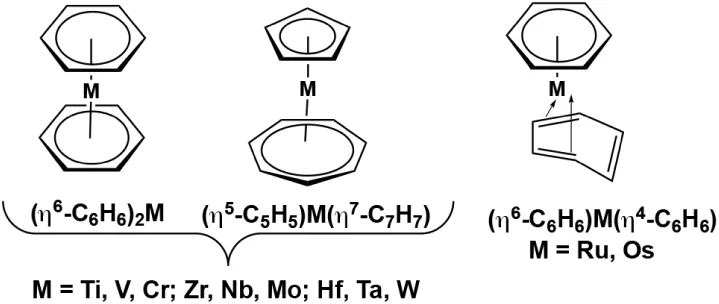

## 1 Introduction

The serendipitous discovery of ferrocene, (η^5^-C_5_H_5_)_2_Fe, by two independent research groups^1,2^ was followed by the study of similar metallocenes of the other first-row transition metals, from titanium to nickel, in which the metal is sandwiched between two planar pentagonal rings. Not long thereafter, the synthesis of sandwich compounds was extended to those of the type (η^6^-C_6_H_6_)_2_M (M = V,^3^ Cr^4–6^ ) containing benzene rather than cyclopentadienyl rings, using the so-called “reducing Friedel-Crafts reaction” starting with an MCl_3_/AlCl_3_/Al/benzene mixture followed by reduction of the initial product in alkaline solution (Figure 1). The subsequent synthesis of the less stable dibenzene-titanium was very different, using the reaction of titanium vapor with benzene vapor.^7,8^

**Figure 1:**
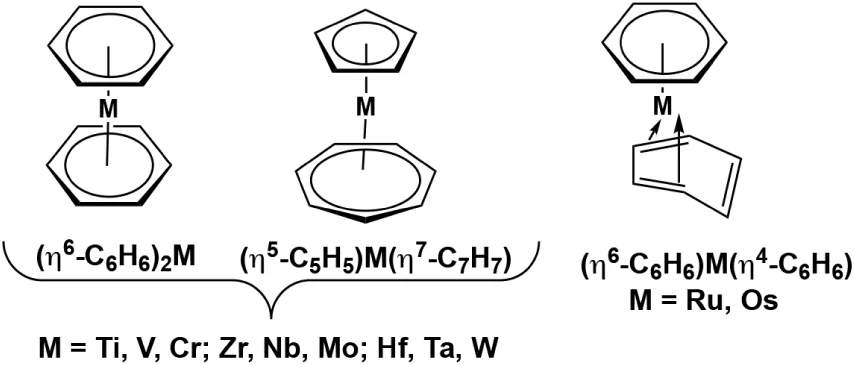
C_12_H_12_M sandwich compounds are known experimentally.

Isomeric with the dibenzene-metal sandwiches (η^6^-C_6_H_6_)_2_M are “mixed” sandwich compounds of the type (η^5^-C_5_H_5_)­M­(η^7^-C_7_H_7_), in which the metal atom is sandwiched between planar five- and seven-membered rings (Figure 1). The first example of such a sandwich compound was the vanadium derivative (η^5^-C_5_H_5_)­V­(η^7^-C_7_H_7_), synthesized by the thermal reaction of (η^5^-C_5_H_5_)­V­(CO)_4_ with cycloheptatriene.^9,10^ The (η^5^-C_5_H_5_)­M­(η^7^-C_7_H_7_) sandwiches of titanium^11,12^ and chromium^13,14^ were subsequently synthesized by various methods using cycloheptatriene as the source of the seven-membered ring.

An important difference between the isomeric (η^6^-C_6_H_6_)_2_M and (η^5^-C_5_H_5_)­M­(η^7^-C_7_H_7_) sandwich compounds is the formal oxidation state of the central metal atom. In the (η^6^-C_6_H_6_)_2_M derivatives, the benzene ligands are neutral, so the formal oxidation state of the metal atom is zero. However, in the (η^5^-C_5_H_5_)­M­(η^7^-C_7_H_7_) derivatives, the planar C_5_H_5_^–^ ligand is the 6π-electron monoanion, and the planar C_7_H_7_^3–^ ligand is the 10π-electron trianion, so the formal oxidation state of the central metal atom is +4. A consequence of the difference in the formal oxidation state of the central metal atoms is that the (η^5^-C_5_H_5_)­M­(η^7^-C_7_H_7_) derivatives are more resistant to air oxidation than their (η^6^-C_6_H_6_)_2_M isomers, an observation already made in 1959 when (η^5^-C_5_H_5_)­V­(η^7^-C_7_H_7_) was first synthesized.^9^

The experimental chemistry of C_12_H_12_M sandwich compounds of the early transition metals has been extended from the first-row metals to the second- and third-row metals, with metal vapor synthesis being an important synthetic method. The Group 6 metal benzene derivatives (η^6^-C_6_H_6_)_2_M (M = Mo,^15^ W^16^) were initially synthesized by the Fischer group by the MCl_*n*_/AlCl_3_/Al reaction. Subsequently metal vapor synthesis was shown to provide the tungsten derivative in much higher yields.^17^ The (η^6^-C_6_H_6_)_2_M (M = Nb, Ta) sandwiches are known for the Group 5 metals but do not appear to be stable species for the second- and third-row group 4 metals, zirconium and hafnium. However, the unstable (η^6^-C_6_H_6_)_2_M (M = Zr, Hf) generated in the metal vapor reactor can be trapped as their trimethylphosphine complexes (η^6^-C_6_H_6_)_2_MPMe_3_ with 18-electron metal configurations. The mixed sandwich compounds (η^5^-C_5_H_5_)­M­(η^7^-C_7_H_7_) (M = Zr,^18^ Nb,^18,19^ Mo;^18,20,21^ Hf,^22^ Ta,^19^ W^23,24^ ), with the favored 18-electron metal configuration, are known for all of the Group 4, 5, and 6 metals.

The neutral sandwich C_12_H_12_M compounds of transition metals of Groups 7 through 10, with all 12 carbon atoms of the two rings bonded to the central metal atom, have metal atom configurations in excess of the favored 18-electron configuration and thus are expected to be significantly less stable. However, dibenzenemetal cations [(η^6^-C_6_H_6_)_2_M]^+^ (M = Mn, Tc, Re) of the Group 7 metals and dibenzenemetal dications [(η^6^-C_6_H_6_)_2_M]^2+^ (M = Fe, Ru, Os) of the Group 8 metals, with fully bonded hexahapto benzene ligands, have the favored 18-electron metal configurations and have been synthesized as stable salts with suitable counteranions. Reduction of such species provides a potential source of neutral (C_6_H_6_)_2_M derivatives. However, no such species with unsubstituted benzene ligands have been proven to be stable at room temperature. Such derivatives can be stabilized by using hexamethylbenzene as a ligand rather than unsubstituted benzene. The difference in stability is demonstrated by experimental work on the ruthenium system. Thus, dibenzene-ruthenium can be synthesized from ruthenium vapor and benzene as an orange solid, characterized by proton NMR at low temperatures, but decomposing at room temperature.^25^ However, neutral bis­(hexamethylbenzene)­ruthenium, synthesized by reduction of the corresponding dication [(η^6^-Me_6_C_6_)_2_Ru]^2+^, is a diamagnetic solid stable at room temperature and has been shown by X-ray crystallography to have one hexahapto Me_6_C_6_ ring and one bent rather than planar tetrahapto Me_6_C_6_ ring with one uncomplexed C=C double bond (Figure 1), thereby giving its ruthenium atom the favored 18-electron configuration.^26^ Analogous osmium derivatives are known.^27,28^

The diamagnetic hexahapto-tetrahapto (η^6^-Me_6_C_6_)­Ru­(η^4^-Me_6_C_6_) structure for the derivative of the second-row transition metal ruthenium contrasts with the paramagnetic, presumed bis­(hexahapto) structure (η^6^-Me_6_C_6_)_2_Fe for its first-row transition metal congener iron. Thus, the iron atom in (η^6^-Me_6_C_6_)_2_Fe has a 20-electron configuration similar to that of the nickel atom in nickelocene (η^5^-C_5_H_5_)_2_Ni.^29,30^ This difference between the diamagnetic (η^6^-Me_6_C_6_)­Ru­(η^4^-Me_6_C_6_) and the paramagnetic (η^6^-Me_6_C_6_)_2_Fe is undoubtedly a consequence of the larger ligand-field strength in complexes of the second-row transition metal ruthenium relative to the corresponding first-row transition metal iron.

This difference in the experimentally preferred structures and spin states for the (Me_6_C_6_)_2_M complexes of iron and ruthenium suggests the possibility of other differences between the preferred structure types of C_12_H_12_M derivatives of the second and third rows of the transition metals relative to those of the corresponding first-row transition metal derivatives. We now report new density functional studies on C_12_H_12_M derivatives of the second- and third-row transition metals for comparison with our previous results on the analogous first-row transition metal derivatives^31^ using similar density functional theory methods.

## 2 Theoretical Methods

The hybrid *meta*-GGA DFT method M06-L^32,33^ and the B3PW91 method^34,35^ as implemented in the Gaussian 16 program^36^ were used for the computations. These methods have been reported to give better overall performance for organometallic compounds than the first-generation functionals.^37^ For carbon, hydrogen, and the first-row transition metals, the def2-TZVP basis sets^38,39^ were used in conjunction with the M06-L and B3PW91 methods for the DFT optimizations. For the 4d and 5d transition metals, the energy-consistent quasi-relativistic pseudopotentials of the Stuttgart/Cologne Group, with 28 or 60 core electrons for the 4d or 5d transition metals, respectively, and the corresponding contracted basis sets were used.^40,41^

The geometries of all structures were fully optimized using the above DFT method with the (120, 974) integration grid. Small imaginary frequencies for the optimized structures were assumed to originate from numerical integration errors. The optimized (C_*n*_H_*n*_)­M­(C_*m*_H_*m*_) (*n* = 6, 5, 4; *m* = 6, 7, 8; *n* + *m* = 12) structures in this paper are designated as **Mz−nmX**, where **M** is the chemical symbol of the transition metal atom, **z** is the relative energy structure sequence number predicted according to the M06-L method, **n** and **m** are the sizes of each ring, and **X** refers to the spin state, with **S, D, T**, and **Q** representing the singlet, doublet, triplet, and quartet spin states, respectively.

## 3 Results and Discussion

### 3.1 C_12_H_12_M Sandwich Compounds of Early Transition Metals with Fully Bonded Planar Rings

The early transition metals of Groups 4–6 in C_12_H_12_M sandwich compounds with fully bonded rings involving all carbon atoms have electronic configurations not exceeding the favored 18-electron configuration. Furthermore, their formal oxidation states in the mixed sandwich compounds (η^5^-C_5_H_5_)­M­(η^7^-C_7_H_7_) **(M1–57S)** are the favorable +4, particularly for the Group 4 metals titanium, zirconium, and hafnium, with the C_7_H_7_^3–^ ring as the 10π-electron trianion and the C_5_H_5_^–^ ring as the 6π-electron monoanion. These **M1–57S** sandwiches are favored energetically by wide margins over their dibenzene-metal isomers (η^6^-C_6_H_6_)_2_M **(M2–66S**) in which the central metal atom is in a formal zero oxidation state (Figure 2). Furthermore, the 16-electron metal configuration of the **M2–66** sandwiches conveys Lewis acidic properties on such species toward a variety of phosphine, isocyanide, and heterocyclic carbene-based ligands to give (η^6^-C_6_H_6_)_2_ML adducts, in which the central metal atom has the favored 18-electron configuration.

**Figure 2:**
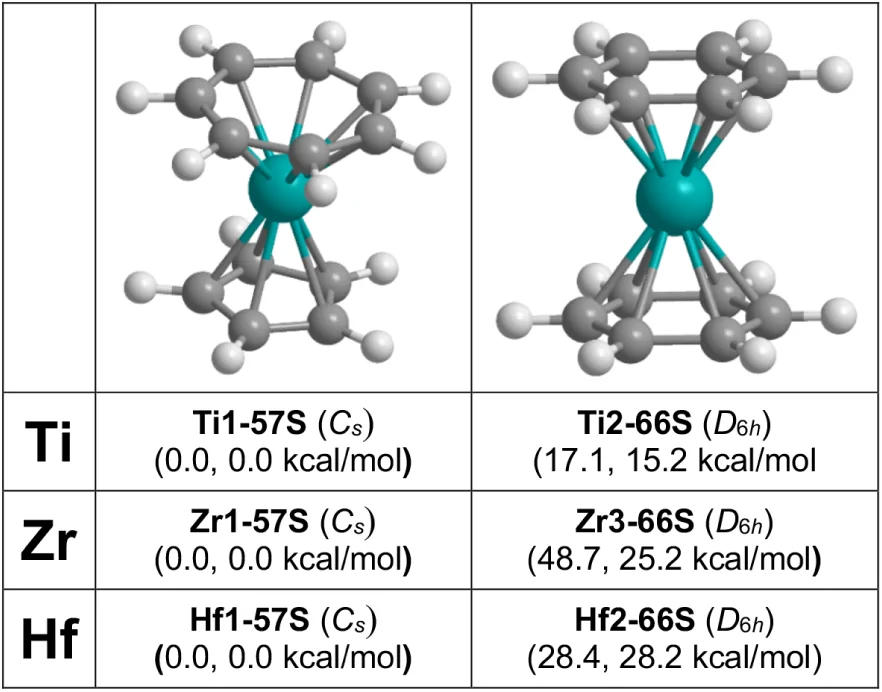
Lowest-energy C_12_H_12_M (M = Ti, Zr, Hf) sandwich structures and their point-group symmetries. The structure figures are the zirconium derivatives. The relative energies (Δ*E*) in kcal/mol were obtained by the M06-L and B3PW91 methods, in that order.

Experiments with metal vapor synthesis are consistent with this interpretation.^17^ Thus, co-condensation of zirconium and hafnium vapors with benzene or toluene in a positive-hearth electron-gun furnace is reported to give deep red solutions, presumably the corresponding bis-arene metal sandwich complexes. However, they appear to be very unstable, decomposing to free metal above −100 °C. The bis-arene metal sandwich complexes can be trapped as their trimethylphosphine adducts (η^6^-RC_6_H_5_)_2_MPMe_3_ (R = H, Me; M = Zr, Hf) having the favored 18-electron metal configuration by co-condensing the metal vapors with a mixture of the arene and trimethylphosphine.

The Group 5 metal sandwiches C_12_H_12_M (M = V, Nb, Ta) are doublet spin-state structures with fully bonded carbocyclic rings and a 17-electron configuration for the central metal atom (Figure 3). The mixed sandwich compounds (η^5^-C_5_H_5_)­M­(η^7^-C_7_H_7_) (M = V, Nb, Ta) **(M1–57D**), with the metal in a + 4 formal oxidation state, are favored energetically over the dibenzene-metal complexes (η^6^-C_6_H_6_)_2_M **(M2–66D**) with the metal in a zero oxidation state, but by smaller margins than for the corresponding sandwich compounds of the Group 4 metals discussed above.

**Figure 3:**
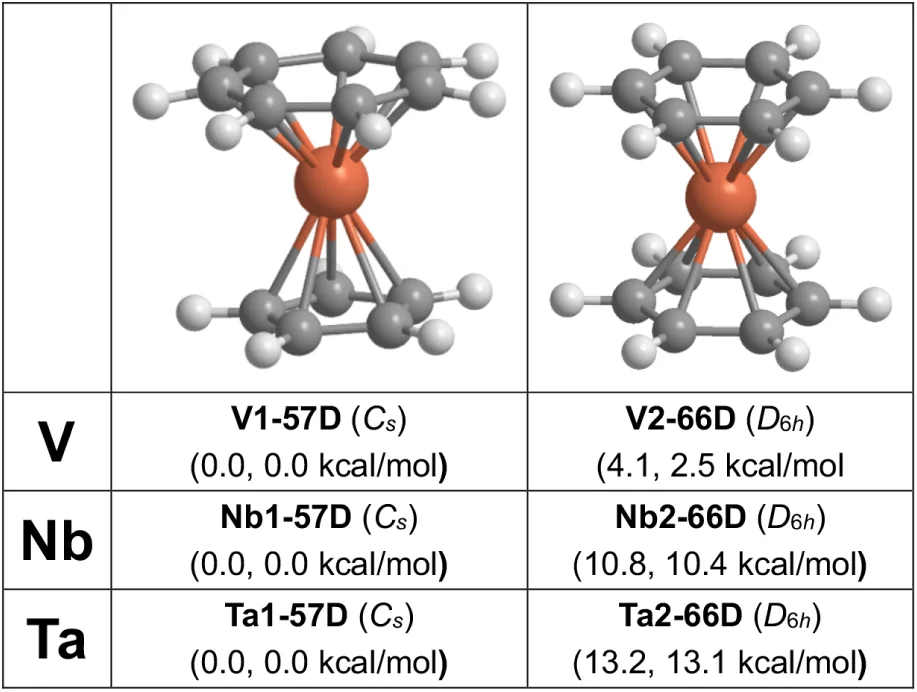
Lowest-energy C_12_H_12_M (M = V, Nb, Ta) structures and their point-group symmetries. The structure figures are the niobium derivatives. The relative energies (Δ*E*) in kcal/mol were obtained by the M06-L and B3PW91 methods, in that order.

The situation changes for the Group 6 metal sandwiches C_12_H_12_M (M = Cr, Mo, W), in which the central metal atom has the favored 18-electron configuration (Figure 4). For these metals, the zerovalent dibenzene-metal derivatives (η^6^-C_6_H_6_)_2_M **(M1–66S**) are favored energetically over their mixed sandwich isomer compounds (η^5^-C_5_H_5_)­M­(η^7^-C_7_H_7_) **(M2–57S)**, albeit by very small margins of ∼4 kcal/mol or less for the molybdenum and tungsten derivatives. This may relate to some difficulty in the smaller Group 6 metal atoms, relative to the neighboring Group 5 metal atoms, in forming strong bonds with all seven carbon atoms of the η^7^-C_7_H_7_ ring.

**Figure 4:**
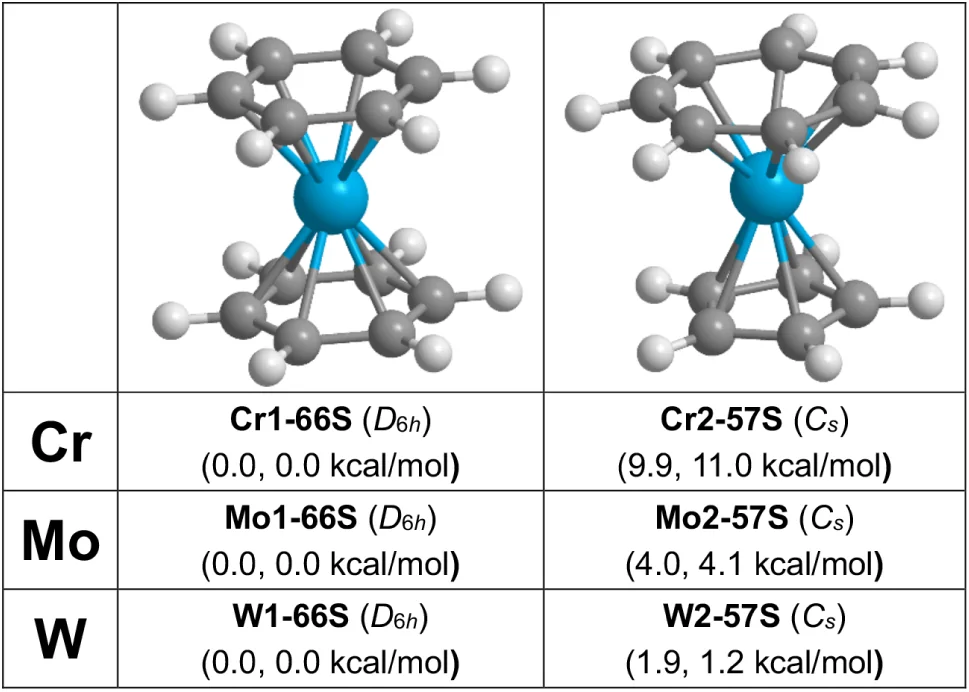
Lowest-energy C_12_H_12_M (M = Cr, Mo, W) structures and their point-group symmetries. The structure figures are the molubdenum derivatives. The relative energies (Δ*E*) in kcal/mol were obtained by the M06-L and B3PW91 methods, in that order.

### 3.2 C_12_H_12_M Sandwiches of the Central Transition Metals in Groups 7 and 8: The Greatest Differences between the First-Row Transition Metals and the Second- and Third-Row Transition Metals

The central metal atom in C_12_H_12_M (M = Mn, Tc, Re) sandwiches of the Group 7 metals with fully bonded carbocyclic rings has a 19-electron configuration, similar to that of the very stable cobaltocene, (η^5^-C_5_H_5_)_2_Co. For the second- and third-row Group 7 metals, the lowest energy structure is the fully bonded doublet spin state dibenzene-metal derivative (η^6^-C_6_H_6_)_2_M (M = Tc, Re) **(M1–66D**) with a 19-electron configuration similar to that of cobaltocene (Figure 5). The lowest energy structures of the mixed sandwiches **(M2–57D**) are doublet spin state structures of the type (η^5^-C_5_H_5_)­M­(η^5^-C_7_H_7_) (M = Tc, Re) in which the seven-membered ring is bent, bonding to the central metal atom through five adjacent carbon atoms, leaving an uncomplexed C=C double bond of length ∼1.32 Å. In these structures, the central metal atom has a 17-electron configuration.

**Figure 5:**
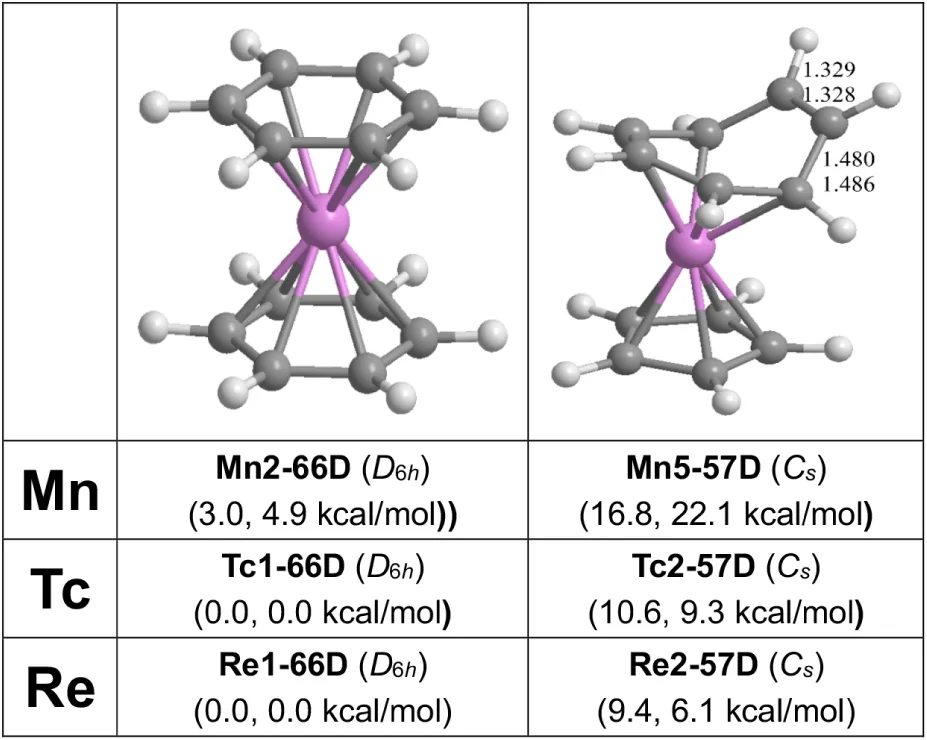
Lowest-energy C_12_H_12_M (M = Mn, Tc, Re) structures and their point-group symmetries. The structure figures are the rhenium derivatives. The relative energies (Δ*E*) in kcal/mol were obtained by the M06-L and B3PW91 methods, in that order.

The lower ligand field strength in the first-row Group 7 metal manganese is reflected in quartet spin state structures being of lower energy than doublet spin state structures for both the (C_6_H_6_)_2_Mn and (C_5_H_5_)­Mn­(C_7_H_7_) sandwiches (Figure 5). Thus, the quartet (η^6^-C_6_H_6_)_2_Mn structure lies ∼4 kcal/mol in energy below the corresponding doublet spin state structure **(M2–66D**). For the mixed sandwiches, the quartet (η^5^-C_5_H_5_)­Mn­(η^7^-C_7_H_7_) structure, with all 12 ring carbon atoms bonded to the central manganese atom, lies ∼13 kcal/mol in energy below the doublet mixed sandwich structure (η^5^-C_5_H_5_)­Mn­(η^7^-C_7_H_7_) designated as **Mn5–57D** in Figure 5 since four isomeric C_12_H_12_M structures including **Mn2–66D** were found to be of lower energy.

The lowest-energy structures for the ruthenium and osmium C_12_H_12_M (M = Ru, Os) sandwiches are singlet structures with 10 of the 12 ring carbons bonded to the central metal atom to give the central metal atom the favored 18-electron configuration (Figure 6). This leaves an uncomplexed C=C double bond of length ∼1.32 Å. The (η^6^-C_6_H_6_)­M­(η^4^-C_6_H_6_) (M = Ru, Os) complexes **(M2–66S**) have been synthesized by co-condensation of their metal vapors with benzene.^28^ Their hexahapto-tetrahapto structures are consistent with their proton NMR spectra. Furthermore, the hexahapto-tetrahapto structure of the more stable hexamethylbenzene derivative (η^6^-Me_6_C_6_)­Ru­(η^4^-Me_6_C_6_) has been confirmed by X-ray crystallography.^26^ The relative energies of the singlet dibenzene-metal sandwiches (η^6^-C_6_H_6_)­M­(η^4^-C_6_H_6_) **(M2–66S**) and their mixed sandwich isomers (η^5^-C_5_H_5_)­M­(η^5^-C_7_H_7_) **(M1–57S**) are very similar, with the latter lying only ∼2 kcal/mol below the former.

**Figure 6:**
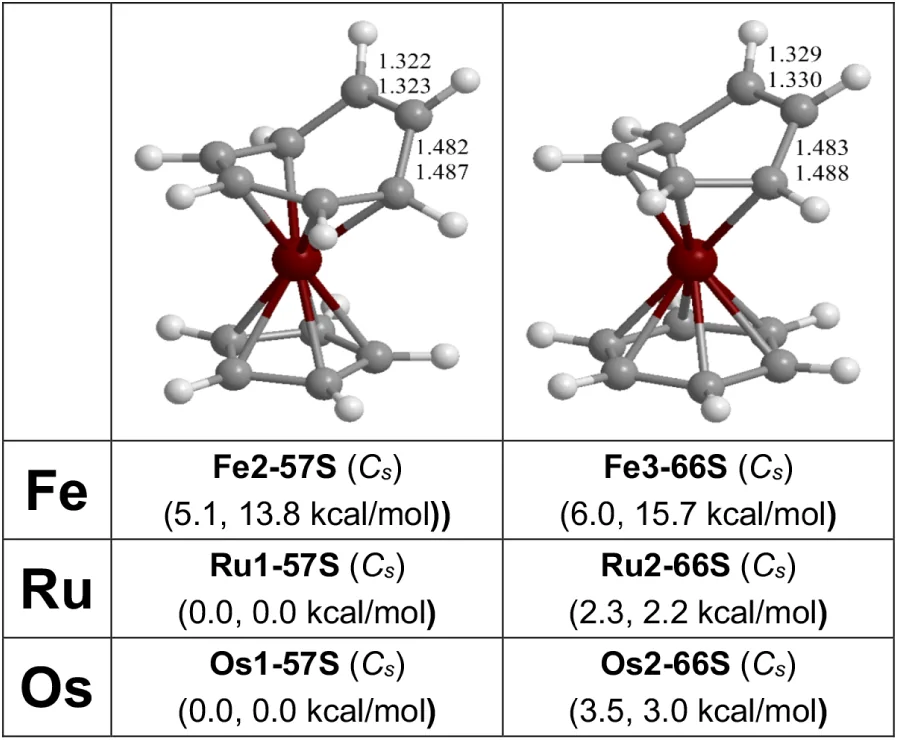
Lowest-energy C_12_H_12_M (M = Fe, Ru, Os) structures and their point-group symmetries. The structure figures are the osmium derivatives. The relative energies (Δ*E*) in kcal/mol were obtained by the M06-L and B3PW91 methods, in that order.

The potential energy surface of the C_12_H_12_Fe sandwich system with a central iron atom is very different from that of its ruthenium and osmium congeners, reflecting the lower ligand fields in iron complexes relative to corresponding ruthenium and osmium complexes. The major difference is the triplet spin state of the lowest-energy C_12_H_12_Fe isomer, namely the dibenzene-iron derivative (η^6^-C_6_H_6_)_2_Fe with two planar hexahapto benzene rings. This leads to a 20-electron iron configuration similar to that in the closely related nickelocene (η^5^-C_5_H_5_)_2_Ni. The neutral, related hexamethylbenzene complex (η^6^-Me_6_C_6_)_2_Fe has been synthesized^29^ by reduction of the dication [(η^6^-Me_6_C_6_)_2_Fe]^2+^. The singlet dibenzene-iron (η^6^-C_6_H_6_)­Fe­(η^4^-C_6_H_6_) isomer **Fe3–66S**, with a nonplanar tetrahapto C_6_H_6_ ring, is a higher-energy structure. The singlet **Fe2–57S** and **Fe2–66S** isomers are of very similar energies, like their ruthenium and osmium analogues.

### 3.3 C_12_H_12_M Sandwiches of the Late Transition Metals in Groups 9 and 10: Unusual Features of the Palladium and Platinum Sandwiches

The lowest-energy C_12_H_12_M sandwich structures for the Group 9 transition metals are doublet hexahapto-dihapto (η^6^-C_6_H_6_)­M­(η^2^-C_6_H_6_) (M = Co, Rh, Ir) structures for the dibenzene-metal derivatives and doublet pentahapto-trihapto (η^5^-C_5_H_5_)­M­(η^3^-C_7_H_7_) structures for the mixed sandwich compounds (Figure 7). Both structure types have 17-electron configurations for the central metal atom. The mixed sandwich (η^5^-C_5_H_5_)­M­(η^3^-C_7_H_7_) structure becomes increasingly favored when moving from cobalt to rhodium and then to iridium. For cobalt and rhodium, the dibenzene-metal (η^6^-C_6_H_6_)­M­(η^2^-C_6_H_6_) structures are energetically favored over the mixed sandwich (η^5^-C_5_H_5_)­M­(η^3^-C_7_H_7_) structures by ∼14 kcal/mol for cobalt and ∼6 kcal/mol for rhodium. For iridium, the relative energy relationship is reversed, with the mixed sandwich structure lying ∼7 kcal/mol below its dibenzene-iridium isomer.

**Figure 7:**
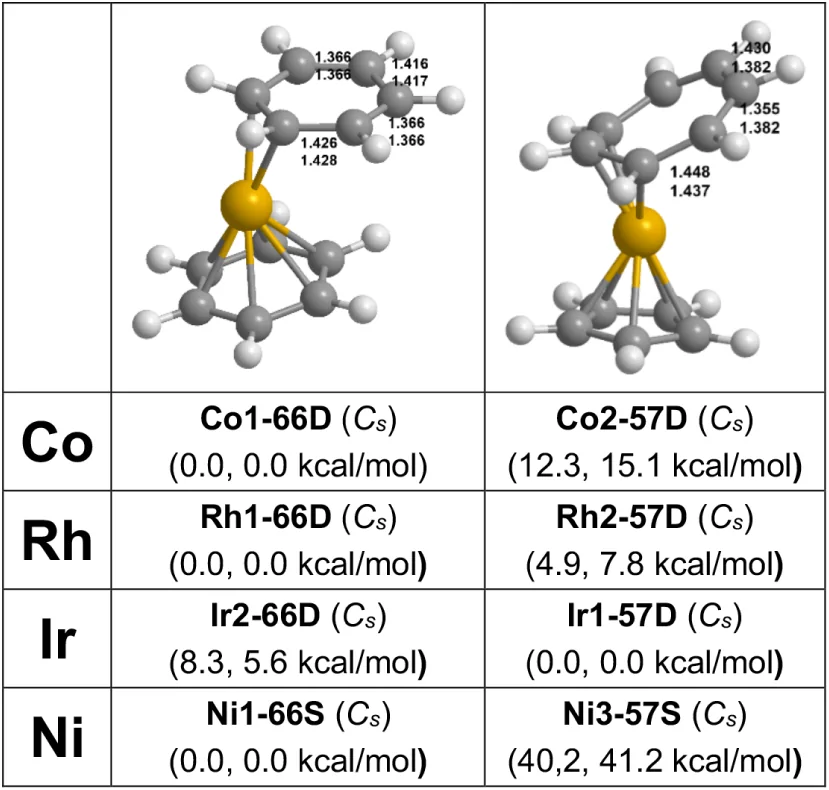
Lowest-energy C_12_H_12_M (M = Co, Rh, Ir, Ni) structures and their point-group symmetries. The structure figures are the rhodium derivatives. The relative energies (Δ*E*) in kcal/mol were obtained by the M06-L and B3PW91 methods, in that order.

For nickel, the situation is similar to that with cobalt, with the hexahapto-dihapto dibenzene-nickel (η^6^-C_6_H_6_)_2_Ni­(η^2^-C_6_H_6_) structure (**Ni1–66S**) being energetically preferred over any other isomer (Figure 7). For nickel, this energetic difference is unusually large, with the lowest-energy mixed sandwich isomer (η^5^-C_5_H_5_)­Ni­(η^3^-C_7_H_7_) **(Ni3–57S**) lying ∼40 kcal/mol above the dibenzene-nickel isomer (η^6^-C_6_H_6_)­Ni­(η^2^-C_6_H_6_) **(Ni1–66S**). The nickel atom in both of these structures has the favorable 18-electron configuration.

The coordination chemistry of palladium and platinum includes square planar metal (II) complexes with a 16-electron metal configuration and linear metal (0) complexes with a 14-electron configuration (Figure 8). The lowest-energy C_12_H_12_M (M = Pd, Pt) sandwich structures **M1–66S** of these metals are bis-dihapto dibenzene-metal (η^2^-C_6_H_6_)_2_M structures, in which each benzene ring, although essentially planar, bonds to the central metal atom through only one of its C=C double bonds. This bonding arrangement leaves an uncomplexed 1,3-butadiene unit in each benzene ring and gives the central two-coordinate metal atom 14-electron linear coordination. A similar (η^2^-C_6_H_6_)_2_Pt structure was found in a previous theoretical study.^42^

**Figure 8:**
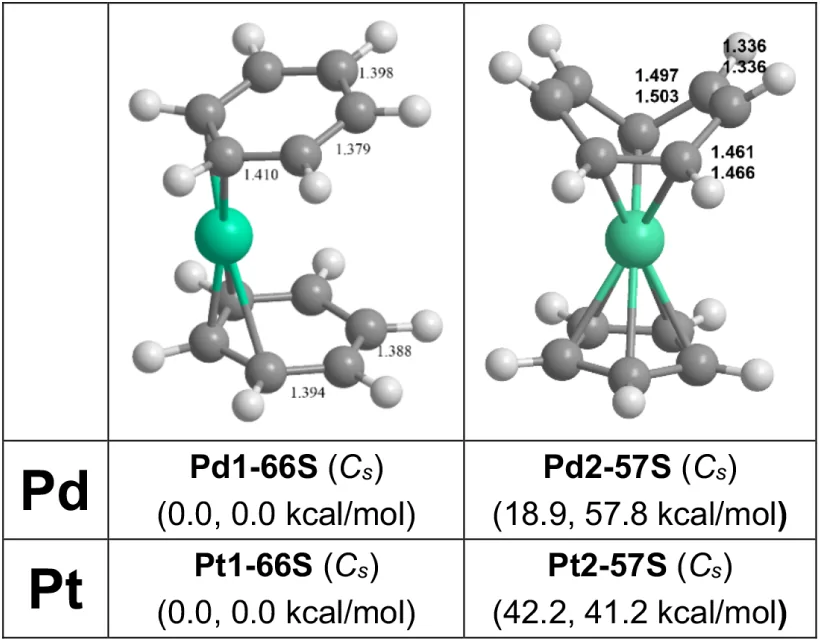
Lowest-energy C_12_H_12_M (M = Pd, Pt) structures and their point-group symmetries. The structure figures are the palladium derivatives. The relative energies (Δ*E*) in kcal/mol were obtained by the M06-L and B3PW91 methods, in that order.

The lowest-energy mixed sandwich structures **M2–57S** of palladium and platinum are of the type (η^5^-C_5_H_5_)­M­(η^2,1^-C_7_H_7_), with an unusual trihapto C_7_H_7_ ligand in which the three carbon atoms bonded to the central metal atom include two of a C=C double bond and a third carbon atom not adjacent to this double bond (Figure 8). This leaves two uncomplexed, nonadjacent C=C double bonds in the C_7_H_7_ ligand with C=C distances of ∼1.35 Å. These **M2–57S** structures lie at high energies of at least ∼20 kcal/mol above the isomeric dibenzene-metal structures **M1–66S**.

## 4 Conclusion

The greatest difference between the preferred C_12_H_12_M sandwich structures for the first-row transition metals and those of the second- and third-row transition metals occurs with the central Group 7 and 8 metals, with the possibility of the largest number of unpaired electrons in high-spin structures for the low ligand-field first-row metals. Thus, the doublet sandwich (η^6^-C_6_H_6_)_2_M and (η^5^-C_5_H_5_)­M­(η^5^-C_7_H_7_) (M = Tc, Re) structures are clearly the lowest-energy structures for the second- and third-row Group 7 metals, technetium and rhenium. However, for the first-row Group 7 metal manganese, the lowest-energy C_12_H_12_Mn structure is a quartet (η^6^-C_6_H_6_)_2_Mn structure. Furthermore, quartet and even sextet C_12_H_12_Mn structures are found lower in energy than the lowest-energy doublet mixed sandwich structure.

A related situation occurs with the Group 8 metals, reflecting the lower ligand fields in iron complexes relative to analogous complexes of ruthenium and osmium. Thus, the singlet structures (η^6^-C_6_H_6_)­M­(η^4^-C_6_H_6_) and (η^5^-C_5_H_5_)­M­(η^5^-C_7_H_7_) (M = Ru, Os), with favored 18-electron configurations and partially bonded nonplanar rings, are energetically preferred for the second- and third-row transition metals ruthenium and osmium. However, the lowest-energy structure for the C_12_H_12_Fe sandwiches is a triplet (η^6^-C_6_H_6_)_2_Fe structure with two fully bonded planar rings and a 20-electron iron configuration. This structure has been realized experimentally in its completely methylated derivative derived from hexamethylbenzene.

The energetically preferred structures for the palladium and platinum sandwiches C_12_H_12_M (M = Pd, Pt) are very different from those preferred for the nickel C_12_H_12_Ni sandwiches. However, this does not appear to be a consequence of differences in ligand field strength. Instead, it is related to the tendency of palladium and platinum to form complexes with low-coordination numbers and 16- and 14-electron configurations. Thus, for the C_12_H_12_M (M = Pd, Pt), the preferred structures are dibenzene-metal (η^2^-C_6_H_6_)_2_M structures in which each benzene ring is a dihapto ligand bonded to the metal atom through only one of its three C=C double bonds. The central metal atom thus is two-coordinate, with a linear configuration of the ligands and a 14-electron configuration. The lowest-energy C_12_H_12_Ni sandwich structure is also a singlet dibenzene-metal complex but of the type (η^6^-C_6_H_6_)­Ni­(η^2^-C_6_H_6_) with one hexahapto and one dihapto benzene ligand to give the nickel atom the normally favored 18-electron configuration. Similar doublet (η^6^-C_6_H_6_)­M­(η^2^-C_6_H_6_) (M = Co, Rh, Ir) structures with 17-electron metal configurations are energetically preferred for the Group 9 metals.

The lowest-energy C_12_H_12_M structures for the early transition metals, namely those of Groups 4, 5, and 6, have all 12 carbon atoms of the two rings bonded to the central metal atom. The (η^5^-C_5_H_5_)­M­(η^7^-C_7_H_7_) structures are energetically preferred over the isomeric (η^6^-C_6_H_6_)_2_M structures for the Group 4 and 5 metals. This energetic difference is relatively large for the Group 4 metals, where the dibenzene-metal complexes have the metal atom in the zero formal oxidation state. This can be related to the experimental observation that these dibenzene-metal complexes cannot be isolated in an uncomplexed form from reactions of the metal vapors with benzene. However, they are formed in such reactions as unstable species that can be trapped as their trimethylphosphine complexes (η^6^-C_6_H_6_)_2_MPMe_3_ (M = Zr, Hf) with 18-electron metal configurations.

## Supplementary Material





## Notes

Figures S1–S7: optimized (C_*n*_H_*n*_)­M­(C_*m*_H_*m*_) (*n* = 6, 5, 4; *m* = 6, 7, 8; *m* + *n* = 12; M = Ti to Ni, Zr to Pd, Hf to Pt) structures; Tables S1–S63: harmonic vibrational frequencies (in cm^–1^) and infrared intensities (in parentheses, in km/mol) for the (C_*n*_H_*n*_)­M­(C_*m*_H_*m*_) (*n* = 6, 5,4; *m* = 6, 7, 8; *m* + *n* = 12; M = Ti to Ni, Zr to Pd, Hf to Pt) structures calculated using the B3PW91 and M06-L methods (PDF)
Coordinates of structures (XYZ)
