# Supplementary material for: Effects of Increased Ligand Fields of the Second- and Third-Row Transition Metals on the Preferred Structures of Sandwich Compounds of Stoichiometry C12H12M
Source: Organometallics. 2026 Jul 31;45(16):2018–24. doi: 10.1021/acs.organomet.6c00202 (PMC13509124; doi:10.1021/acs.organomet.6c00202)
Supplement: Supplementary file 1 [file om6c00202_si_001.pdf]

Supporting Information

Effects of Increased Ligand Fields of the Second and Third Row Transition Metals on the Preferred Structures of Sandwich Compounds of Stoichiometry $C_{12}H_{12}M$

Yongtao Liu, [a] Haoyu Chen,[a] Jinfeng Luo,[a] Huidong Li, [a,b]*Zhixiang Fan,[a]* Qunchao Fan,[a] R. Bruce King, [b]* and Henry F. Schaefer, III [b]

[a] *School of Science, Key Laboratory of High Performance Scientific Computation, Xihua University, Chengdu, China 610039*
[b]*Center for Computational Quantum Chemistry, University of Georgia, Athens, Georgia, USA 30602*

rbking@chem.uga.edu; huidongli@mail.xhu.edu.cn

Figure S1 to S7. Optimized $(C_nH_n)M(C_mH_m)$ (n=6,5,4;m=6,7,8;m+n=12; M=Ti-Ni, Zr-Pd, Hf-Pt) structures

Tables S1 to S63: Harmonic vibrational frequencies (in cm^{-1}) and infrared intensities (in parentheses, in km/mol) for the $(C_nH_n)M(C_mH_m)$ (n=6,5,4;m=6,7,8;m+n=12; M=Ti-Ni, Zr-Pd, Hf-Pt) structures calculated with the B3PW91 method and M06-L method.

Figure S1. Optimized (C_nH_n)Ti(C_mH_m) (n=6,5,4;m=6,7,8) structures

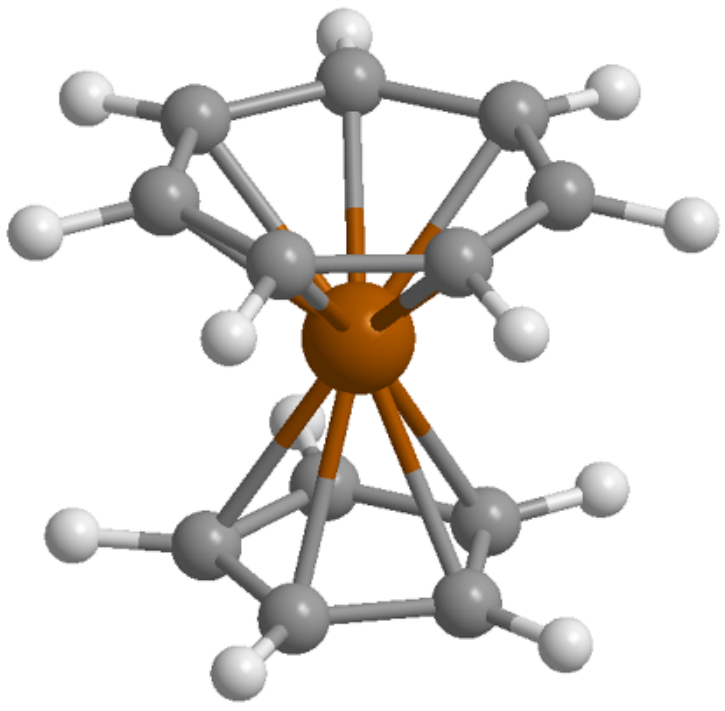<br>57-Ti-1S(C_s)<br>(0.0,0.0)<br>(0.0,0.0)	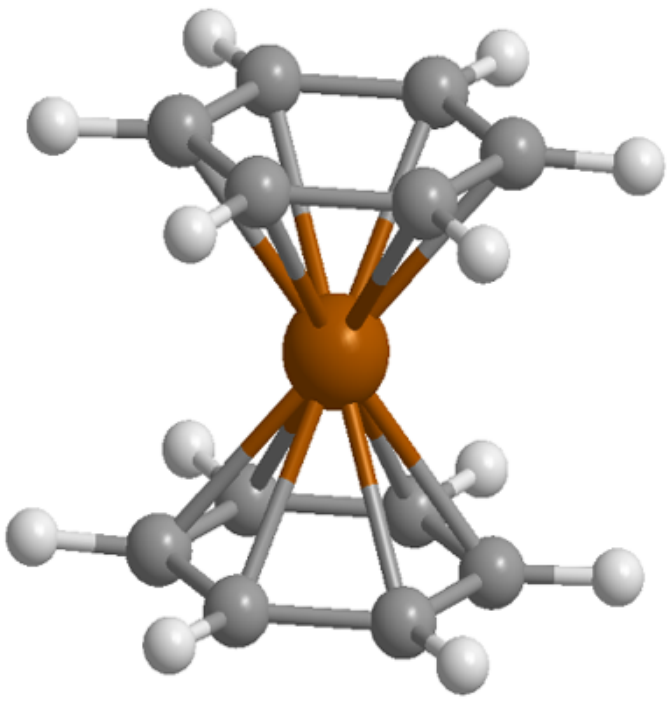<br>66-Ti-2S(D_{6h})<br>(17.1,15.2)<br>(0.0,0.0)	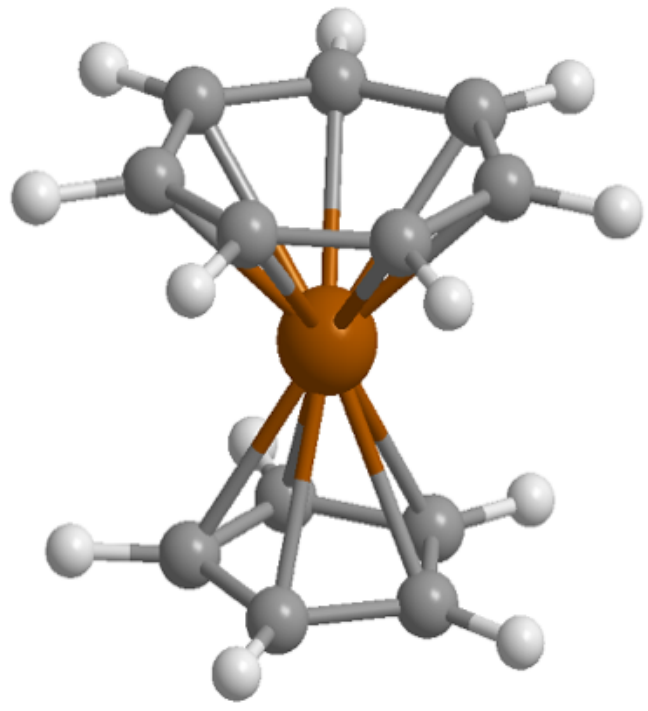<br>57-Ti-3T(C_s)<br>(26.2,21.6)<br>(26.2,21.6)
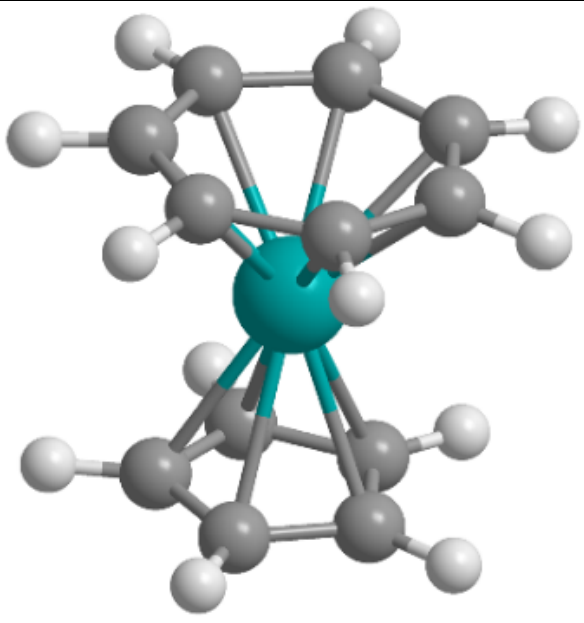<br>57-Zr-1S(C_s)<br>(0.0,0.0)<br>(0.0,0.0)	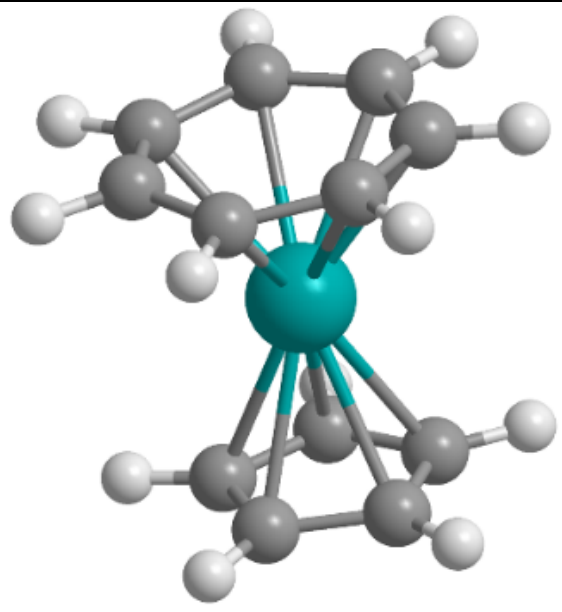<br>57-Zr-2T(C_s)<br>(32.4,34.6)<br>(32.4,34.6)	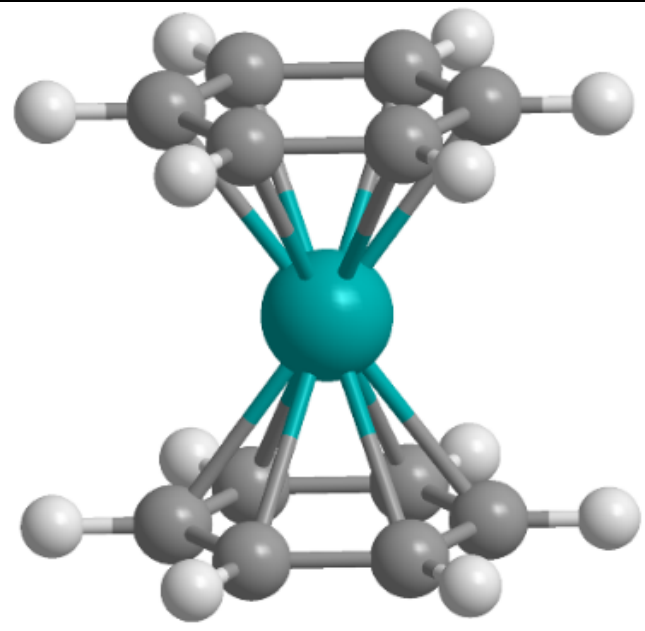<br>66-Zr-3S(D_{6h})<br>(48.7,25.2)<br>(0.0,0.0)
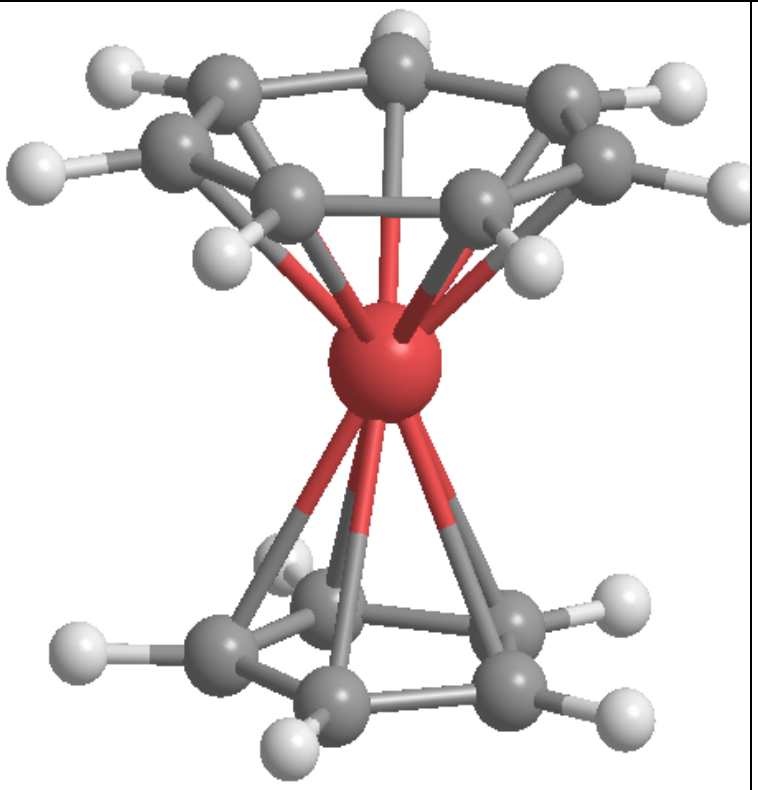<br>57-Hf-1S(C_s)<br>(0.0,0.0)<br>(0.0,0.0)	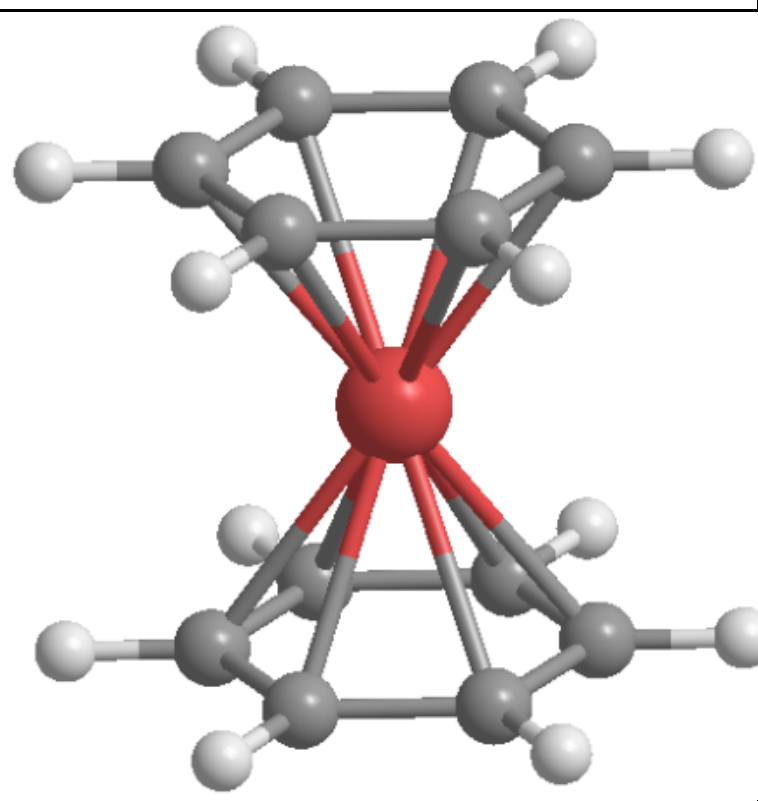<br>66-Hf-2S(D_{6h})<br>(28.4,28.2)<br>(0.0,0.0)	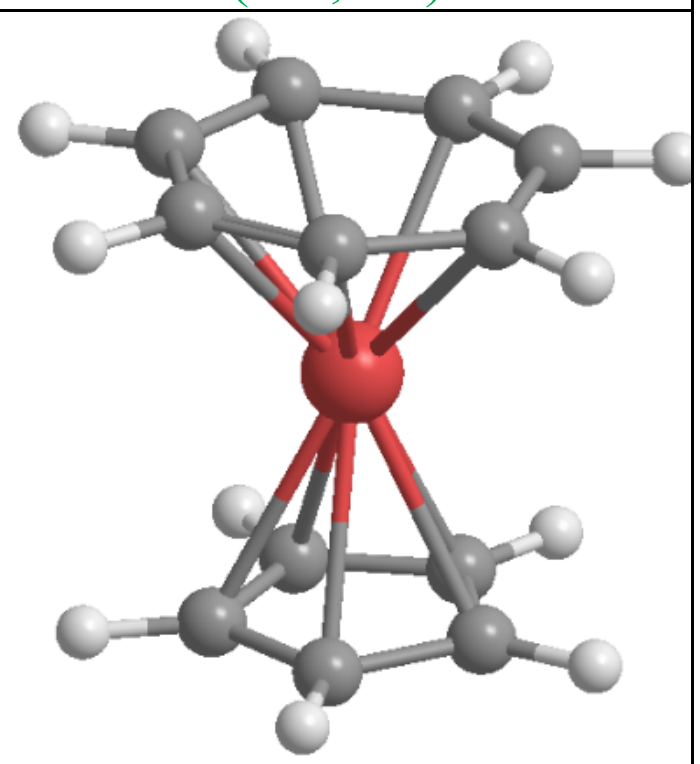<br>57-Hf-3T(C_s)<br>(36.7,37.6)<br>(36.7,37.6)

Figure S2. Optimized (C_nH_n)V(C_mH_m) (n=6,5,4;m=6,7,8) structures

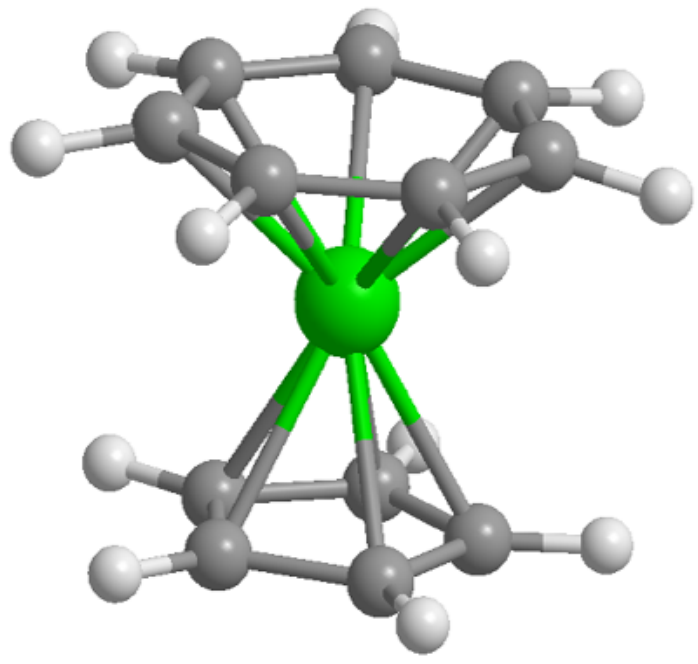<br>57-V-1D(C_s)<br>(0.0,0.0)<br>(0.0,0.0)	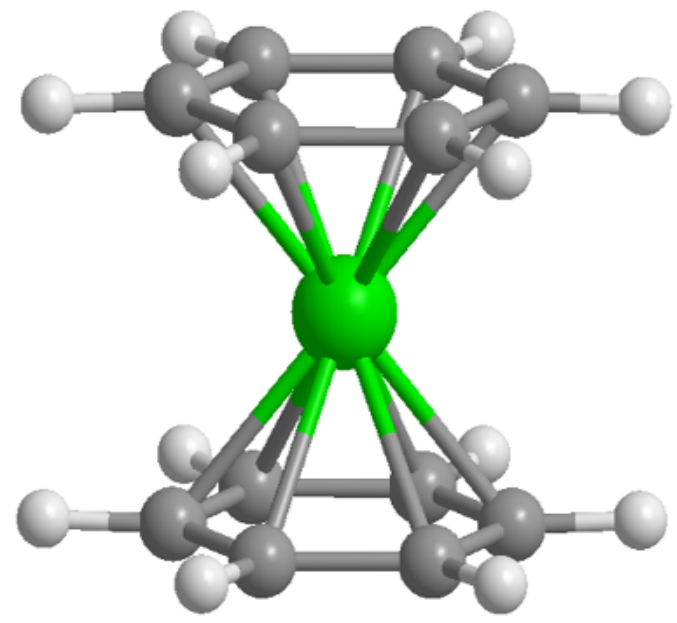<br>66-V-2D(D_{6h})<br>(4.1,2.5)<br>(0.0,0.0)	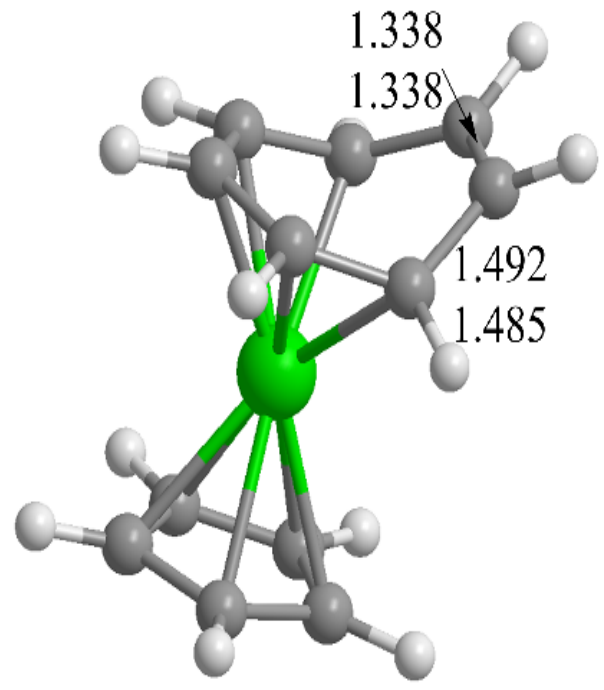<br>57-V-3Q(C_s)<br>(40.8,36.5)<br>(40.8,36.5)
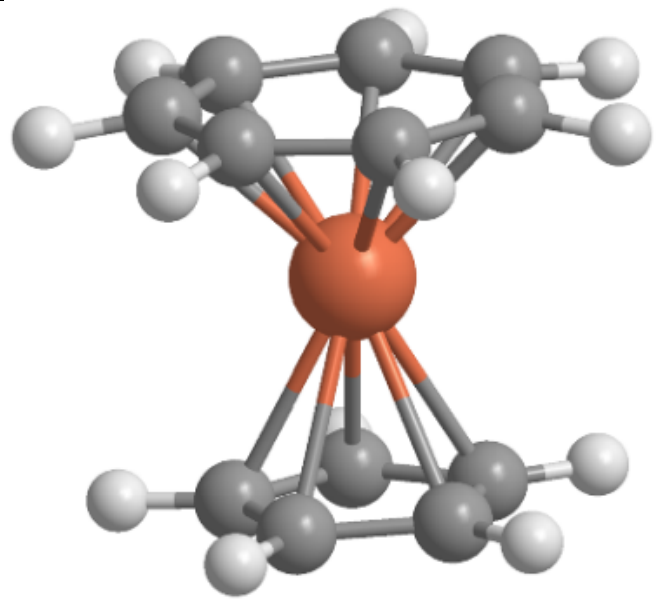<br>57-Nb-1D(C_1,C_s)<br>(0.0,0.0)<br>(0.0,0.0)	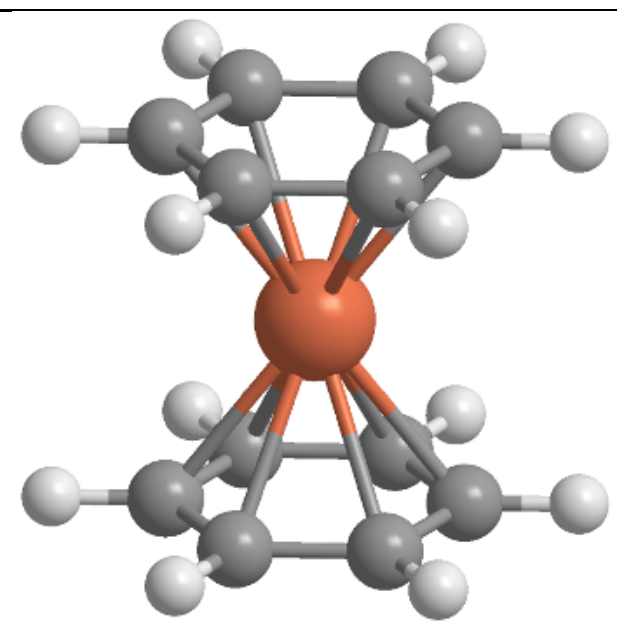<br>66-Nb-2D(D_{6h})<br>(10.8,10.4)<br>(0.0.0.0)	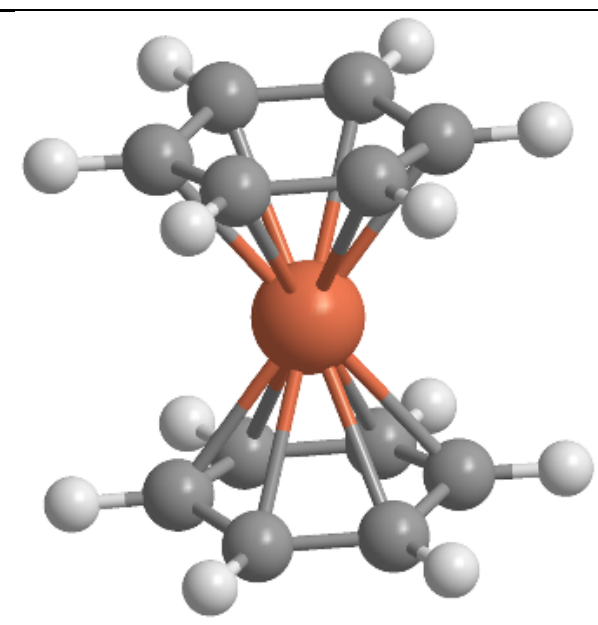<br>66-Nb-3Q(D_{2h})<br>(48.2,51.4)<br>(37.4,41.0)
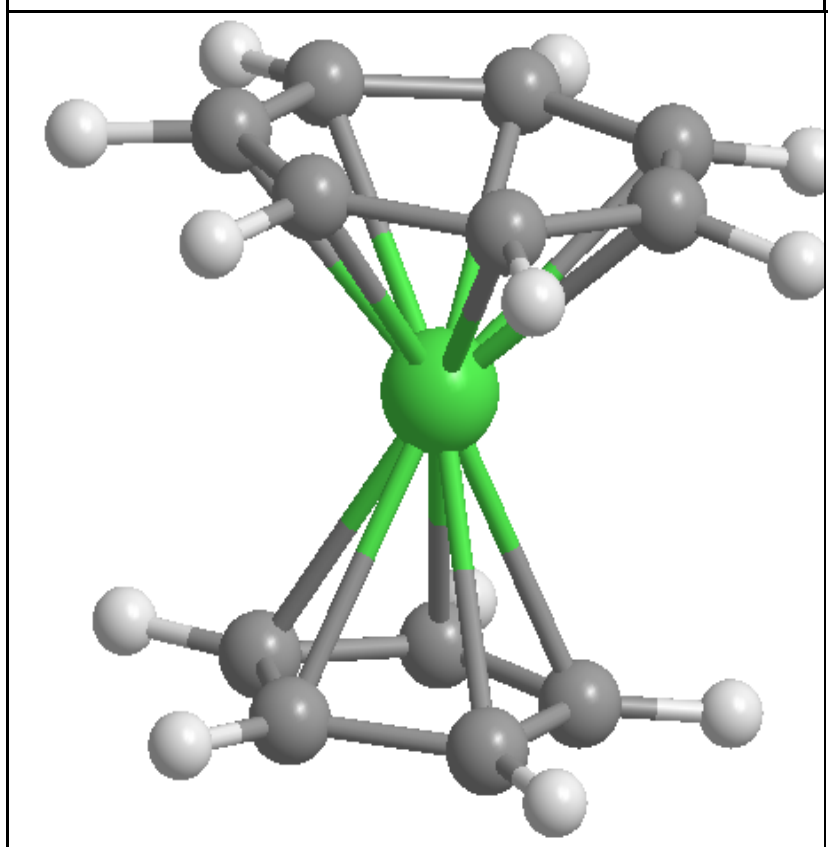<br>57-Ta-1D(C_s)<br>(0.0,0.0)<br>(0.0,0.0)	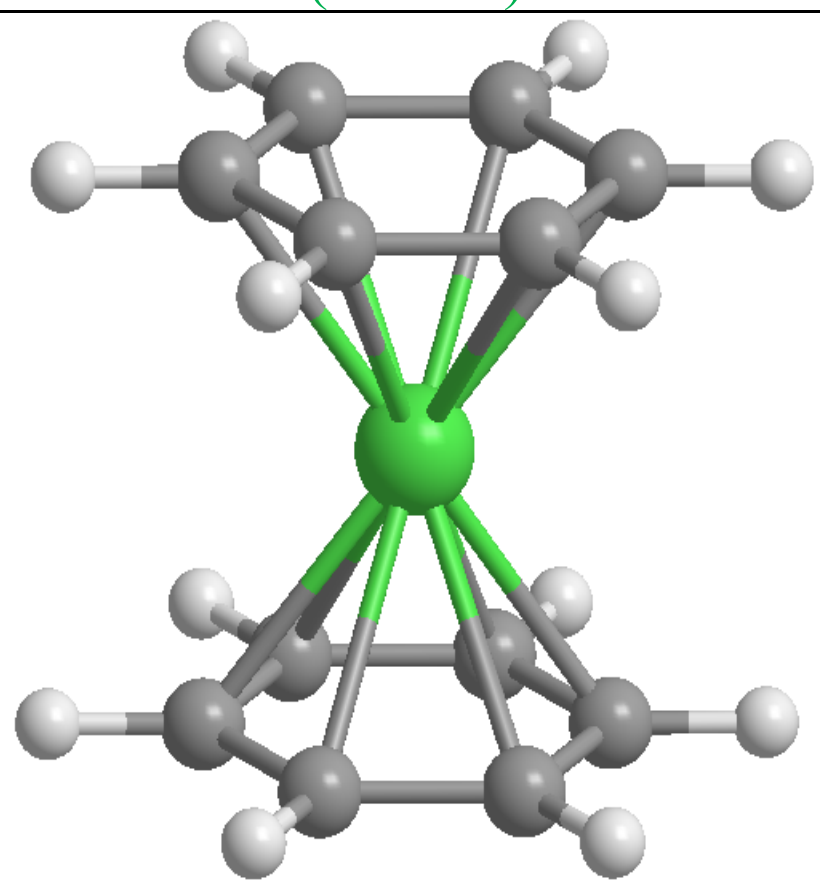<br>66-Ta-2D(D_{6h})<br>(13.2,13.1)<br>(0.0,0.0)	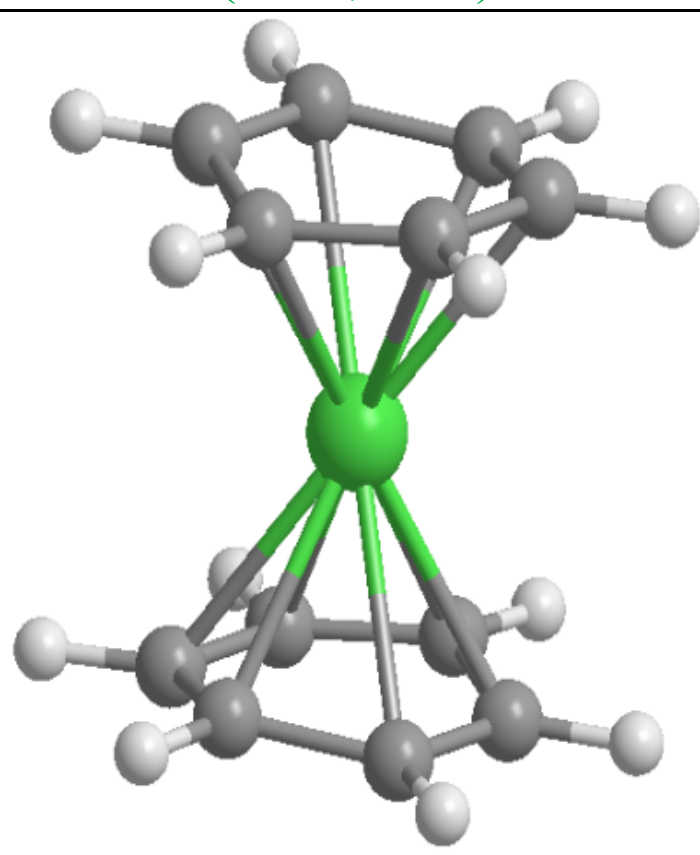<br>66-Ta-3Q(D_{2h})<br>(48.6,51.8)<br>(35.4,38.8)

Figure S3. Optimized $(C_nH_n)Cr(C_mH_m)$ (n=6,5,4;m=6,7,8) structures

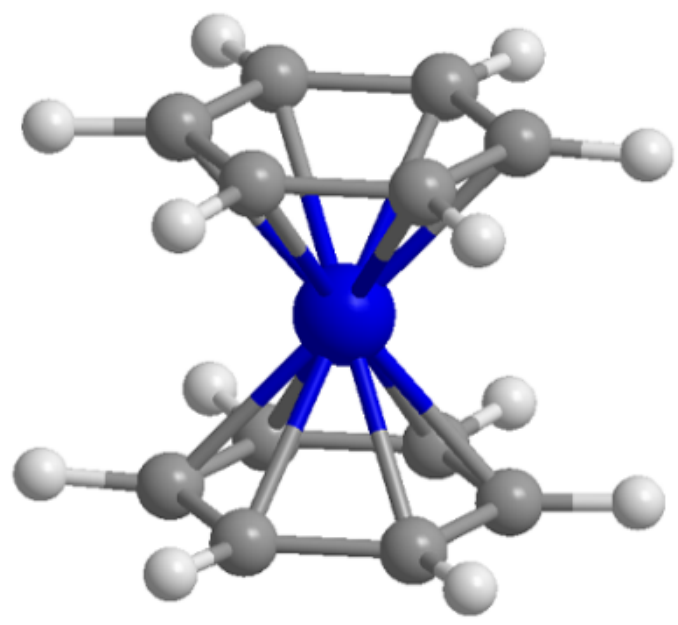

66-Cr-1S(D_{6h})
(0.0,0.0)
(0.0,0.0)

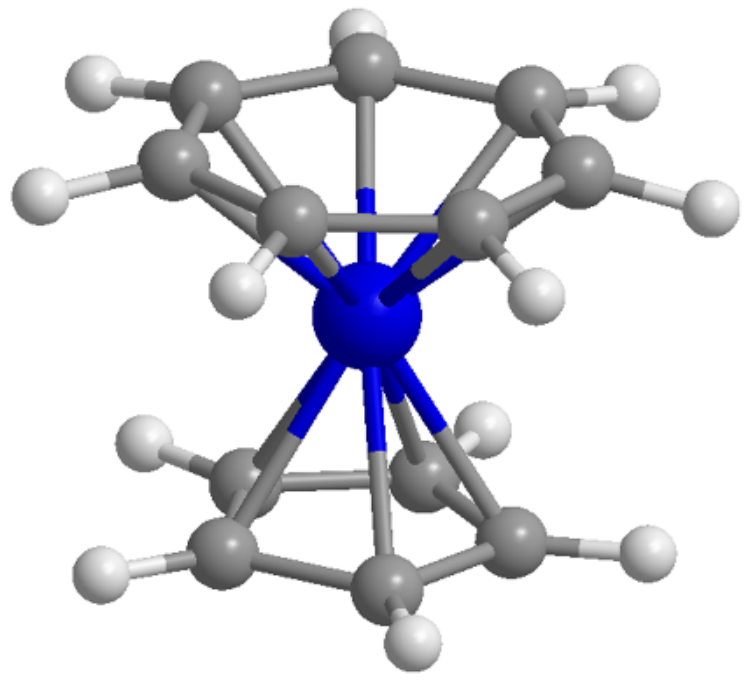

57-Cr-2S(C_s)
(9.9,11.2)
(0.0,0.0)

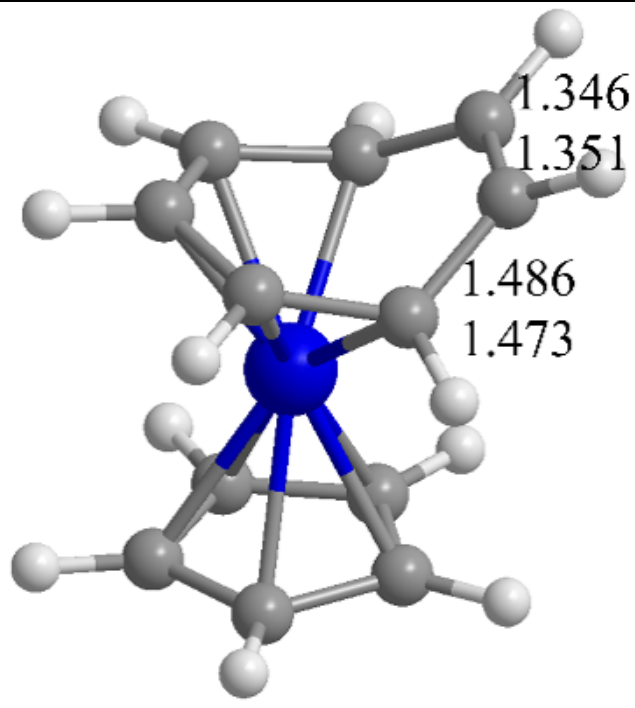

57-Cr-3T(C_s)
(25.7,24.7)
(15.9,13.5)

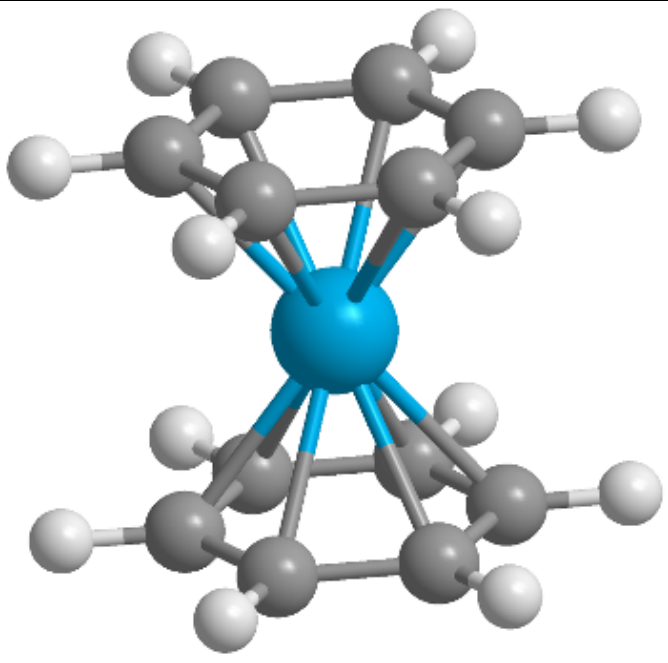

66-Mo-1S(D_{6h})
(0.0,0.0)
(0.0,0.0)

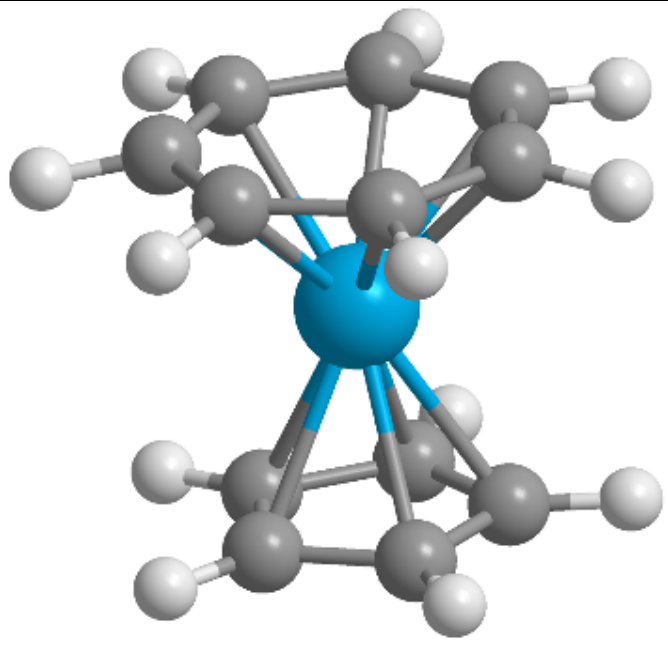

57-Mo-2S(C_s)
(4.0,4.1)
(0.0,0.0)

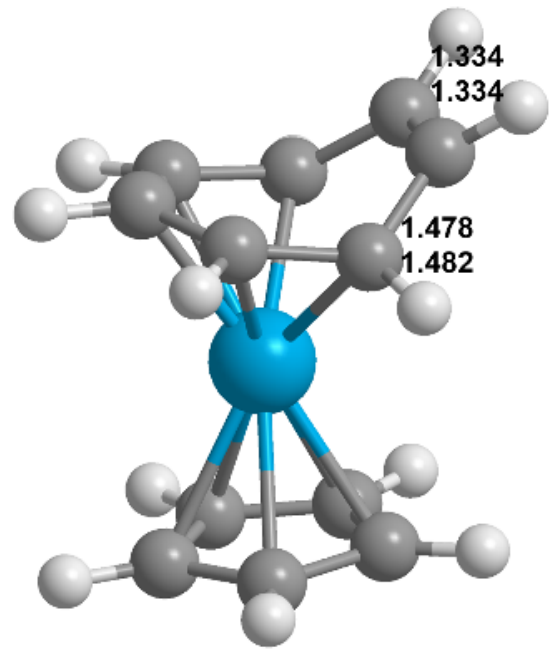

57-Mo-3T(C_s)
(37.7,36.2)
(33.6,32.1)

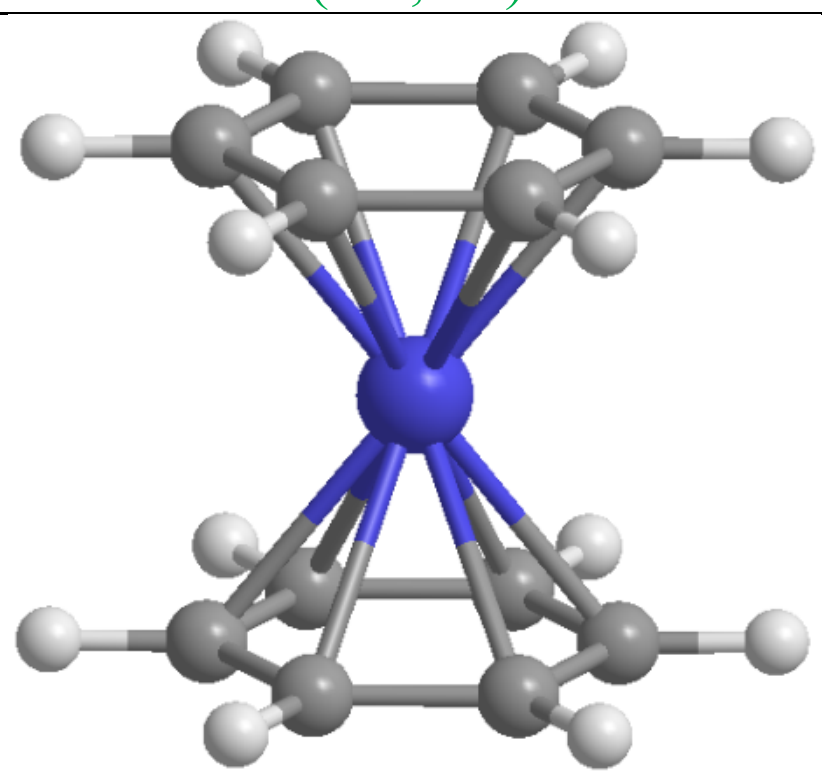

66-W-1S(D_{6h})
(0.0,0.0)
(0.0,0.0)

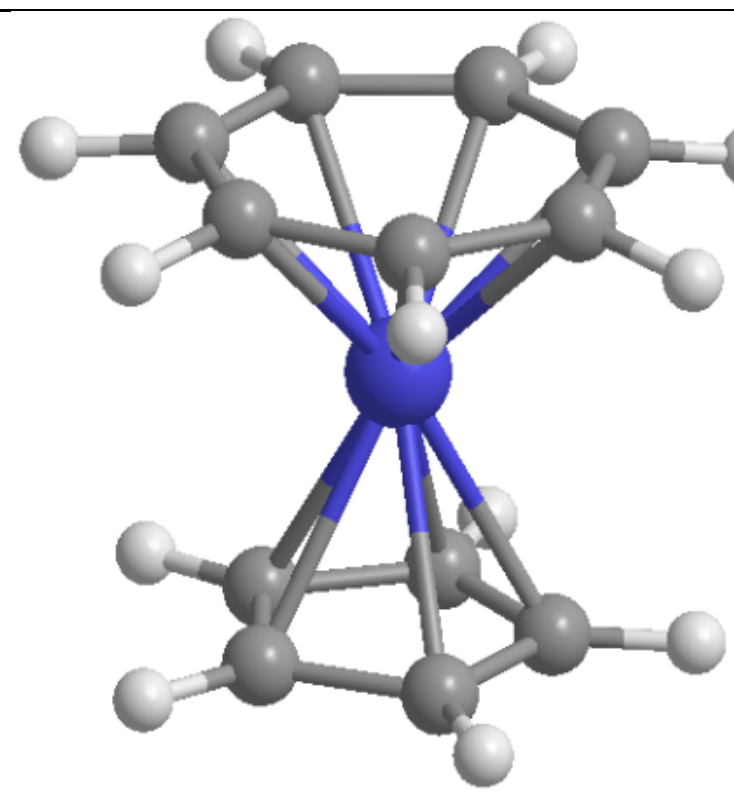

57-W-2S(C_s)
(1.9,1.2)
(0.0,0.0)

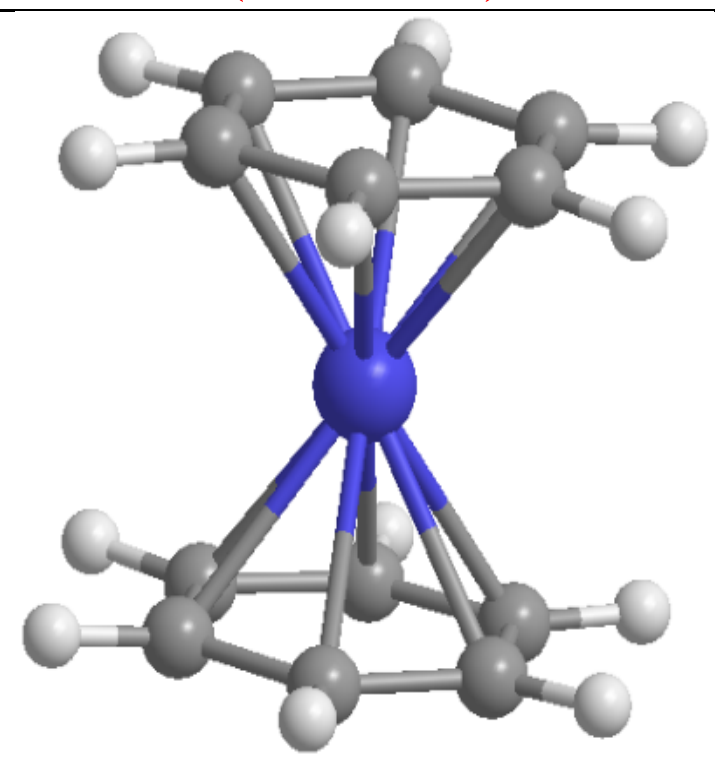

66-W-3T(D_{2h})
(32.2,31.8)
(32.2,31.8)

Figure S4. Optimized $(C_nH_n)Mn(C_mH_m)$ (n=6,5,4;m=6,7,8) structures

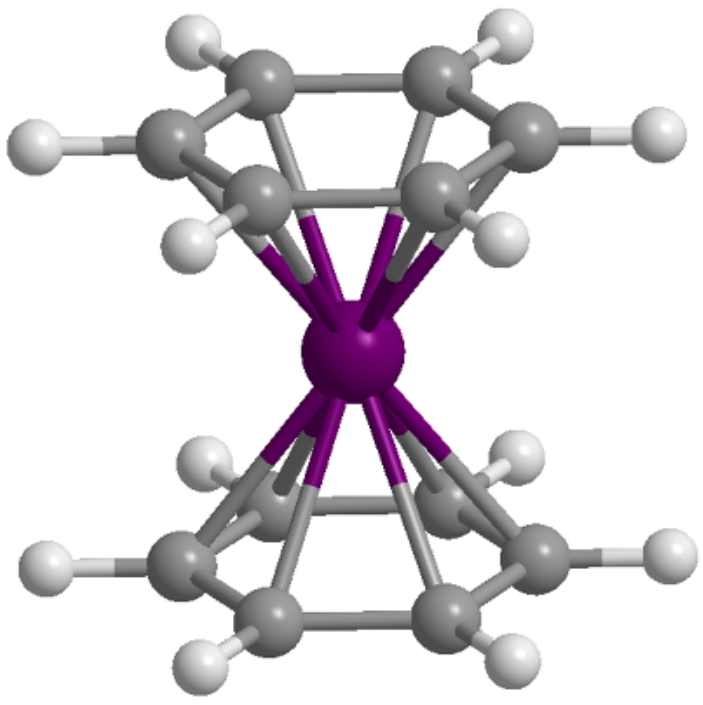 66-Mn-1Q (*D_{6h}*) (0.0,0.0) (0.0,0.0)	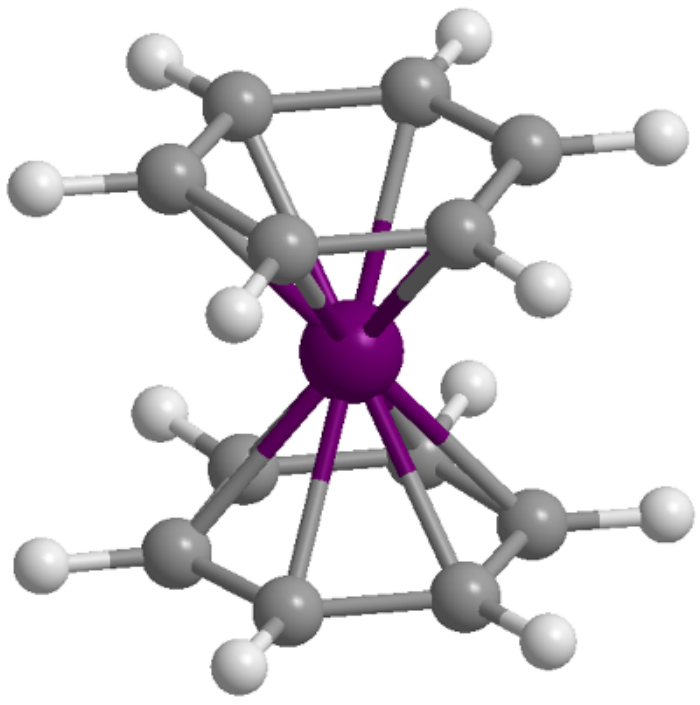 66-Mn-2D(*C_1*) (3.0,4.9) (3.0,4.9)	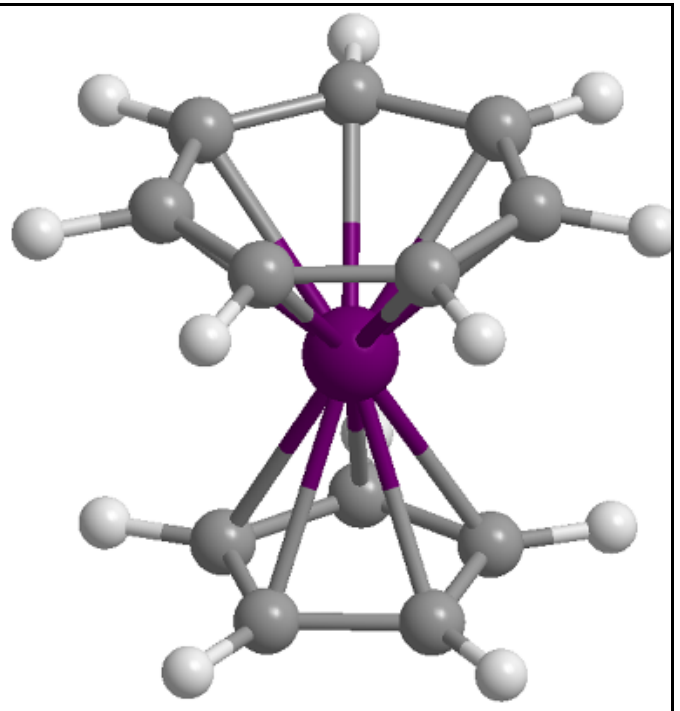 57-Mn-3Q(*C_s*) (5.1,6.0) (0.0,0.0)
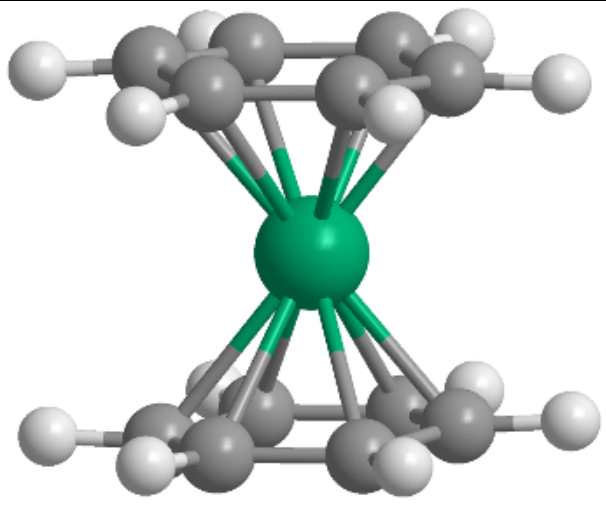 66-Tc-1D(*C_{2v}*) (0.0,0.0) (0.0,0.0)	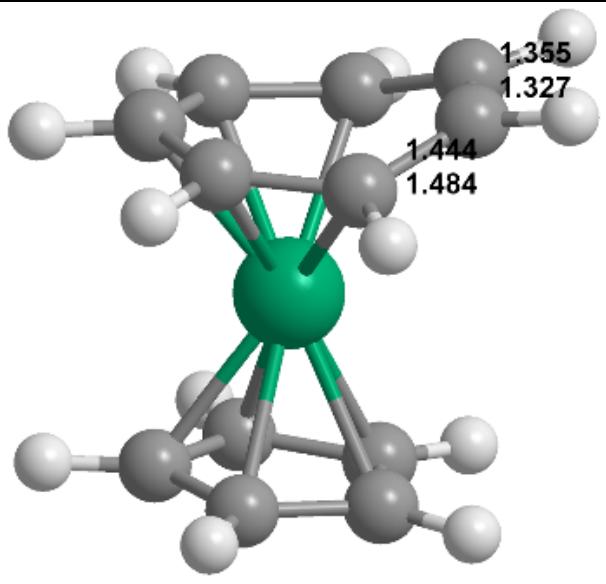 57-Tc-2D(*C_1*) (10.6,9.3) (0.0,0.0)	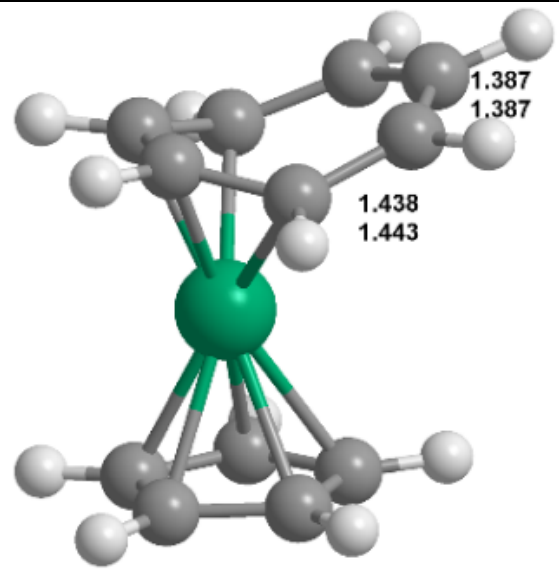 57-Tc-3Q(*C_s*) (33.4,32.4) (22.8,23.1)
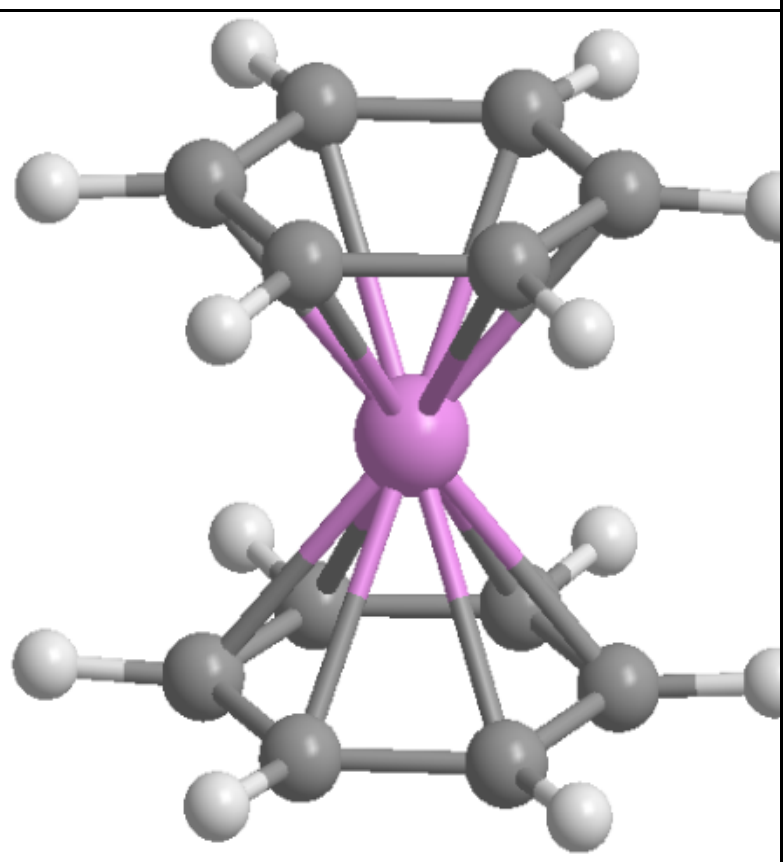 66-Re-1D(*D_{6h}*) (0.0,0.0) (0.0,0.0)	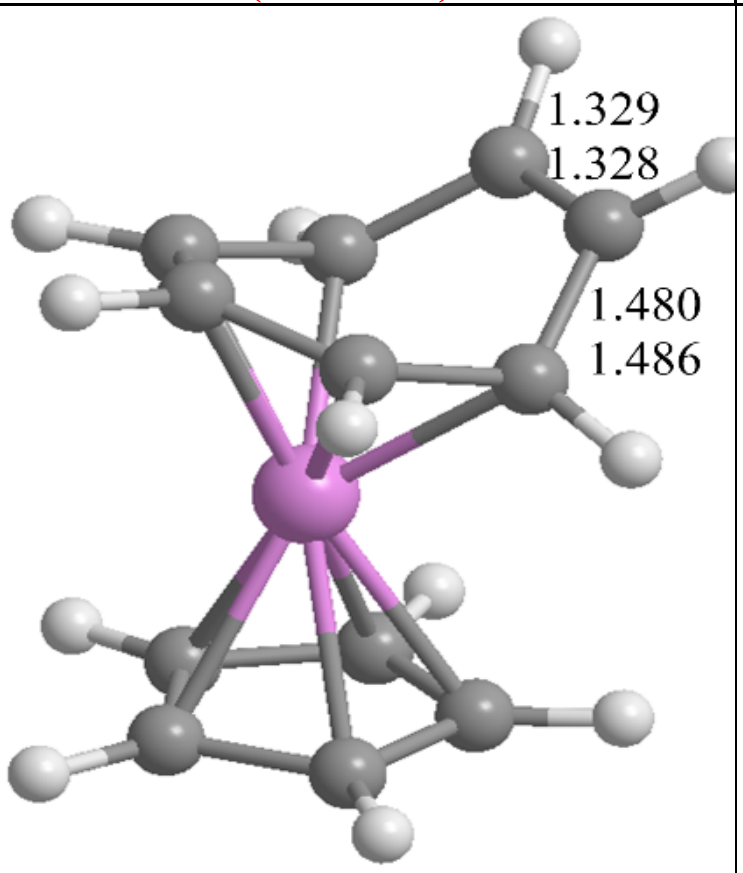 57-Re-2D(*C_s*) (9.4,6.1) (0.0,0.0)	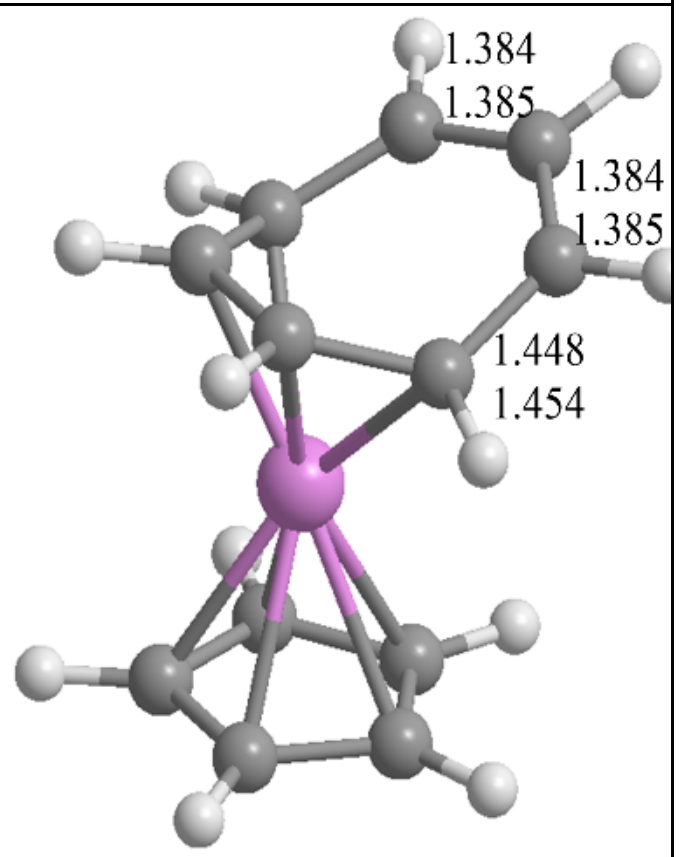 57-Re-3Q(*C_s*) (35.9,34.3) (26.5,28.2)

Figure S5. Optimized $(C_nH_n)Fe(C_mH_m)$ (n=6,5,4;m=6,7,8) structures

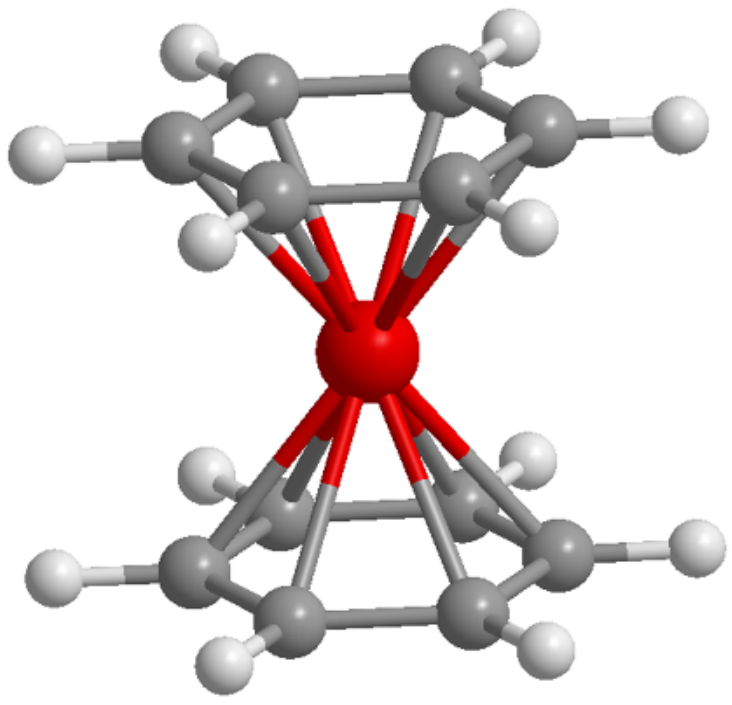<br>66-Fe-1T (D_{6h})<br>(0.0,0.0)<br>(0.0,0.0)	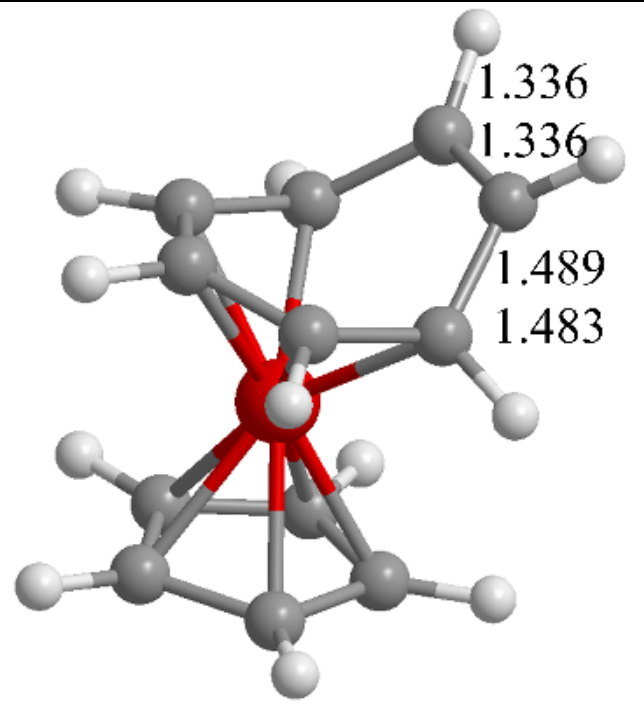<br><br>57-Fe-2S(C_s)<br>(5.1,13.8)<br>(0.0,0.0)	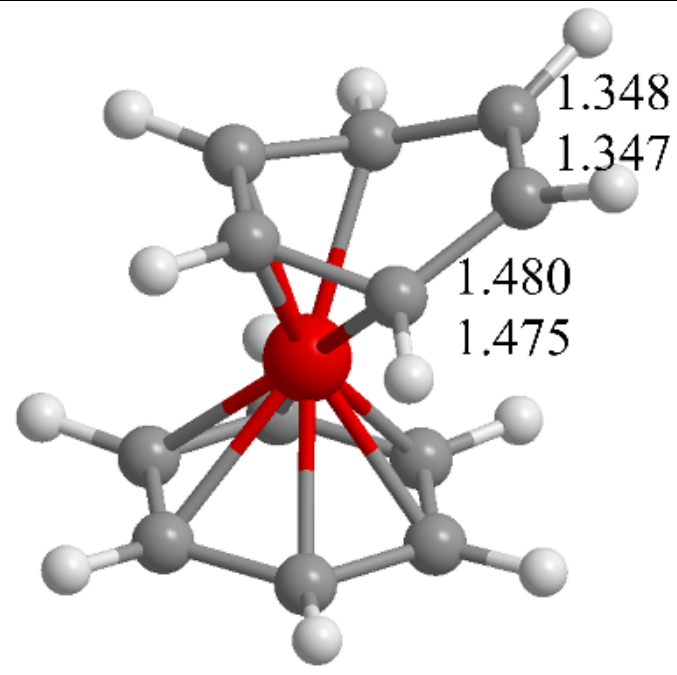<br><br>66-Fe-3S(C_s)<br>(6.0,15.7)<br>(6.0,15.7)
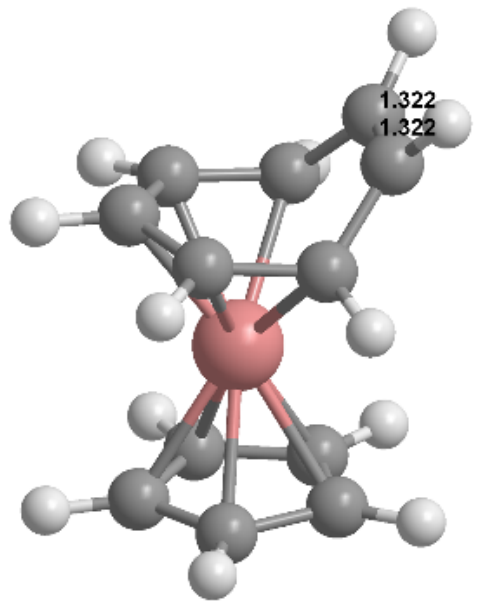<br><br>57-Ru-1S(C_1)<br>(0.0,0.0)<br>(0.0,0.0)	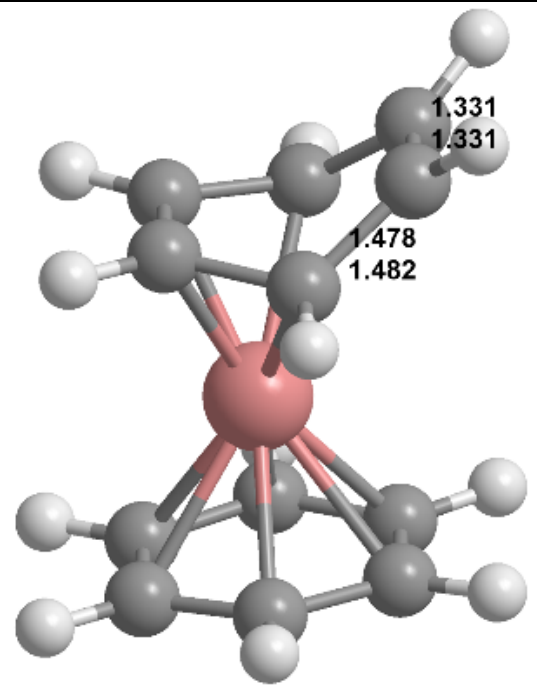<br><br>66-Ru-2S(C_s)<br>(2.3,2.2)<br>(0.0,0.0)	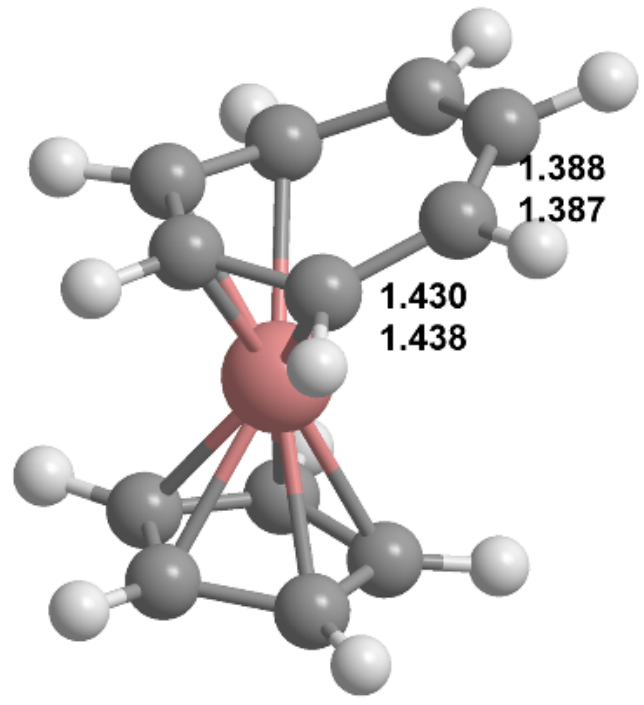<br><br>57-Ru-3T(C_s)<br>(25.5,26.9)<br>(25.5,26.9)
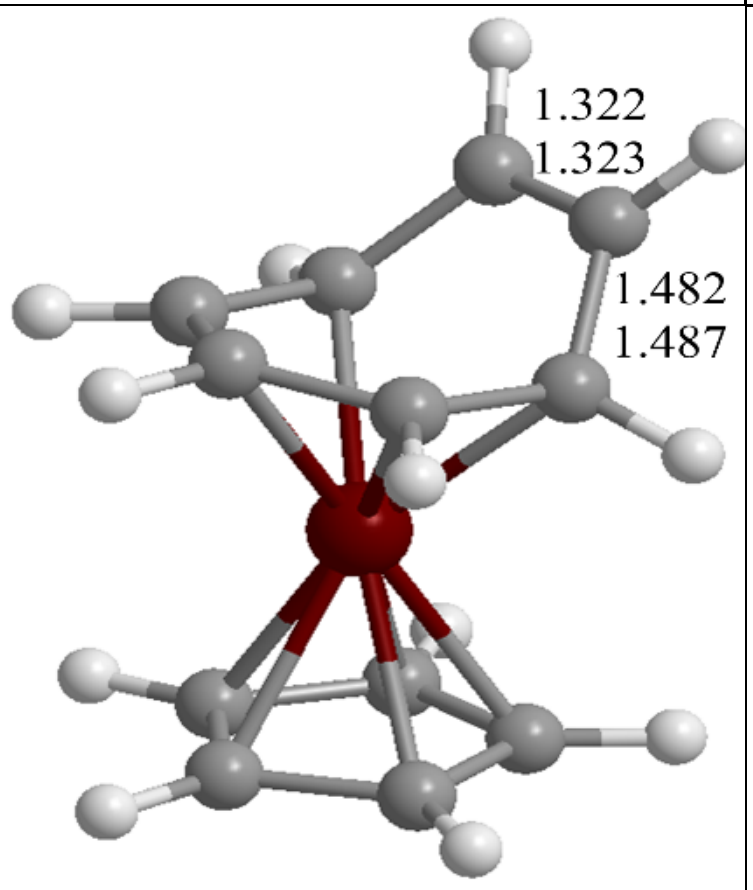<br><br>57-Os-1S(C_s)<br>(0.0,0.0)<br>(0.0,0.0)	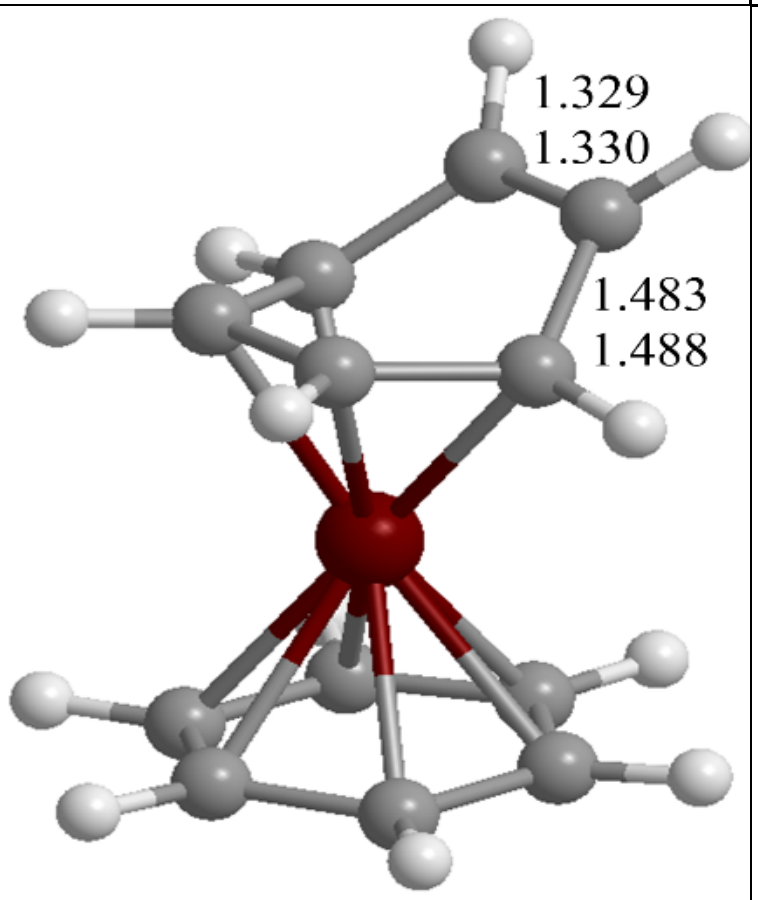<br><br>66-Os-2S(C_s)<br>(3.5,3.0)<br>(0.0,0.0)	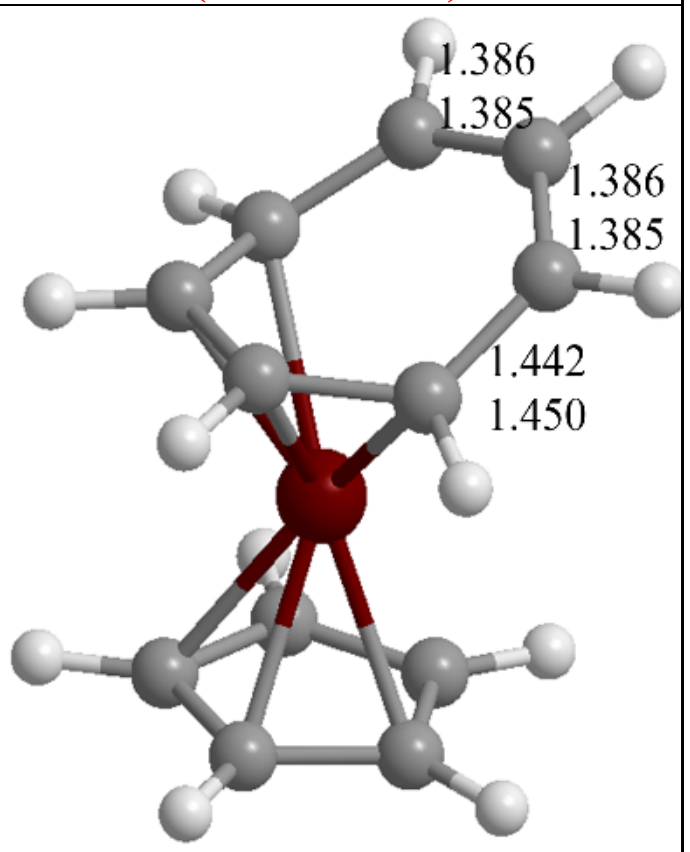<br><br>57-Os-2T(C_s)<br>(30.8,31.6)<br>(30.8,31.6)

Figure S6. Optimized $(C_nH_n)Co(C_mH_m)$ (n=6,5,4;m=6,7,8) structures

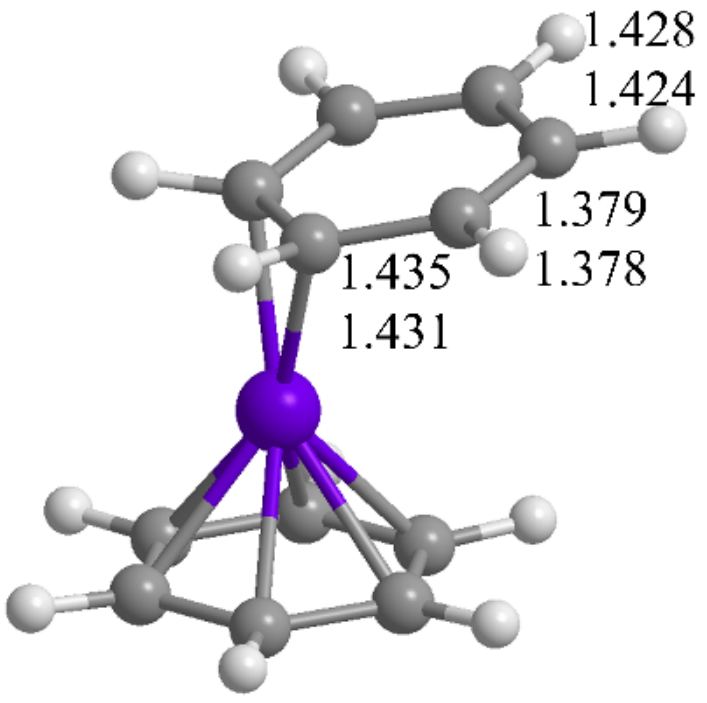

66-Co-1D(C_s)
(0.0,0.0)
(0.0,0.0)

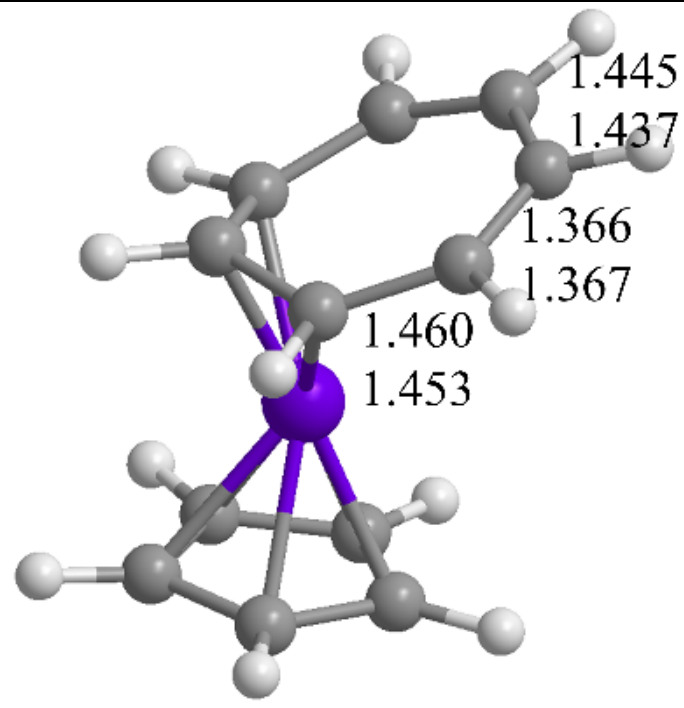

57-Co-2D(C_s)
(12.3,15.1)
(0.0,0.0)

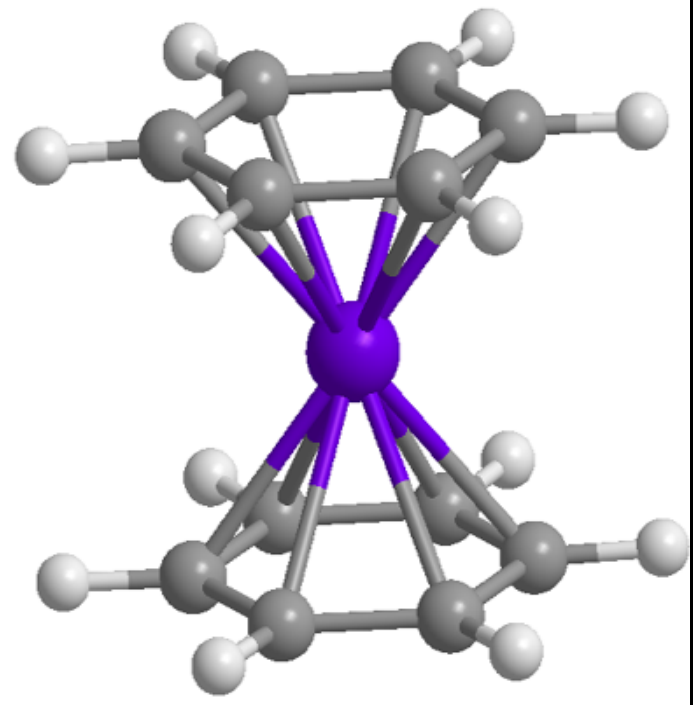
66-Co-3Q(C_2,C_{2h})
(28.5,24.5)
(28.5,24.5)

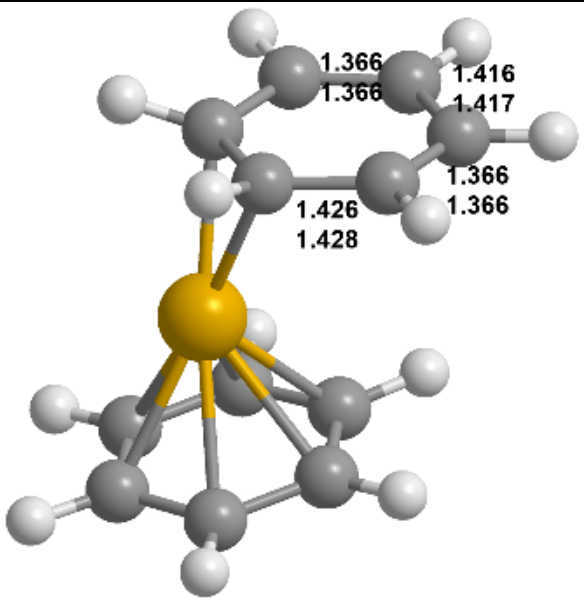

66-Rh-1D(C_s)
(0.0,0.0)
(0.0,0.0)

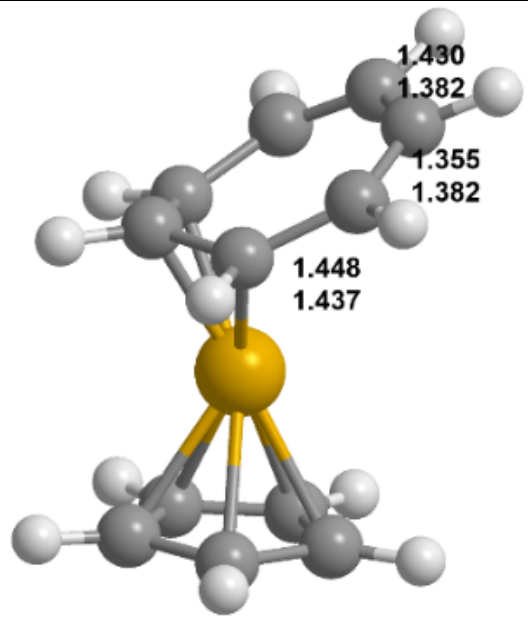

57-Rh-2D(C_s)
(4.9.7.8)
(0.0,0.0)

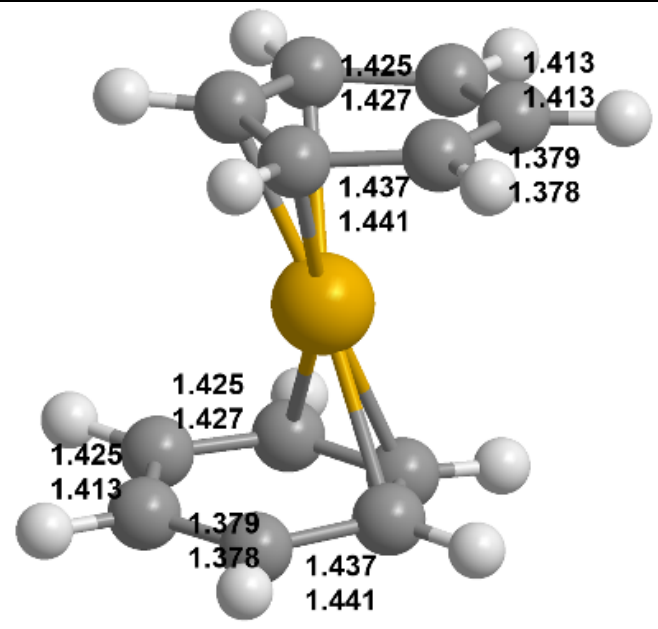

66-Rh-3Q(C_1)
(38.9,40.1)
(38.9,40.1)

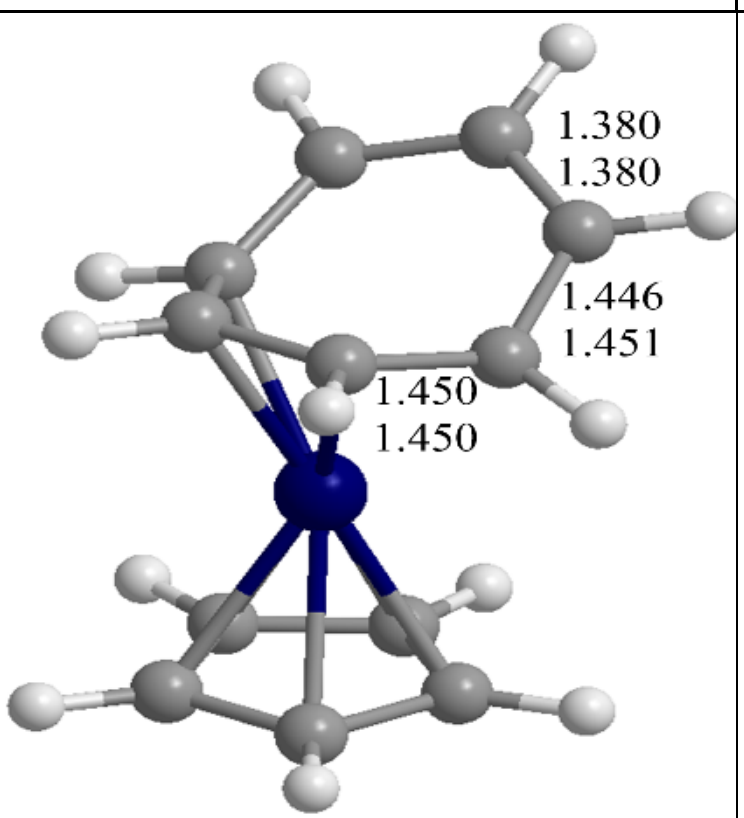

57-Ir-1D(C_s)
(0.0,0.0)
(0.0,0.0)

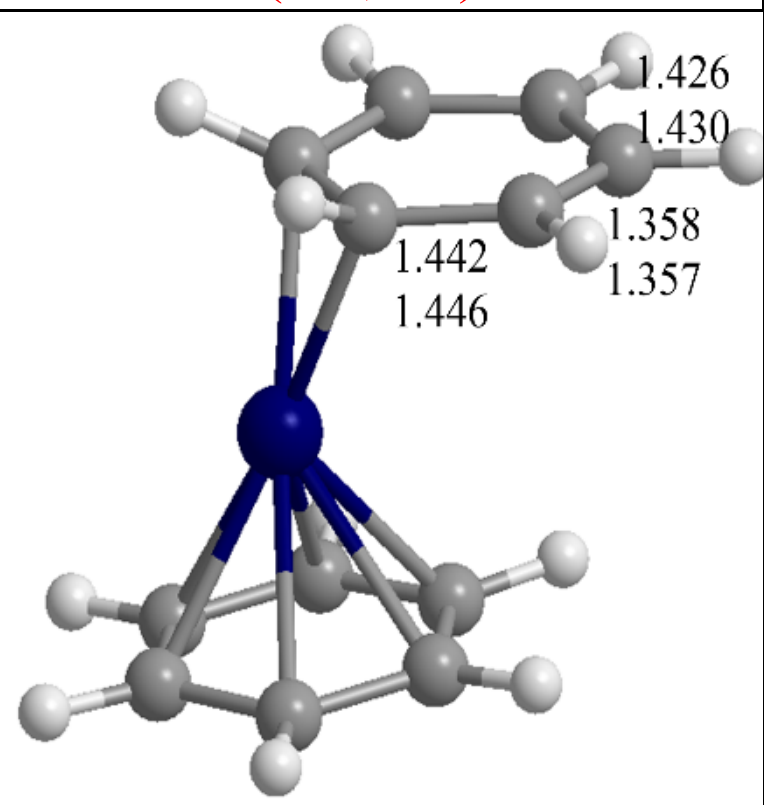

66-Ir-2D(C_s)
(8.3,5.6)
(0.0,0.0)

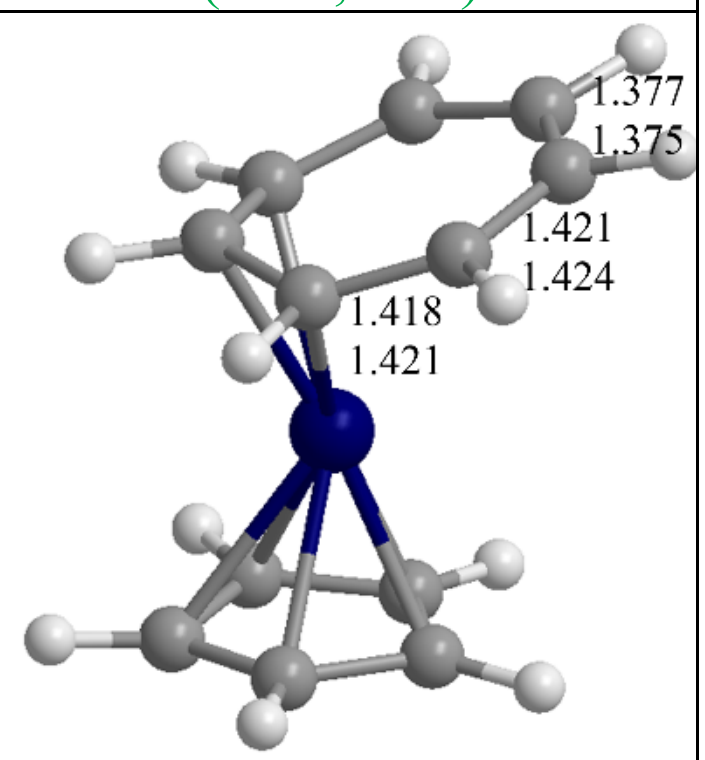

57-Ir-3Q(C_s)
(47.3,47.7)
(47.3,47.7)

Figure S7. Optimized (C_nH_n)Ni(C_mH_m) (n=6,5,4;m=6,7,8) structures

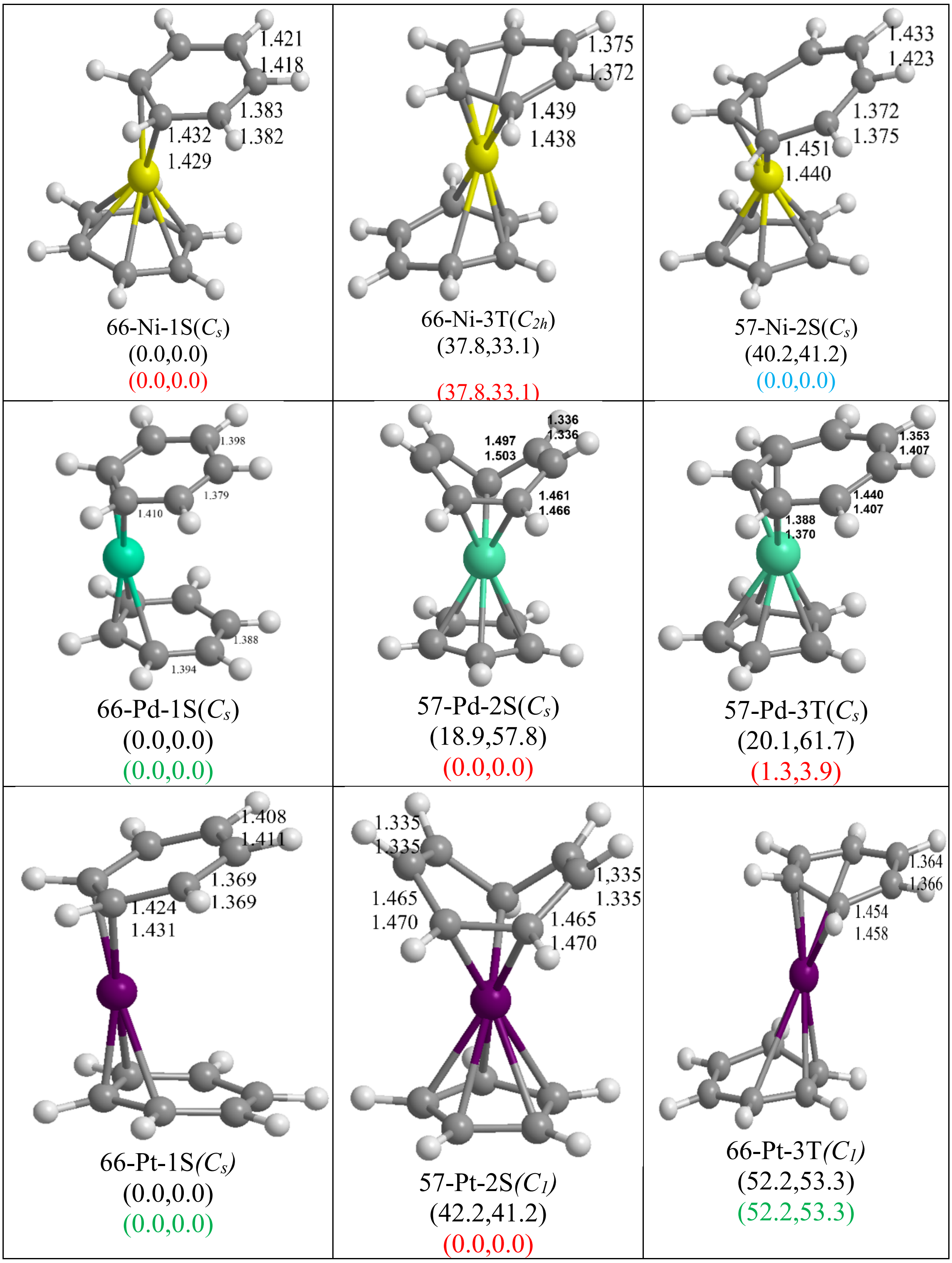

Table S1. Harmonic vibrational frequencies (in cm^{-1}) and infrared intensities (in parentheses, in km/mol) for the $C_5H_5TiC_7H_7$ structure **Ti1-57S**

B3PW91			M06-L		
7(0)	806(2)	1368(0)	19(0)	782(2)	1365(0)
98(0)	807(0)	1404(0)	91(0)	794(81)	1407(0)
99(0)	856(0)	1404(0)	94(0)	845(0)	1408(0)
249(0)	856(0)	1441(1)	254(0)	845(0)	1440(3)
291(0)	868(26)	1441(1)	301(0)	866(29)	1441(3)
291(0)	897(0)	1467(1)	301(0)	878(0)	1469(0)
337(0)	898(0)	1467(0)	337(0)	878(0)	1469(0)
337(0)	903(0)	1502(0)	337(0)	883(0)	1495(0)
422(4)	904(0)	1502(0)	421(3)	883(0)	1495(0)
422(4)	927(0)	1511(0)	422(3)	924(0)	1522(0)
453(0)	927(0)	1511(0)	448(0)	924(0)	1523(0)
497(0)	969(20)	3179(0)	495(0)	969(21)	3186(0)
497(0)	970(20)	3179(0)	495(0)	970(20)	3186(0)
557(0)	1031(15)	3188(0)	547(0)	1034(12)	3195(0)
558(0)	1031(15)	3188(0)	548(0)	1034(12)	3195(0)
626(0)	1079(0)	3200(9)	623(0)	1082(0)	3206(12)
626(0)	1080(0)	3201(9)	625(0)	1082(0)	3207(12)
761(26)	1148(6)	3208(0)	740(10)	1150(10)	3215(0)
762(9)	1178(0)	3233(0)	740(10)	1179(0)	3230(0)
762(132)	1178(0)	3233(0)	751(116)	1179(0)	3230(0)
800(91)	1249(0)	3248(1)	777(0)	1248(0)	3247(2)
803(3)	1249(0)	3248(1)	777(0)	1249(0)	3247(2)
803(2)	1282(0)	3260(0)	781(2)	1276(0)	3259(0)

Table S2. Harmonic vibrational frequencies (in cm^{-1}) and infrared intensities (in parentheses, in km/mol) for the $C_6H_6TiC_6H_6$ structure **Ti2-66S**

B3PW91			M06-L		
45(0)	776(2)	1336(0)	42(0)	752(0)	1333(0)
66(0)	776(2)	1395(0)	54(0)	752(0)	1406(0)
66(0)	826(0)	1406(0)	55(0)	789(0)	1421(0)
255(0)	826(0)	1445(0)	248(0)	789(0)	1442(0)
304(0)	846(0)	1445(0)	297(0)	816(0)	1442(0)
304(0)	846(0)	1447(0)	297(0)	816(0)	1446(0)
398(0)	894(0)	1447(0)	362(119)	867(0)	1446(0)
398(0)	897(0)	1449(0)	388(0)	870(0)	1447(0)
405(148)	973(57)	1449(0)	388(0)	971(70)	1447(0)
453(0)	973(0)	1522(0)	446(11)	973(0)	1523(0)
453(0)	1006(31)	1522(0)	446(11)	1006(0)	1523(0)
460(15)	1006(31)	3206(0)	451(0)	1006(0)	3209(0)
460(15)	1006(0)	3208(0)	451(0)	1006(29)	3210(0)
610(0)	1006(0)	3212(0)	597(0)	1006(29)	3215(0)
610(0)	1032(0)	3212(0)	597(0)	1027(0)	3215(0)
617(0)	1049(0)	3212(0)	605(0)	1044(0)	3215(0)
617(0)	1119(0)	3212(0)	607(0)	1121(0)	3215(0)
620(0)	1119(0)	3225(0)	607(0)	1121(0)	3228(0)
636(0)	1142(0)	3225(0)	618(0)	1140(0)	3228(0)
718(107)	1146(0)	3225(10)	705(91)	1143(0)	3228(15)
734(0)	1156(0)	3225(10)	717(0)	1160(0)	3228(14)
774(0)	1156(0)	3233(0)	751(1)	1160(0)	3236(0)
774(0)	1335(0)	3233(0)	751(1)	1333(0)	3236(0)

Table S3. Harmonic vibrational frequencies (in cm^{-1}) and infrared intensities (in parentheses, in km/mol) for the $C_5H_5TiC_7H_7$ structure **Ti3-57T**

B3PW91			M06-L		
10(0)	814(2)	1283(0)	-111(0)	800(3)	1280(1)
37(0)	850(0)	1383(0)	8(0)	835(0)	1384(0)
99(0)	850(0)	1399(0)	86(0)	837(0)	1403(0)
100(0)	858(7)	1403(0)	87(0)	863(9)	1407(0)
230(2)	897(0)	1441(5)	232(2)	877(0)	1446(1)
267(0)	901(0)	1456(0)	265(1)	881(0)	1459(0)
273(0)	910(0)	1469(1)	283(0)	909(0)	1472(0)
288(0)	916(6)	1469(0)	294(0)	912(1)	1472(1)
331(1)	925(1)	1496(6)	328(0)	915(3)	1497(4)
379(5)	928(0)	1537(15)	389(5)	923(1)	1546(17)
384(4)	938(1)	1564(2)	394(4)	928(3)	1562(2)
428(4)	959(5)	3159(3)	428(5)	962(4)	3153(8)
471(0)	962(7)	3162(2)	477(0)	966(6)	3158(5)
488(0)	1031(12)	3166(1)	491(0)	1034(9)	3162(2)
537(12)	1031(13)	3178(6)	531(9)	1034(10)	3177(11)
627(0)	1077(0)	3188(19)	622(0)	1079(0)	3187(26)
627(0)	1077(0)	3191(16)	623(0)	1080(0)	3190(19)
759(126)	1129(20)	3195(3)	756(85)	1136(17)	3193(7)
787(1)	1149(2)	3221(0)	771(0)	1151(4)	3219(0)
788(6)	1162(1)	3223(0)	773(1)	1165(1)	3220(0)
794(103)	1203(1)	3238(4)	787(0)	1205(1)	3237(7)
805(2)	1257(1)	3238(4)	788(83)	1258(1)	3237(7)
805(5)	1280(1)	3251(1)	796(3)	1278(0)	3249(1)

Table S4. Harmonic vibrational frequencies (in cm^{-1}) and infrared intensities (in parentheses, in km/mol) for the $C_5H_5ZrC_7H_7$ structure **Zr1-57S**

B3PW91			M06-L		
2(0)	795(0)	1360(0)	9(0)	771(1)	1358(0)
56(1)	796(54)	1399(0)	52(1)	788(90)	1401(0)
56(1)	853(19)	1399(0)	52(1)	846(0)	1402(0)
250(1)	856(0)	1432(1)	241(2)	846(0)	1432(4)
275(0)	856(0)	1432(1)	270(0)	853(22)	1433(4)
276(0)	883(0)	1466(0)	272(0)	866(0)	1467(0)
344(0)	883(0)	1466(0)	342(0)	867(0)	1468(0)
344(0)	898(0)	1486(0)	342(0)	876(0)	1480(0)
366(2)	900(0)	1486(0)	352(7)	878(0)	1481(0)
367(2)	926(0)	1509(0)	361(1)	923(0)	1510(0)
370(4)	926(0)	1509(0)	362(1)	924(0)	1511(0)
503(0)	956(27)	3178(0)	502(0)	956(27)	3188(0)
503(0)	956(27)	3178(0)	502(0)	957(27)	3188(0)
564(0)	1029(17)	3187(0)	552(0)	1031(14)	3196(0)
564(0)	1030(17)	3187(0)	553(0)	1031(13)	3196(0)
629(0)	1079(0)	3199(8)	620(0)	1081(0)	3208(9)
630(0)	1079(0)	3200(8)	624(0)	1081(0)	3209(9)
742(3)	1145(3)	3207(0)	722(3)	1145(5)	3216(0)
743(3)	1164(0)	3225(0)	722(3)	1165(0)	3225(0)
749(181)	1164(0)	3225(0)	741(153)	1166(0)	3225(0)
786(0)	1241(0)	3240(0)	757(0)	1241(0)	3241(1)
786(0)	1241(0)	3240(0)	758(0)	1241(0)	3242(1)
794(58)	1281(0)	3252(0)	771(0)	1275(0)	3253(0)

Table S5. Harmonic vibrational frequencies (in cm^{-1}) and infrared intensities (in parentheses, in km/mol) for the $C_5H_5ZrC_7H_7$ structure **Zr2-57T**

B3PW91			M06-L		
-8(0)	802(3)	1283(0)	-2(0)	792(1)	1284(2)
40(0)	842(0)	1381(0)	61(0)	830(0)	1382(0)
64(0)	845(0)	1399(0)	71(0)	834(0)	1402(0)
93(0)	854(4)	1401(0)	138(0)	855(5)	1404(0)
239(2)	894(0)	1440(5)	233(1)	876(0)	1444(1)
262(1)	895(0)	1453(0)	259(1)	877(0)	1456(0)
275(0)	905(0)	1470(2)	273(0)	903(0)	1472(0)
285(0)	911(6)	1470(2)	287(0)	907(2)	1472(1)
310(2)	917(1)	1489(4)	310(1)	910(4)	1490(3)
321(2)	922(1)	1544(15)	316(1)	921(1)	1545(16)
342(0)	933(2)	1557(2)	334(13)	928(4)	1555(2)
351(10)	954(6)	3156(4)	338(1)	956(6)	3155(8)
468(0)	956(7)	3159(2)	469(0)	959(6)	3161(5)
482(0)	1031(12)	3163(1)	478(0)	1033(9)	3165(2)
532(9)	1031(12)	3175(6)	529(7)	1033(10)	3180(8)
626(0)	1077(0)	3185(17)	624(0)	1080(0)	3190(20)
628(0)	1078(0)	3188(14)	625(0)	1080(0)	3193(15)
755(104)	1148(0)	3191(8)	751(82)	1149(1)	3196(9)
785(0)	1155(1)	3220(0)	767(0)	1159(1)	3219(0)
786(32)	1160(16)	3221(0)	768(0)	1177(14)	3221(0)
789(99)	1197(0)	3237(2)	778(1)	1201(0)	3237(4)
797(0)	1254(1)	3237(2)	781(103)	1256(1)	3238(4)
800(1)	1281(2)	3249(0)	789(0)	1278(0)	3250(0)

Table S6. Harmonic vibrational frequencies (in cm^{-1}) and infrared intensities (in parentheses, in km/mol) for the $C_6H_6ZrC_6H_6$ structure **Zr3-66S**

B3PW91			M06-L		
19(0)	767(1)	1334(0)	24(0)	744(2)	1332(0)
19(0)	767(1)	1381(0)	24(0)	744(2)	1391(0)
30(0)	816(0)	1402(0)	25(0)	790(0)	1416(0)
259(0)	816(0)	1422(0)	268(0)	790(0)	1423(0)
300(0)	829(0)	1422(0)	310(0)	800(0)	1423(0)
300(0)	829(0)	1441(0)	310(0)	800(0)	1443(1)
309(104)	886(0)	1441(0)	312(87)	865(0)	1443(1)
352(0)	886(0)	1443(0)	350(0)	865(0)	1444(0)
352(0)	959(88)	1443(0)	350(0)	963(97)	1444(0)
385(8)	962(0)	1518(0)	392(6)	966(0)	1519(0)
385(8)	997(37)	1518(0)	392(6)	1000(34)	1519(0)
457(0)	997(37)	3203(0)	461(0)	1000(34)	3207(0)
457(0)	998(0)	3204(0)	461(0)	1000(0)	3208(0)
599(0)	998(0)	3208(0)	593(0)	1000(0)	3213(0)
599(0)	1030(0)	3208(0)	593(0)	1030(0)	3213(0)
612(0)	1045(0)	3209(0)	608(0)	1044(0)	3213(0)
612(0)	1100(0)	3209(0)	608(0)	1105(0)	3213(0)
621(0)	1100(0)	3221(0)	613(0)	1105(0)	3226(0)
633(0)	1143(0)	3221(0)	621(0)	1141(0)	3226(0)
703(99)	1145(0)	3222(7)	692(76)	1142(0)	3226(11)
721(0)	1154(0)	3222(7)	710(0)	1158(0)	3226(10)
764(0)	1154(0)	3230(0)	742(0)	1158(0)	3234(0)
764(0)	1334(0)	3230(0)	742(0)	1332(0)	3234(0)

Table S7. Harmonic vibrational frequencies (in cm^{-1}) and infrared intensities (in parentheses, in km/mol) for the $C_5H_5HfC_7H_7$ structure **Hf1-57S**

B3PW91			M06-L		
3(0)	794(0)	1356(0)	5(0)	770(1)	1354(0)
60(1)	797(91)	1397(0)	57(1)	790(84)	1399(0)
60(1)	851(14)	1397(0)	57(1)	847(0)	1400(0)
255(1)	858(0)	1427(1)	256(1)	847(0)	1427(5)
278(0)	858(0)	1427(1)	284(0)	849(17)	1427(5)
279(0)	876(0)	1463(0)	286(0)	858(0)	1465(0)
315(3)	876(0)	1463(0)	307(5)	859(0)	1465(0)
341(2)	896(0)	1477(0)	336(1)	874(0)	1470(0)
341(2)	898(0)	1477(0)	336(1)	876(0)	1471(0)
344(0)	927(0)	1509(0)	342(0)	924(0)	1511(0)
344(0)	927(0)	1509(0)	342(0)	924(0)	1512(0)
503(0)	951(29)	3182(0)	501(0)	951(29)	3192(0)
503(0)	952(29)	3182(0)	501(0)	952(29)	3192(0)
561(0)	1028(17)	3190(0)	547(0)	1030(13)	3200(0)
562(0)	1028(17)	3190(0)	547(0)	1030(13)	3200(0)
631(0)	1078(0)	3202(7)	623(0)	1081(0)	3211(9)
631(0)	1078(0)	3203(7)	627(0)	1081(0)	3212(9)
736(3)	1142(3)	3210(0)	715(4)	1144(5)	3219(0)
736(3)	1158(0)	3227(0)	716(3)	1159(0)	3226(0)
750(201)	1158(0)	3227(0)	743(140)	1160(0)	3226(0)
773(0)	1238(0)	3242(0)	744(29)	1237(0)	3243(1)
773(0)	1238(0)	3242(0)	745(0)	1238(0)	3243(1)
793(10)	1280(0)	3254(0)	769(0)	1274(0)	3255(1)

Table S8. Harmonic vibrational frequencies (in cm^{-1}) and infrared intensities (in parentheses, in km/mol) for the $C_6H_6HfC_6H_6$ structure **Hf2-66S**

B3PW91			M06-L		
23(0)	756(1)	1330(0)	24(0)	729(1)	1328(0)
23(0)	756(1)	1379(0)	24(0)	729(1)	1388(0)
33(0)	802(0)	1393(0)	31(0)	769(0)	1406(0)
266(68)	802(0)	1408(0)	260(57)	769(0)	1408(0)
268(0)	814(0)	1408(0)	275(0)	776(0)	1408(0)
309(0)	814(0)	1435(0)	314(0)	776(0)	1436(1)
309(0)	875(0)	1435(0)	314(0)	850(0)	1436(1)
342(0)	875(0)	1437(0)	332(0)	852(0)	1437(0)
342(0)	953(93)	1437(0)	332(0)	955(102)	1437(0)
354(7)	958(0)	1511(0)	358(5)	960(0)	1511(0)
354(7)	991(40)	1511(0)	358(5)	993(36)	1511(0)
454(0)	991(40)	3206(0)	457(0)	993(36)	3210(0)
454(0)	992(0)	3207(0)	457(0)	994(0)	3212(0)
602(0)	992(0)	3211(0)	594(0)	994(0)	3216(0)
602(0)	1031(0)	3211(0)	594(0)	1029(0)	3216(0)
613(0)	1046(0)	3212(0)	604(0)	1044(0)	3217(0)
613(0)	1090(0)	3212(0)	608(0)	1095(0)	3217(0)
615(0)	1090(0)	3224(0)	608(0)	1095(0)	3229(0)
628(0)	1140(0)	3224(0)	613(0)	1137(0)	3229(0)
701(108)	1142(0)	3224(5)	686(86)	1138(0)	3229(10)
722(0)	1152(0)	3224(5)	706(0)	1155(0)	3229(9)
751(0)	1152(0)	3232(0)	724(0)	1155(0)	3237(0)
751(0)	1330(0)	3233(0)	724(0)	1328(0)	3237(0)

Table S9. Harmonic vibrational frequencies (in cm^{-1}) and infrared intensities (in parentheses, in km/mol) for the $C_5H_5HfC_7H_7$ structure **Hf3-57T**

B3PW91			M06-L		
-9(0)	800(4)	1283(0)	4(0)	787(102)	1282(2)
32(0)	843(0)	1379(0)	55(0)	830(0)	1380(0)
51(0)	847(0)	1399(0)	60(0)	833(0)	1402(0)
93(0)	854(3)	1402(0)	141(0)	856(4)	1404(0)
241(1)	894(0)	1439(4)	238(0)	875(0)	1443(1)
245(1)	894(0)	1451(0)	245(1)	875(0)	1454(0)
268(0)	905(0)	1469(3)	267(1)	901(1)	1471(1)
283(0)	912(4)	1469(2)	284(0)	902(1)	1471(2)
286(0)	914(2)	1486(3)	290(9)	907(4)	1487(3)
288(0)	918(1)	1550(15)	293(0)	917(1)	1552(2)
298(8)	926(3)	1554(2)	295(1)	924(5)	1554(16)
332(0)	952(7)	3159(4)	331(0)	955(6)	3156(8)
457(0)	955(8)	3163(2)	460(0)	959(7)	3163(5)
474(0)	1031(11)	3166(1)	472(0)	1033(9)	3166(2)
519(8)	1031(11)	3179(5)	515(6)	1033(9)	3183(8)
628(0)	1079(0)	3190(13)	624(0)	1081(0)	3193(18)
629(0)	1079(0)	3192(11)	625(0)	1081(0)	3197(13)
755(108)	1149(0)	3196(6)	751(84)	1149(1)	3199(8)
780(0)	1153(1)	3223(0)	763(1)	1157(1)	3221(0)
785(2)	1164(15)	3224(0)	769(0)	1183(13)	3222(0)
791(2)	1197(0)	3239(1)	773(0)	1201(0)	3239(3)
795(126)	1254(1)	3240(1)	786(1)	1255(1)	3239(3)
800(2)	1279(2)	3251(0)	787(2)	1277(0)	3251(0)

Table S10. Harmonic vibrational frequencies (in cm^{-1}) and infrared intensities (in parentheses, in km/mol) for the $C_5H_5VC_7H_7$ structure **V1-57D**

B3PW91			M06-L		
9(0)	851(0)	1380(0)	11(0)	833(0)	1379(0)
135(0)	851(0)	1405(0)	125(0)	833(0)	1407(0)
136(0)	858(0)	1405(0)	128(0)	838(0)	1408(0)
252(1)	858(0)	1457(0)	253(1)	838(0)	1457(2)
308(0)	876(14)	1457(0)	316(0)	875(15)	1457(2)
309(0)	904(0)	1466(1)	316(0)	883(0)	1467(0)
328(0)	904(0)	1466(1)	326(0)	884(0)	1467(0)
329(0)	914(0)	1527(0)	327(0)	904(0)	1522(0)
440(14)	914(0)	1527(0)	436(3)	904(0)	1523(0)
440(15)	938(0)	1529(0)	441(13)	925(0)	1543(0)
448(5)	938(0)	1529(0)	442(14)	925(0)	1544(0)
499(0)	982(11)	3167(0)	500(0)	983(11)	3166(0)
499(0)	983(11)	3167(0)	500(0)	984(10)	3166(0)
554(0)	1030(12)	3177(0)	544(0)	1033(9)	3175(0)
554(0)	1030(12)	3177(0)	545(0)	1033(9)	3175(0)
622(0)	1078(0)	3189(21)	620(0)	1079(0)	3186(33)
622(0)	1078(0)	3190(21)	620(0)	1079(0)	3187(32)
800(135)	1147(7)	3197(2)	785(0)	1148(10)	3194(2)
802(1)	1190(0)	3232(0)	785(0)	1192(0)	3227(0)
802(2)	1190(0)	3232(0)	794(3)	1192(0)	3228(0)
803(49)	1259(0)	3248(3)	797(132)	1258(0)	3244(5)
824(12)	1259(0)	3248(3)	809(12)	1259(0)	3245(5)
824(12)	1282(0)	3260(0)	809(12)	1276(0)	3256(0)

Table S11. Harmonic vibrational frequencies (in cm^{-1}) and infrared intensities (in parentheses, in km/mol) for the $C_6H_6VC_6H_6$ structure **V2-66D**

B3PW91			M06-L		
48(0)	824(5)	1342(0)	51(0)	807(4)	1339(0)
119(0)	824(5)	1411(0)	109(0)	807(4)	1422(0)
119(0)	863(0)	1418(0)	109(0)	833(0)	1433(0)
261(0)	863(0)	1454(2)	253(0)	833(0)	1454(0)
321(0)	868(0)	1454(2)	319(0)	837(0)	1454(0)
321(0)	868(0)	1456(0)	319(0)	837(0)	1456(0)
406(0)	917(0)	1456(0)	395(0)	889(0)	1456(0)
406(0)	918(0)	1478(0)	395(0)	894(0)	1471(0)
429(163)	984(38)	1478(0)	398(138)	982(46)	1471(0)
454(0)	984(0)	1535(0)	451(0)	983(0)	1537(0)
454(0)	1014(0)	1535(0)	451(0)	1015(0)	1537(0)
481(27)	1014(0)	3196(0)	475(24)	1015(0)	3193(0)
481(27)	1015(19)	3198(0)	475(24)	1017(15)	3195(0)
624(0)	1015(19)	3202(0)	615(0)	1017(15)	3200(0)
624(0)	1025(0)	3202(0)	615(0)	1019(0)	3200(0)
626(0)	1040(0)	3204(0)	617(0)	1033(0)	3201(0)
626(0)	1132(0)	3204(0)	619(0)	1134(0)	3201(0)
632(0)	1132(0)	3215(0)	619(0)	1134(0)	3212(0)
643(0)	1149(0)	3215(0)	625(0)	1147(0)	3212(0)
759(84)	1154(0)	3216(27)	751(66)	1151(0)	3212(43)
766(0)	1158(0)	3216(27)	755(0)	1161(0)	3212(41)
810(0)	1158(0)	3223(3)	794(0)	1161(0)	3219(4)
810(0)	1341(0)	3224(0)	794(0)	1339(0)	3219(0)

Table S12. Harmonic vibrational frequencies (in cm^{-1}) and infrared intensities (in parentheses, in km/mol) for the $C_5H_5VC_7H_7$ structure **V3-57Q**

B3PW91			M06-L		
8(0)	823(7)	1355(12)	-22(0)	815(6)	1358(10)
75(0)	852(0)	1391(24)	71(0)	838(0)	1394(17)
98(0)	852(0)	1397(1)	86(0)	839(0)	1399(1)
166(1)	885(6)	1400(0)	146(5)	872(4)	1404(0)
182(4)	885(1)	1429(3)	157(1)	880(1)	1433(1)
243(2)	900(17)	1465(2)	241(2)	885(2)	1465(0)
276(3)	901(0)	1469(22)	284(3)	889(7)	1467(21)
317(2)	904(0)	1469(1)	331(1)	896(10)	1471(0)
340(5)	930(11)	1496(1)	351(2)	924(10)	1495(1)
348(1)	954(0)	1552(33)	352(4)	946(0)	1547(30)
400(1)	980(2)	1728(27)	400(1)	967(2)	1722(28)
406(4)	983(5)	3124(5)	409(5)	978(11)	3127(10)
435(0)	991(5)	3128(8)	435(0)	985(1)	3129(10)
566(34)	1028(14)	3130(3)	561(27)	1030(11)	3133(0)
574(1)	1031(14)	3145(39)	569(2)	1034(11)	3151(29)
622(0)	1076(0)	3155(34)	615(0)	1078(0)	3158(39)
624(0)	1078(0)	3158(10)	620(0)	1081(0)	3162(20)
740(24)	1131(1)	3191(7)	738(22)	1134(1)	3195(10)
764(0)	1147(4)	3224(0)	756(0)	1148(8)	3224(0)
793(107)	1181(10)	3227(0)	779(8)	1184(10)	3225(0)
797(6)	1221(3)	3241(3)	789(1)	1226(3)	3241(5)
805(24)	1227(6)	3242(3)	789(53)	1231(5)	3242(5)
805(1)	1282(0)	3254(0)	798(26)	1277(0)	3253(0)

Table S13. Harmonic vibrational frequencies (in cm^{-1}) and infrared intensities (in parentheses, in km/mol) for the $C_5H_5NbC_7H_7$ structure **Nb1-57D**

B3PW91			M06-L		
26(0)	752(0)	1338(0)	16(0)	818(0)	1374(0)
96(0)	782(0)	1338(0)	93(0)	818(0)	1402(0)
98(1)	797(42)	1372(30)	93(0)	835(0)	1403(0)
160(0)	812(0)	1374(0)	261(2)	835(0)	1450(2)
226(1)	828(1)	1437(0)	317(0)	870(8)	1451(2)
238(4)	918(0)	1438(0)	318(0)	881(0)	1465(0)
238(0)	919(0)	1441(0)	323(0)	882(0)	1466(0)
248(0)	925(0)	1493(0)	323(0)	897(0)	1510(0)
278(4)	941(0)	1495(4)	366(0)	898(0)	1510(0)
288(0)	952(86)	1547(34)	385(9)	926(0)	1536(0)
290(0)	954(0)	1584(0)	386(9)	926(0)	1536(0)
402(0)	960(51)	3180(0)	510(0)	975(13)	3163(0)
443(0)	965(0)	3183(0)	510(0)	975(12)	3164(0)
551(8)	1008(2)	3184(4)	550(0)	1031(10)	3172(0)
573(0)	1016(0)	3185(0)	551(0)	1031(10)	3172(0)
583(0)	1026(2)	3198(6)	619(0)	1079(0)	3182(37)
591(0)	1027(0)	3198(0)	621(0)	1079(0)	3182(36)
607(0)	1116(0)	3200(36)	779(0)	1143(7)	3188(6)
609(0)	1149(0)	3200(0)	780(1)	1180(0)	3225(0)
672(0)	1150(0)	3231(16)	788(69)	1180(0)	3225(0)
692(0)	1169(3)	3231(0)	801(86)	1250(0)	3242(4)
701(70)	1178(0)	3233(0)	809(3)	1251(0)	3242(4)
706(4)	1293(0)	3233(0)	811(3)	1276(0)	3253(0)

Table S14. Harmonic vibrational frequencies (in cm^{-1}) and infrared intensities (in parentheses, in km/mol) for the $C_6H_6NbC_6H_6$ structure **Nb2-66D**

B3PW91			M06-L		
33(0)	821(0)	1341(0)	44(0)	806(0)	1339(0)
81(1)	821(0)	1396(0)	74(1)	807(0)	1407(0)
81(1)	833(0)	1416(0)	77(1)	808(0)	1429(0)
270(0)	833(0)	1450(2)	259(0)	809(0)	1451(0)
326(0)	867(0)	1450(2)	307(99)	842(0)	1453(0)
326(0)	867(0)	1452(0)	318(0)	842(0)	1453(0)
341(121)	909(0)	1452(0)	320(0)	890(0)	1455(0)
362(0)	910(0)	1456(0)	350(0)	891(0)	1455(0)
362(0)	974(50)	1456(0)	354(0)	974(58)	1455(0)
414(19)	974(0)	1532(0)	404(16)	975(0)	1531(0)
414(19)	1007(0)	1532(0)	406(16)	1009(0)	1532(0)
460(0)	1007(0)	3192(0)	457(0)	1010(17)	3192(0)
460(0)	1008(20)	3194(0)	459(0)	1011(0)	3193(0)
608(0)	1008(20)	3198(0)	602(0)	1012(16)	3198(0)
608(0)	1020(0)	3198(0)	602(0)	1016(0)	3198(0)
616(0)	1032(0)	3199(0)	608(0)	1028(0)	3198(0)
616(0)	1120(0)	3199(0)	609(0)	1124(0)	3198(0)
631(0)	1120(0)	3211(0)	620(0)	1124(0)	3210(35)
635(0)	1149(0)	3211(0)	620(0)	1147(0)	3210(0)
750(79)	1153(0)	3211(27)	741(60)	1151(0)	3210(38)
758(0)	1156(0)	3211(27)	747(0)	1158(0)	3210(0)
809(0)	1156(0)	3217(7)	793(0)	1159(0)	3215(6)
809(0)	1340(0)	3218(0)	794(0)	1339(0)	3216(0)

Table S15. Harmonic vibrational frequencies (in cm^{-1}) and infrared intensities (in parentheses, in km/mol) for the $C_6H_6NbC_6H_6$ structure **Nb3-66Q**

B3PW91			M06-L		
24(0)	780(0)	1338(0)	26(0)	752(0)	1338(0)
85(0)	794(0)	1339(0)	96(0)	782(0)	1338(0)
91(0)	810(44)	1362(43)	98(1)	797(42)	1372(30)
165(0)	819(0)	1365(0)	160(0)	812(0)	1374(0)
210(1)	835(1)	1433(0)	226(1)	828(1)	1437(0)
242(0)	930(0)	1433(1)	238(4)	918(0)	1438(0)
254(0)	933(0)	1440(0)	238(0)	919(0)	1441(0)
261(5)	938(4)	1492(0)	248(0)	925(0)	1493(0)
274(2)	950(0)	1493(2)	278(4)	941(0)	1495(4)
284(0)	950(81)	1551(42)	288(0)	952(86)	1547(34)
297(0)	954(0)	1584(0)	290(0)	954(0)	1584(0)
401(0)	955(56)	3181(0)	402(0)	960(51)	3180(0)
448(0)	964(0)	3183(0)	443(0)	965(0)	3183(0)
551(11)	1007(3)	3184(3)	551(8)	1008(2)	3184(4)
578(0)	1017(0)	3185(0)	573(0)	1016(0)	3185(0)
586(0)	1023(2)	3197(7)	583(0)	1026(2)	3198(6)
597(0)	1023(0)	3198(0)	591(0)	1027(0)	3198(0)
608(0)	1110(0)	3200(0)	607(0)	1116(0)	3200(36)
613(0)	1150(0)	3200(28)	609(0)	1149(0)	3200(0)
685(0)	1151(0)	3226(10)	672(0)	1150(0)	3231(16)
708(104)	1165(4)	3226(0)	692(0)	1169(3)	3231(0)
710(0)	1174(0)	3228(0)	701(70)	1178(0)	3233(0)
722(2)	1296(0)	3228(0)	706(4)	1293(0)	3233(0)

Table S16. Harmonic vibrational frequencies (in cm^{-1}) and infrared intensities (in parentheses, in km/mol) for the $C_5H_5TaC_7H_7$ structure **Ta1-57D**

B3PW91			M06-L		
12(0)	825(0)	1372(0)	16(0)	807(3)	1370(0)
88(0)	825(0)	1398(0)	84(0)	809(3)	1400(0)
89(0)	846(0)	1398(0)	85(0)	831(0)	1400(0)
273(1)	846(0)	1445(0)	270(1)	832(0)	1446(2)
318(0)	869(3)	1445(0)	310(0)	869(5)	1447(2)
318(0)	898(0)	1461(2)	311(0)	878(0)	1462(1)
319(1)	898(0)	1461(2)	315(0)	878(0)	1462(1)
319(1)	904(0)	1507(0)	326(0)	892(0)	1502(0)
327(0)	904(0)	1507(0)	326(0)	892(0)	1503(0)
349(7)	927(0)	1534(0)	348(6)	922(0)	1540(0)
349(7)	928(0)	1534(0)	348(6)	922(0)	1540(0)
503(0)	969(15)	3161(0)	504(0)	971(14)	3163(0)
503(0)	969(14)	3161(0)	504(0)	972(14)	3164(0)
552(0)	1027(12)	3170(0)	538(0)	1029(9)	3171(0)
552(0)	1027(12)	3170(0)	539(0)	1029(9)	3172(0)
624(0)	1077(0)	3180(30)	620(0)	1078(0)	3180(41)
624(0)	1077(0)	3181(29)	622(0)	1078(0)	3180(40)
799(0)	1140(5)	3187(6)	778(0)	1140(8)	3186(8)
799(6)	1174(0)	3229(0)	778(0)	1176(0)	3225(0)
800(134)	1174(0)	3229(0)	792(55)	1176(0)	3225(0)
803(61)	1248(0)	3244(1)	801(53)	1247(0)	3242(3)
820(4)	1248(0)	3244(1)	802(0)	1248(0)	3242(3)
820(4)	1281(0)	3255(1)	803(47)	1274(0)	3252(1)

Table S17. Harmonic vibrational frequencies (in cm^{-1}) and infrared intensities (in parentheses, in km/mol) for the $C_6H_6TaC_6H_6$ structure **Ta2-66D**

B3PW91			M06-L		
28(0)	780(0)	1334(0)	46(0)	786(0)	1335(0)
86(0)	785(0)	1335(0)	68(1)	787(0)	1408(0)
89(1)	799(53)	1369(0)	70(1)	799(0)	1426(0)
222(0)	811(0)	1369(32)	274(0)	800(0)	1439(0)
224(0)	826(1)	1433(0)	282(72)	826(0)	1440(0)
243(0)	901(0)	1434(0)	313(0)	827(0)	1446(0)
252(5)	915(0)	1438(0)	316(0)	882(0)	1447(0)
262(0)	921(0)	1491(0)	332(0)	883(0)	1448(0)
268(0)	935(0)	1494(7)	333(0)	972(62)	1450(0)
294(0)	950(0)	1540(15)	371(12)	974(0)	1527(0)
307(0)	952(71)	1581(0)	372(12)	1005(0)	1527(0)
409(0)	956(51)	3180(0)	453(0)	1006(18)	3192(0)
434(0)	964(0)	3183(0)	455(0)	1006(0)	3193(0)
547(5)	1009(2)	3184(4)	602(0)	1007(18)	3197(0)
575(0)	1015(0)	3185(0)	602(0)	1015(0)	3197(0)
577(0)	1022(1)	3197(5)	609(0)	1028(0)	3198(0)
581(0)	1022(0)	3197(0)	610(0)	1117(0)	3198(0)
603(0)	1115(0)	3199(36)	612(0)	1117(0)	3208(37)
607(0)	1147(0)	3199(0)	614(0)	1145(0)	3209(0)
676(0)	1148(0)	3231(17)	745(65)	1148(0)	3209(40)
703(0)	1166(1)	3232(0)	750(0)	1156(0)	3209(0)
707(58)	1177(0)	3233(0)	775(0)	1156(0)	3213(7)
710(8)	1291(0)	3233(0)	776(0)	1335(0)	3214(0)

Table S18. Harmonic vibrational frequencies (in cm^{-1}) and infrared intensities (in parentheses, in km/mol) for the $C_6H_6TaC_6H_6$ structure **Ta3-66Q**

B3PW91			M06-L		
30(0)	792(0)	1335(0)	28(0)	780(0)	1334(0)
77(0)	808(0)	1336(0)	86(0)	785(0)	1335(0)
82(1)	811(53)	1360(0)	89(1)	799(53)	1369(0)
213(0)	819(0)	1361(46)	222(0)	811(0)	1369(32)
231(0)	834(2)	1429(0)	224(0)	826(1)	1433(0)
234(1)	920(0)	1430(1)	243(0)	901(0)	1434(0)
241(3)	926(0)	1438(0)	252(5)	915(0)	1438(0)
264(0)	933(3)	1490(0)	262(0)	921(0)	1491(0)
280(0)	946(0)	1492(4)	268(0)	935(0)	1494(7)
287(0)	950(0)	1545(18)	294(0)	950(0)	1540(15)
307(0)	950(64)	1581(0)	307(0)	952(71)	1581(0)
405(0)	950(57)	3182(0)	409(0)	956(51)	3180(0)
440(0)	962(0)	3185(0)	434(0)	964(0)	3183(0)
547(6)	1010(3)	3185(3)	547(5)	1009(2)	3184(4)
577(0)	1017(0)	3186(0)	575(0)	1015(0)	3185(0)
578(1)	1018(2)	3198(5)	577(0)	1022(1)	3197(5)
585(0)	1019(0)	3199(0)	581(0)	1022(0)	3197(0)
606(0)	1110(0)	3201(27)	603(0)	1115(0)	3199(36)
614(0)	1148(0)	3201(0)	607(0)	1147(0)	3199(0)
688(0)	1149(0)	3227(11)	676(0)	1148(0)	3231(17)
713(90)	1162(2)	3227(0)	703(0)	1166(1)	3232(0)
716(0)	1173(0)	3229(1)	707(58)	1177(0)	3233(0)
725(4)	1295(0)	3229(0)	710(8)	1291(0)	3233(0)

Table S19. Harmonic vibrational frequencies (in cm^{-1}) and infrared intensities (in parentheses, in km/mol) for the $C_6H_6CrC_6H_6$ structure **Cr1-66S**

B3PW91			M06-L		
52(0)	882(7)	1349(0)	54(0)	849(0)	1347(0)
153(0)	882(7)	1432(0)	144(0)	849(0)	1443(0)
153(1)	884(0)	1435(0)	144(0)	876(5)	1451(0)
269(0)	884(0)	1466(4)	270(0)	876(5)	1465(1)
336(0)	925(0)	1466(4)	351(0)	904(0)	1465(1)
336(0)	925(0)	1469(0)	351(0)	908(0)	1468(0)
401(0)	938(0)	1469(0)	387(0)	908(0)	1468(0)
401(0)	942(0)	1513(0)	387(0)	915(0)	1506(0)
458(0)	999(0)	1513(0)	454(170)	1001(0)	1506(0)
458(0)	999(19)	1552(0)	460(0)	1002(21)	1555(0)
459(188)	1016(0)	1552(0)	460(0)	1011(0)	1555(0)
498(44)	1025(0)	3175(0)	513(47)	1021(0)	3160(0)
498(44)	1025(0)	3176(0)	513(47)	1027(0)	3161(0)
625(0)	1028(8)	3181(0)	625(0)	1027(0)	3168(0)
625(0)	1028(8)	3181(0)	625(0)	1031(4)	3168(0)
637(0)	1028(0)	3183(0)	636(0)	1031(4)	3169(0)
637(0)	1145(0)	3183(0)	638(0)	1146(0)	3169(0)
645(0)	1145(0)	3194(0)	639(0)	1146(0)	3177(124)
650(0)	1156(0)	3194(0)	639(0)	1154(0)	3177(121)
808(0)	1160(0)	3194(73)	804(0)	1158(0)	3178(0)
808(50)	1160(0)	3194(73)	812(27)	1161(0)	3178(0)
856(0)	1161(0)	3200(20)	843(0)	1161(0)	3180(37)
856(0)	1349(0)	3202(0)	843(0)	1346(0)	3185(0)

Table S20. Harmonic vibrational frequencies (in cm^{-1}) and infrared intensities (in parentheses, in km/mol) for the $C_5H_5CrC_7H_7$ structure **Cr2-57S**

B3PW91			M06-L		
6(0)	876(12)	1393(0)	7(0)	873(10)	1392(0)
161(0)	877(12)	1409(0)	150(0)	874(10)	1411(0)
161(0)	890(4)	1409(0)	154(0)	886(0)	1411(0)
265(2)	905(0)	1466(1)	265(1)	888(0)	1465(0)
322(0)	906(0)	1466(1)	318(0)	891(4)	1466(0)
323(0)	909(0)	1471(0)	319(0)	902(0)	1472(1)
325(0)	910(0)	1471(0)	338(1)	903(0)	1473(1)
325(0)	922(0)	1541(0)	338(1)	910(0)	1551(0)
463(32)	922(0)	1541(0)	465(21)	910(0)	1552(0)
463(33)	963(0)	1558(0)	475(35)	945(0)	1567(0)
468(22)	964(0)	1558(0)	475(35)	946(0)	1567(0)
497(0)	996(4)	3147(0)	501(0)	999(3)	3133(0)
497(0)	997(4)	3147(0)	502(0)	999(2)	3133(0)
555(0)	1031(8)	3156(0)	544(0)	1033(5)	3142(0)
555(0)	1031(8)	3156(0)	545(0)	1033(4)	3143(0)
617(0)	1076(0)	3167(50)	615(0)	1076(0)	3148(90)
618(0)	1076(0)	3168(50)	617(0)	1076(0)	3150(88)
816(52)	1150(7)	3174(6)	811(1)	1151(10)	3156(12)
823(1)	1203(0)	3223(0)	811(7)	1204(0)	3211(0)
823(2)	1203(0)	3224(0)	814(16)	1204(0)	3211(0)
840(0)	1266(0)	3239(10)	815(0)	1264(0)	3228(17)
840(0)	1266(0)	3240(10)	816(0)	1265(0)	3229(16)
842(70)	1284(0)	3251(3)	845(49)	1277(0)	3237(7)

Table S21. Harmonic vibrational frequencies (in cm^{-1}) and infrared intensities (in parentheses, in km/mol) for the $C_5H_5CrC_7H_7$ structure **Cr3-57T**

B3PW91			M06-L		
34(0)	832(3)	1358(0)	17(0)	824(3)	1365(0)
89(1)	844(1)	1396(0)	75(1)	828(1)	1400(0)
127(0)	851(0)	1401(3)	119(0)	834(0)	1404(0)
148(5)	853(0)	1405(7)	119(3)	839(1)	1419(3)
249(1)	902(1)	1412(7)	246(0)	883(0)	1425(2)
301(0)	904(22)	1450(1)	301(0)	890(18)	1460(1)
312(0)	906(9)	1463(1)	315(1)	893(1)	1464(2)
330(0)	916(0)	1470(2)	336(0)	912(9)	1472(1)
368(4)	936(3)	1490(18)	368(3)	913(3)	1491(10)
382(8)	938(1)	1503(0)	386(7)	925(2)	1512(0)
430(1)	959(1)	1688(10)	425(0)	956(7)	1664(5)
459(7)	974(6)	3129(2)	459(8)	957(2)	3137(1)
491(6)	999(3)	3143(10)	507(4)	999(5)	3151(9)
580(5)	1024(14)	3148(16)	569(3)	1025(10)	3156(13)
589(1)	1033(13)	3155(37)	578(1)	1036(10)	3161(19)
614(0)	1077(0)	3162(11)	607(0)	1079(0)	3168(25)
621(0)	1078(0)	3167(25)	615(0)	1080(0)	3172(29)
732(36)	1125(2)	3183(11)	725(32)	1137(1)	3184(11)
780(6)	1146(7)	3229(0)	772(4)	1147(11)	3228(0)
798(56)	1169(8)	3237(0)	784(1)	1181(4)	3232(0)
808(36)	1206(1)	3248(2)	789(39)	1219(1)	3247(3)
811(14)	1222(0)	3250(1)	800(38)	1229(0)	3247(3)
822(4)	1283(0)	3263(0)	804(3)	1277(0)	3259(0)

Table S22. Harmonic vibrational frequencies (in cm^{-1}) and infrared intensities (in parentheses, in km/mol) for the $C_6H_6MoC_6H_6$ structure **Mo1-66S**

B3PW91			M06-L		
37(0)	858(0)	1347(0)	45(0)	851(0)	1346(0)
123(2)	858(0)	1415(0)	117(1)	851(0)	1428(0)
123(2)	883(1)	1433(0)	117(1)	878(0)	1445(0)
284(0)	883(1)	1459(5)	273(0)	878(0)	1461(1)
351(0)	929(0)	1459(5)	343(0)	911(0)	1461(1)
351(0)	931(0)	1462(0)	343(0)	915(0)	1464(0)
353(0)	931(0)	1462(0)	344(117)	920(0)	1464(0)
353(0)	932(0)	1490(0)	351(0)	920(0)	1490(0)
373(136)	989(0)	1490(0)	351(0)	992(0)	1490(0)
436(38)	992(23)	1548(0)	435(35)	994(27)	1549(0)
436(38)	1012(0)	1548(0)	435(35)	1010(0)	1549(0)
467(0)	1016(0)	3172(0)	467(0)	1018(0)	3165(0)
467(0)	1016(0)	3173(0)	467(0)	1019(0)	3166(0)
603(0)	1019(7)	3178(0)	603(0)	1019(0)	3171(0)
603(0)	1019(7)	3178(0)	603(0)	1024(5)	3171(0)
637(0)	1020(0)	3179(0)	632(0)	1024(5)	3172(0)
637(0)	1134(0)	3179(0)	637(0)	1137(0)	3172(0)
638(0)	1134(0)	3189(83)	637(0)	1137(0)	3181(114)
644(0)	1155(0)	3189(83)	642(0)	1154(0)	3181(112)
801(48)	1156(0)	3190(0)	798(0)	1158(0)	3182(0)
803(0)	1156(0)	3190(0)	800(32)	1158(0)	3182(0)
844(0)	1160(0)	3194(39)	820(0)	1158(0)	3184(49)
844(0)	1347(0)	3196(0)	820(0)	1346(0)	3187(0)

Table S23. Harmonic vibrational frequencies (in cm^{-1}) and infrared intensities (in parentheses, in km/mol) for the $C_5H_5MoC_7H_7$ structure **Mo2-57S**

B3PW91			M06-L		
12(0)	884(4)	1388(0)	16(0)	880(3)	1388(0)
130(1)	884(4)	1402(0)	121(0)	881(3)	1404(0)
130(1)	889(0)	1402(0)	121(0)	885(0)	1405(0)
281(2)	898(0)	1461(2)	271(2)	885(0)	1463(1)
322(0)	899(0)	1461(2)	316(0)	891(0)	1463(1)
322(0)	904(0)	1465(0)	317(0)	897(0)	1467(1)
343(0)	904(0)	1465(0)	344(0)	897(0)	1468(1)
343(0)	923(0)	1544(0)	344(0)	913(0)	1541(0)
397(11)	924(0)	1544(0)	375(7)	913(0)	1541(0)
408(28)	960(0)	1553(0)	406(26)	950(0)	1561(0)
408(28)	962(0)	1553(0)	406(26)	951(0)	1561(0)
509(0)	987(4)	3139(0)	512(0)	991(3)	3135(0)
509(0)	988(4)	3139(0)	512(0)	991(3)	3135(0)
555(0)	1028(8)	3148(0)	547(0)	1030(5)	3144(0)
555(0)	1028(8)	3148(0)	547(0)	1031(5)	3144(0)
615(0)	1075(0)	3157(68)	612(0)	1076(0)	3150(93)
615(0)	1075(0)	3158(68)	614(0)	1076(0)	3151(92)
808(48)	1141(5)	3164(14)	800(17)	1143(8)	3156(21)
820(0)	1189(0)	3223(0)	800(0)	1193(0)	3216(0)
820(0)	1190(0)	3223(0)	800(11)	1193(0)	3216(0)
822(0)	1258(0)	3239(7)	803(0)	1257(0)	3233(12)
822(0)	1258(0)	3239(7)	804(1)	1258(0)	3233(12)
847(70)	1283(0)	3249(6)	847(54)	1277(0)	3243(9)

Table S24. Harmonic vibrational frequencies (in cm^{-1}) and infrared intensities (in parentheses, in km/mol) for the $C_5H_5MoC_7H_7$ structure **Mo3-57T**

B3PW91			M06-L		
22(0)	830(2)	1352(0)	6(0)	810(3)	1354(0)
72(1)	830(3)	1392(0)	79(1)	823(2)	1396(1)
96(0)	844(0)	1395(6)	91(0)	830(0)	1398(4)
137(5)	845(0)	1399(3)	146(3)	832(1)	1402(3)
274(1)	897(0)	1399(5)	272(1)	881(0)	1403(1)
315(0)	898(24)	1431(0)	314(0)	885(0)	1431(0)
321(0)	902(11)	1458(1)	320(1)	897(21)	1459(0)
331(2)	904(0)	1461(2)	338(1)	903(11)	1462(2)
340(4)	932(1)	1473(13)	341(3)	921(0)	1470(9)
373(7)	943(1)	1497(1)	373(6)	941(1)	1501(1)
385(2)	964(0)	1688(9)	379(2)	964(0)	1688(11)
441(4)	973(11)	3125(1)	446(4)	969(11)	3130(1)
499(3)	995(1)	3136(6)	510(2)	997(1)	3142(7)
587(5)	1022(14)	3141(22)	586(4)	1025(11)	3147(18)
589(1)	1027(14)	3151(32)	590(1)	1029(10)	3153(17)
608(0)	1075(0)	3156(26)	602(0)	1078(0)	3159(44)
618(0)	1076(0)	3162(26)	614(0)	1078(0)	3163(34)
735(37)	1113(2)	3175(13)	734(31)	1116(2)	3174(16)
783(3)	1138(9)	3230(0)	772(1)	1139(13)	3228(0)
793(54)	1162(11)	3235(0)	782(5)	1166(11)	3232(0)
801(33)	1198(0)	3247(1)	789(40)	1201(0)	3247(2)
808(1)	1213(0)	3248(1)	794(1)	1214(0)	3247(2)
812(2)	1280(0)	3260(0)	798(14)	1274(0)	3259(0)

Table S25. Harmonic vibrational frequencies (in cm^{-1}) and infrared intensities (in parentheses, in km/mol) for the $C_6H_6WC_6H_6$ structure **W1-66S**

B3PW91			M06-L		
36(0)	860(0)	1345(0)	51(0)	854(0)	1343(0)
116(3)	860(0)	1417(0)	111(2)	855(0)	1430(0)
116(3)	885(0)	1433(0)	111(2)	882(0)	1447(0)
294(0)	885(0)	1456(5)	270(0)	883(0)	1457(1)
295(0)	923(0)	1456(5)	273(0)	907(0)	1458(1)
295(0)	926(0)	1459(0)	286(0)	910(0)	1460(0)
332(104)	930(0)	1459(0)	316(91)	919(0)	1461(0)
361(0)	930(0)	1476(0)	363(0)	920(0)	1477(0)
361(0)	988(0)	1476(0)	363(0)	991(0)	1477(0)
392(26)	991(26)	1546(0)	391(26)	994(29)	1547(0)
392(26)	1011(0)	1546(0)	392(26)	1007(0)	1547(0)
467(0)	1013(0)	3174(0)	465(0)	1014(0)	3164(0)
467(0)	1013(0)	3174(0)	467(0)	1016(0)	3164(0)
602(0)	1016(9)	3179(0)	601(0)	1017(0)	3170(0)
602(0)	1016(9)	3179(0)	602(0)	1020(6)	3170(0)
628(0)	1020(0)	3180(0)	620(0)	1021(6)	3171(0)
634(0)	1129(0)	3180(0)	629(0)	1133(0)	3171(0)
634(0)	1129(0)	3189(83)	633(0)	1133(0)	3178(115)
634(0)	1154(0)	3189(83)	634(0)	1152(0)	3179(121)
808(0)	1155(0)	3190(0)	788(0)	1156(0)	3179(0)
808(57)	1155(0)	3190(0)	789(0)	1156(0)	3180(57)
815(0)	1159(0)	3193(43)	804(0)	1157(0)	3180(0)
815(0)	1345(0)	3195(0)	809(38)	1343(0)	3184(0)

Table S26. Harmonic vibrational frequencies (in cm^{-1}) and infrared intensities (in parentheses, in km/mol) for the $C_5H_5WC_7H_7$ structure **W2-57S**

B3PW91			M06-L		
13(0)	886(4)	1386(0)	17(0)	882(0)	1385(0)
122(1)	887(4)	1400(0)	115(1)	883(0)	1402(0)
123(1)	893(2)	1400(0)	115(1)	885(3)	1402(0)
291(1)	896(0)	1459(3)	281(1)	886(2)	1459(1)
316(0)	897(0)	1459(3)	306(0)	895(0)	1460(1)
316(0)	902(0)	1462(0)	307(0)	895(0)	1464(1)
350(7)	902(0)	1463(0)	331(6)	896(1)	1465(1)
350(2)	924(0)	1539(0)	351(5)	915(0)	1536(0)
350(3)	924(0)	1539(0)	351(5)	915(0)	1536(0)
367(17)	954(0)	1558(0)	367(15)	946(0)	1567(0)
367(16)	956(0)	1558(0)	368(14)	948(0)	1567(0)
504(0)	985(5)	3141(0)	507(0)	990(4)	3135(0)
505(0)	986(5)	3141(0)	507(0)	990(4)	3135(0)
542(0)	1026(8)	3149(0)	531(0)	1028(5)	3143(0)
542(0)	1027(8)	3149(0)	532(0)	1029(5)	3144(0)
618(0)	1075(0)	3158(70)	614(0)	1075(0)	3149(98)
618(0)	1075(0)	3158(70)	615(0)	1075(0)	3149(97)
812(0)	1140(6)	3163(16)	786(0)	1141(9)	3154(24)
813(0)	1187(0)	3223(0)	787(0)	1190(0)	3214(0)
818(39)	1187(0)	3223(0)	811(5)	1190(0)	3214(0)
828(0)	1256(0)	3238(6)	812(0)	1255(0)	3231(12)
828(0)	1257(0)	3239(6)	813(13)	1256(0)	3231(12)
847(79)	1282(0)	3249(8)	849(61)	1275(0)	3239(13)

Table S27. Harmonic vibrational frequencies (in cm^{-1}) and infrared intensities (in parentheses, in km/mol) for the $C_6H_6WC_6H_6$ structure **W3-66T**

B3PW91			M06-L		
-212(0)	738(5)	1329(0)	34(0)	740(0)	1326(0)
28(0)	794(0)	1330(0)	100(0)	744(4)	1326(0)
93(0)	804(0)	1396(4)	109(0)	750(0)	1399(0)
109(0)	812(22)	1396(0)	204(2)	765(1)	1399(0)
179(2)	823(3)	1426(0)	218(0)	772(0)	1439(2)
236(42)	883(0)	1427(0)	247(5)	853(0)	1443(0)
250(0)	885(0)	1440(0)	273(0)	866(1)	1443(0)
268(7)	898(0)	1465(0)	277(0)	892(0)	1456(0)
299(0)	903(0)	1467(0)	298(0)	895(0)	1461(6)
334(0)	951(58)	1497(4)	340(0)	952(64)	1489(0)
345(0)	955(0)	1549(0)	350(0)	955(0)	1491(0)
414(0)	960(74)	3190(0)	390(47)	958(62)	3190(0)
431(0)	967(0)	3196(0)	460(0)	969(0)	3190(18)
525(21)	1009(3)	3196(3)	517(16)	1005(4)	3190(0)
556(0)	1011(17)	3197(0)	565(0)	1010(0)	3191(2)
578(5)	1013(0)	3207(3)	573(0)	1020(13)	3209(0)
589(0)	1017(0)	3208(0)	580(0)	1021(0)	3217(0)
600(0)	1112(0)	3211(19)	601(9)	1121(0)	3219(13)
610(0)	1141(0)	3211(0)	616(0)	1137(0)	3221(0)
667(0)	1143(0)	3229(4)	667(0)	1139(0)	3226(11)
703(0)	1144(0)	3230(0)	709(0)	1143(0)	3227(0)
717(0)	1161(0)	3232(0)	715(0)	1143(0)	3231(0)
730(185)	1263(0)	3233(0)	739(152)	1267(0)	3231(0)

Table S28. Harmonic vibrational frequencies (in cm^{-1}) and infrared intensities (in parentheses, in km/mol) for the $C_6H_6MnC_6H_6$ structure **Mn1-66Q**

B3PW91			M06-L		
26(0)	810(1)	1351(0)	43(0)	790(0)	1351(0)
72(0)	810(1)	1351(0)	47(0)	791(0)	1359(0)
72(0)	871(0)	1355(0)	52(0)	844(0)	1370(0)
84(0)	871(0)	1466(0)	53(0)	844(0)	1468(0)
84(0)	872(0)	1466(0)	55(0)	848(0)	1468(1)
190(0)	872(0)	1467(5)	168(63)	848(0)	1469(0)
215(75)	927(0)	1467(5)	170(1)	906(0)	1469(1)
218(0)	928(0)	1475(0)	172(1)	907(0)	1477(0)
218(0)	977(82)	1475(0)	183(0)	977(86)	1477(0)
379(0)	982(0)	1547(0)	372(0)	983(0)	1553(0)
379(0)	1015(0)	1547(0)	372(0)	1013(0)	1553(0)
414(0)	1019(0)	3192(0)	415(0)	1017(0)	3194(0)
414(0)	1019(0)	3193(0)	416(0)	1021(0)	3195(0)
603(0)	1020(23)	3199(0)	592(0)	1021(20)	3201(0)
603(0)	1020(23)	3199(0)	598(0)	1022(0)	3201(0)
611(0)	1022(0)	3200(0)	598(0)	1022(20)	3202(0)
612(0)	1130(0)	3200(0)	602(0)	1135(0)	3202(0)
612(0)	1130(0)	3213(0)	606(0)	1136(0)	3215(0)
633(0)	1155(0)	3213(0)	606(0)	1155(0)	3215(24)
696(117)	1157(0)	3214(20)	684(99)	1157(0)	3215(0)
718(0)	1165(0)	3214(20)	702(0)	1170(0)	3216(26)
801(0)	1165(0)	3223(1)	784(0)	1171(0)	3224(0)
801(0)	1347(0)	3223(0)	785(0)	1351(0)	3224(0)

Table S29. Harmonic vibrational frequencies (in cm^{-1}) and infrared intensities (in parentheses, in km/mol) for the $C_6H_6MnC_6H_6$ structure **Mn2-66D**

B3PW91			M06-L		
27(0)	863(0)	1353(0)	-117(6)	843(5)	1351(0)
108(0)	882(3)	1391(11)	35(0)	857(0)	1394(22)
122(0)	887(6)	1400(0)	78(0)	865(0)	1410(0)
132(0)	901(0)	1463(10)	123(0)	881(3)	1463(5)
138(0)	912(0)	1465(0)	125(0)	890(0)	1465(0)
234(0)	929(0)	1479(4)	184(0)	913(0)	1480(1)
239(0)	947(0)	1480(0)	237(0)	918(1)	1481(0)
282(2)	947(0)	1518(3)	242(0)	923(0)	1511(5)
327(5)	997(0)	1533(0)	243(0)	999(0)	1526(0)
344(101)	997(19)	1547(0)	338(83)	1000(21)	1547(0)
385(21)	1007(0)	1548(0)	370(34)	1000(16)	1551(0)
402(0)	1014(9)	3175(0)	395(0)	1002(0)	3164(0)
431(0)	1015(0)	3176(6)	430(0)	1017(0)	3164(12)
551(0)	1017(10)	3180(0)	556(0)	1019(8)	3169(0)
608(0)	1038(0)	3181(0)	583(26)	1040(0)	3170(0)
612(0)	1039(12)	3185(1)	588(0)	1041(10)	3173(4)
614(0)	1145(0)	3186(0)	602(0)	1144(0)	3175(0)
620(0)	1150(0)	3193(0)	609(0)	1151(0)	3180(88)
623(11)	1155(0)	3193(61)	615(0)	1156(0)	3180(0)
756(78)	1157(0)	3200(0)	747(46)	1156(1)	3188(67)
773(0)	1161(1)	3201(46)	762(0)	1157(1)	3188(0)
824(0)	1166(0)	3205(16)	808(0)	1169(0)	3191(25)
840(1)	1352(0)	3206(0)	822(0)	1351(0)	3194(0)

Table S30. Harmonic vibrational frequencies (in cm^{-1}) and infrared intensities (in parentheses, in km/mol) for the $C_5H_5MnC_7H_7$ structure **Mn3-57Q**

B3PW91			M06-L		
7(0)	855(0)	1388(0)	18(0)	844(0)	1389(0)
72(0)	855(0)	1388(0)	61(2)	844(0)	1393(0)
72(0)	860(6)	1391(0)	61(2)	847(0)	1393(0)
92(1)	864(0)	1461(3)	70(0)	847(0)	1463(1)
92(1)	864(0)	1461(3)	70(0)	858(0)	1463(1)
173(0)	880(0)	1465(0)	180(0)	859(0)	1470(0)
186(1)	880(0)	1465(0)	190(1)	864(7)	1470(0)
186(1)	899(0)	1500(0)	190(1)	897(0)	1508(0)
286(0)	899(0)	1500(0)	292(2)	897(0)	1508(0)
286(0)	927(0)	1528(0)	294(0)	919(0)	1528(0)
296(40)	927(0)	1528(0)	295(29)	919(0)	1528(0)
445(0)	970(7)	3175(0)	454(0)	975(7)	3176(0)
445(0)	970(7)	3175(0)	455(0)	975(7)	3176(0)
494(0)	1027(19)	3184(0)	477(0)	1030(15)	3185(0)
495(0)	1027(19)	3184(0)	478(0)	1030(15)	3185(0)
628(0)	1072(0)	3196(8)	621(0)	1076(0)	3197(11)
629(0)	1073(0)	3196(8)	622(0)	1077(0)	3197(11)
714(181)	1148(0)	3204(1)	715(139)	1151(0)	3205(1)
754(0)	1184(0)	3216(0)	729(0)	1189(0)	3216(0)
754(0)	1184(0)	3216(0)	730(0)	1189(0)	3217(0)
773(145)	1261(0)	3233(8)	765(118)	1261(0)	3235(11)
790(4)	1261(0)	3233(8)	783(2)	1262(0)	3235(11)
790(4)	1280(0)	3247(1)	783(2)	1276(0)	3249(1)

Table S31. Harmonic vibrational frequencies (in cm^{-1}) and infrared intensities (in parentheses, in km/mol) for the $C_6H_6TcC_6H_6$ structure **Tc1-66D**

B3PW91			M06-L		
39(0)	843(0)	1344(0)	43(0)	830(0)	1342(0)
105(3)	871(0)	1345(0)	104(3)	847(0)	1343(0)
147(1)	877(6)	1406(0)	145(1)	868(3)	1418(0)
153(41)	899(0)	1438(0)	160(0)	871(0)	1433(0)
167(0)	930(0)	1449(1)	190(29)	912(0)	1450(0)
250(1)	939(0)	1450(0)	242(1)	922(0)	1451(0)
295(32)	944(0)	1471(0)	294(35)	928(0)	1472(0)
319(0)	955(1)	1474(7)	332(0)	941(2)	1478(2)
374(3)	972(58)	1476(0)	362(3)	975(61)	1478(0)
408(1)	986(1)	1515(33)	404(0)	976(43)	1516(23)
421(0)	986(0)	1543(0)	416(0)	985(0)	1551(0)
427(0)	993(26)	3178(3)	437(0)	987(1)	3175(5)
436(20)	1011(0)	3178(3)	438(12)	994(25)	3175(9)
570(70)	1014(1)	3179(9)	565(61)	1007(1)	3175(2)
591(9)	1014(33)	3180(0)	577(12)	1009(0)	3177(0)
609(0)	1021(0)	3186(2)	609(0)	1026(0)	3184(15)
609(0)	1023(14)	3186(17)	610(30)	1027(5)	3185(2)
610(48)	1125(0)	3197(37)	615(0)	1128(0)	3192(51)
628(2)	1152(4)	3198(0)	624(2)	1153(0)	3194(0)
685(74)	1154(0)	3202(13)	674(43)	1154(0)	3198(13)
702(5)	1155(0)	3203(16)	684(5)	1156(2)	3199(22)
817(67)	1161(0)	3228(0)	808(58)	1166(0)	3227(0)
827(5)	1315(22)	3230(7)	817(3)	1313(19)	3228(11)

Table S32. Harmonic vibrational frequencies (in cm^{-1}) and infrared intensities (in parentheses, in km/mol) for the $C_5H_5TcC_7H_7$ structure **Tc2-57D**

B3PW91			M06-L		
35(0)	826(1)	1339(0)	28(0)	830(7)	1383(0)
95(2)	830(19)	1383(3)	56(1)	837(5)	1393(0)
121(1)	836(3)	1386(0)	121(1)	873(11)	1402(1)
232(1)	845(1)	1389(8)	121(0)	879(2)	1445(1)
305(0)	884(1)	1394(0)	278(0)	887(8)	1448(0)
305(0)	898(9)	1402(0)	281(1)	892(0)	1451(0)
345(1)	916(1)	1443(0)	335(3)	901(0)	1458(1)
365(0)	917(4)	1447(1)	342(12)	907(1)	1486(0)
375(6)	924(22)	1457(2)	352(8)	910(0)	1513(4)
404(10)	957(0)	1474(0)	367(3)	937(5)	1521(0)
429(8)	977(0)	1703(9)	387(17)	954(2)	1622(0)
468(5)	984(6)	3122(12)	446(9)	954(2)	3140(1)
511(2)	1008(1)	3124(4)	461(0)	999(2)	3140(3)
594(2)	1016(12)	3136(24)	515(6)	1017(8)	3146(8)
601(1)	1022(11)	3155(13)	559(3)	1025(6)	3156(4)
605(0)	1068(0)	3160(48)	595(2)	1070(0)	3162(58)
614(0)	1075(2)	3161(21)	600(0)	1075(0)	3165(55)
739(32)	1097(1)	3178(13)	740(26)	1135(19)	3171(19)
792(4)	1133(13)	3233(0)	795(1)	1161(0)	3216(1)
792(32)	1150(5)	3241(0)	798(0)	1199(1)	3220(0)
799(13)	1171(0)	3251(1)	807(0)	1244(0)	3235(12)
810(7)	1190(1)	3253(1)	810(0)	1247(0)	3235(10)
811(7)	1277(0)	3265(1)	814(16)	1273(0)	3246(5)

Table S33. Harmonic vibrational frequencies (in cm^{-1}) and infrared intensities (in parentheses, in km/mol) for the $C_5H_5TcC_7H_7$ structure **Tc3-57Q**

B3PW91			M06-L		
-8(0)	816(3)	1362(0)	17(0)	801(0)	1363(0)
52(1)	843(1)	1383(3)	66(1)	827(0)	1387(3)
79(0)	848(0)	1386(0)	68(0)	829(1)	1393(0)
136(0)	849(10)	1394(1)	145(0)	848(0)	1407(1)
222(0)	849(0)	1426(9)	220(0)	851(12)	1430(0)
267(1)	878(1)	1426(2)	256(2)	871(0)	1432(4)
300(1)	888(0)	1453(2)	302(0)	880(1)	1456(0)
305(1)	906(0)	1456(2)	307(1)	888(0)	1456(1)
331(1)	907(10)	1471(5)	328(1)	910(10)	1472(4)
336(4)	922(0)	1508(2)	331(3)	923(0)	1514(1)
398(3)	931(3)	1523(3)	394(3)	933(3)	1527(5)
427(1)	958(1)	3140(3)	423(1)	954(1)	3147(5)
463(4)	1006(6)	3141(3)	479(3)	1004(6)	3148(0)
526(7)	1019(12)	3154(0)	527(5)	1023(11)	3160(1)
586(2)	1021(14)	3159(9)	582(1)	1024(9)	3160(11)
594(0)	1071(0)	3166(27)	592(0)	1074(0)	3169(31)
606(0)	1073(0)	3177(30)	599(0)	1076(0)	3179(19)
690(68)	1135(10)	3181(12)	687(58)	1136(14)	3187(21)
754(3)	1148(1)	3235(0)	748(3)	1150(1)	3232(0)
788(45)	1153(1)	3235(0)	774(10)	1159(1)	3232(0)
802(0)	1224(1)	3249(1)	777(1)	1228(1)	3247(2)
806(33)	1228(0)	3251(1)	791(4)	1235(0)	3250(2)
811(6)	1279(0)	3262(0)	794(43)	1273(0)	3260(0)

Table S34. Harmonic vibrational frequencies (in cm^{-1}) and infrared intensities (in parentheses, in km/mol) for the $C_6H_6ReC_6H_6$ structure **Re1-66D**

B3PW91			M06-L		
23(0)	799(9)	1339(0)	32(0)	786(0)	1336(0)
32(2)	823(45)	1339(0)	97(2)	794(0)	1336(0)
144(1)	835(0)	1410(0)	143(1)	798(0)	1413(0)
159(2)	850(3)	1411(0)	147(1)	817(11)	1416(0)
164(1)	867(0)	1455(4)	201(0)	818(1)	1456(1)
244(0)	909(2)	1455(0)	256(1)	891(2)	1457(0)
264(0)	940(0)	1465(14)	276(0)	925(0)	1463(10)
317(0)	946(1)	1465(0)	280(3)	933(2)	1474(2)
353(1)	951(0)	1466(0)	306(0)	936(0)	1474(0)
363(9)	963(0)	1510(0)	359(0)	964(0)	1512(0)
367(0)	969(47)	1511(0)	370(0)	971(53)	1514(0)
390(55)	969(47)	3179(1)	386(50)	972(43)	3173(38)
467(2)	983(0)	3180(18)	470(0)	985(0)	3173(0)
528(22)	997(4)	3181(5)	513(25)	991(4)	3174(9)
577(0)	1006(0)	3182(4)	575(0)	1001(0)	3175(0)
590(21)	1026(0)	3194(10)	578(0)	1030(0)	3192(0)
603(0)	1028(7)	3199(0)	587(0)	1033(5)	3200(0)
615(3)	1128(1)	3203(27)	611(12)	1132(1)	3202(35)
637(0)	1145(0)	3204(3)	638(0)	1149(0)	3204(0)
765(138)	1145(0)	3213(17)	641(0)	1149(0)	3209(37)
771(0)	1152(0)	3215(0)	772(0)	1150(0)	3210(0)
773(55)	1154(0)	3219(3)	782(144)	1152(0)	3212(6)
790(1)	1290(9)	3220(3)	785(0)	1276(0)	3215(0)

Table S35 Harmonic vibrational frequencies (in cm^{-1}) and infrared intensities (in parentheses, in km/mol) for the $C_5H_5ReC_7H_7$ structure **Re2-57D**

B3PW91			M06-L		
35(1)	820(0)	1334(0)	32(1)	802(9)	1334(0)
91(2)	834(3)	1372(0)	91(2)	825(5)	1370(0)
114(2)	837(20)	1374(0)	110(2)	827(19)	1374(0)
234(1)	845(1)	1382(7)	218(2)	830(1)	1380(1)
304(0)	878(1)	1387(3)	300(0)	861(1)	1390(5)
311(4)	897(10)	1391(0)	313(5)	895(3)	1393(0)
346(3)	911(2)	1425(0)	342(4)	898(10)	1416(2)
358(4)	916(1)	1442(1)	363(5)	902(0)	1442(0)
376(1)	920(24)	1451(3)	385(0)	918(23)	1452(2)
386(9)	960(0)	1466(0)	399(6)	956(0)	1471(1)
420(5)	979(0)	1697(9)	423(5)	974(0)	1691(6)
487(3)	981(6)	3120(19)	482(4)	980(6)	3126(18)
521(1)	1010(0)	3121(3)	533(0)	1008(1)	3127(4)
590(2)	1012(11)	3137(21)	587(2)	1014(8)	3141(20)
601(1)	1015(14)	3155(8)	601(2)	1016(11)	3153(2)
607(0)	1065(0)	3160(21)	606(0)	1065(0)	3159(26)
615(0)	1075(2)	3161(55)	612(0)	1077(2)	3165(63)
743(34)	1087(1)	3178(14)	740(23)	1090(0)	3172(18)
792(1)	1126(16)	3235(0)	764(16)	1127(21)	3230(0)
797(31)	1143(4)	3242(0)	767(0)	1146(4)	3235(0)
798(12)	1165(1)	3252(1)	786(13)	1169(2)	3248(3)
804(2)	1183(0)	3254(1)	790(5)	1181(1)	3250(3)
806(10)	1273(0)	3265(0)	794(0)	1266(0)	3259(1)

Table S36. Harmonic vibrational frequencies (in cm^{-1}) and infrared intensities (in parentheses, in km/mol) for the $C_5H_5ReC_7H_7$ structure **Re3-57Q**

B3PW91			M06-L		
-11(0)	816(18)	1350(0)	23(0)	808(9)	1350(0)
45(1)	836(1)	1378(4)	66(1)	819(0)	1380(4)
78(1)	843(4)	1385(0)	70(0)	828(1)	1391(0)
145(0)	846(9)	1390(2)	149(0)	840(0)	1401(1)
225(0)	846(0)	1404(12)	221(1)	849(14)	1409(7)
282(0)	874(0)	1413(2)	281(0)	867(0)	1417(1)
296(1)	884(0)	1446(2)	303(1)	877(0)	1448(2)
315(1)	892(10)	1447(2)	314(0)	888(1)	1448(0)
339(1)	904(0)	1458(5)	346(2)	894(10)	1460(5)
358(0)	929(2)	1496(1)	356(0)	932(1)	1504(1)
399(4)	935(4)	1517(8)	397(4)	937(4)	1521(10)
430(1)	976(0)	3131(9)	427(1)	974(0)	3141(4)
466(4)	1005(7)	3131(2)	484(4)	1005(7)	3141(4)
524(8)	1015(11)	3151(1)	525(5)	1016(12)	3155(2)
590(1)	1015(15)	3158(9)	585(1)	1018(9)	3159(15)
605(1)	1069(0)	3163(27)	600(0)	1072(0)	3165(28)
605(0)	1072(0)	3175(30)	609(0)	1075(0)	3177(19)
682(64)	1127(13)	3179(19)	678(55)	1129(18)	3184(28)
743(2)	1135(1)	3238(0)	736(3)	1136(0)	3235(0)
792(27)	1136(1)	3239(0)	764(2)	1143(1)	3235(0)
794(1)	1214(2)	3251(0)	777(5)	1219(2)	3249(1)
806(3)	1223(0)	3254(0)	787(2)	1230(0)	3252(1)
815(23)	1275(0)	3264(0)	804(27)	1268(0)	3261(0)

Table S37. Harmonic vibrational frequencies (in cm^{-1}) and infrared intensities (in parentheses, in km/mol) for the $C_6H_6FeC_6H_6$ structure **Fe1-66T**

B3PW91			M06-L		
33(0)	851(2)	1357(0)	46(0)	841(1)	1357(0)
95(0)	852(2)	1360(0)	70(0)	842(1)	1370(0)
95(0)	894(0)	1363(0)	73(0)	861(0)	1375(0)
111(0)	894(0)	1478(8)	103(0)	861(0)	1479(3)
111(0)	919(0)	1478(0)	104(0)	901(0)	1479(0)
202(0)	919(0)	1478(8)	190(0)	901(0)	1480(3)
233(1)	953(0)	1478(0)	193(0)	932(0)	1480(0)
233(1)	954(0)	1534(0)	194(73)	933(0)	1530(0)
236(79)	994(0)	1534(0)	194(0)	995(34)	1530(0)
390(0)	994(30)	1558(0)	382(0)	996(0)	1566(0)
390(0)	1006(0)	1559(0)	383(0)	1003(0)	1566(0)
423(0)	1013(0)	3181(0)	424(0)	1008(0)	3175(0)
423(0)	1030(0)	3182(0)	425(0)	1032(0)	3175(0)
611(0)	1030(0)	3188(0)	599(0)	1033(0)	3183(0)
611(0)	1032(14)	3188(0)	604(0)	1034(11)	3183(0)
614(0)	1032(14)	3189(0)	604(0)	1034(11)	3184(0)
614(0)	1155(0)	3189(0)	610(0)	1157(0)	3184(0)
619(0)	1155(0)	3202(0)	611(0)	1158(0)	3195(0)
640(0)	1159(0)	3202(0)	612(0)	1159(0)	3195(56)
724(110)	1162(0)	3203(41)	719(80)	1161(0)	3196(0)
744(0)	1166(0)	3203(41)	734(0)	1171(0)	3196(61)
837(0)	1166(0)	3210(9)	828(0)	1171(0)	3202(14)
837(0)	1357(0)	3211(0)	829(0)	1357(0)	3203(0)

Table S38. Harmonic vibrational frequencies (in cm^{-1}) and infrared intensities (in parentheses, in km/mol) for the $C_5H_5FeC_7H_7$ structure **Fe2-57S**

B3PW91			M06-L		
37(0)	848(3)	1349(1)	35(0)	836(9)	1349(1)
132(1)	849(1)	1395(12)	131(1)	846(0)	1397(6)
172(1)	873(13)	1403(1)	172(1)	871(11)	1404(2)
256(1)	902(6)	1404(0)	262(0)	888(1)	1407(0)
300(0)	914(0)	1419(0)	300(0)	899(1)	1421(0)
300(1)	915(0)	1453(0)	304(0)	905(4)	1451(0)
344(1)	923(1)	1456(2)	357(1)	912(1)	1452(1)
362(0)	946(17)	1467(2)	373(0)	934(0)	1466(1)
417(6)	950(5)	1495(0)	438(4)	944(21)	1496(0)
426(11)	960(0)	1506(6)	441(5)	959(0)	1499(4)
444(8)	991(0)	1725(15)	453(15)	982(0)	1722(18)
491(25)	1005(2)	3118(13)	506(29)	996(2)	3114(17)
525(18)	1020(1)	3119(11)	543(16)	1021(2)	3114(13)
604(4)	1023(8)	3133(11)	600(2)	1023(5)	3131(8)
610(1)	1033(8)	3142(11)	606(1)	1035(4)	3133(22)
613(1)	1074(0)	3146(52)	611(0)	1073(0)	3138(53)
615(1)	1077(0)	3158(52)	617(2)	1079(0)	3162(63)
749(33)	1119(0)	3179(20)	748(28)	1119(0)	3167(25)
802(7)	1148(10)	3229(0)	798(4)	1147(14)	3220(0)
826(32)	1156(4)	3235(1)	814(8)	1159(4)	3225(1)
837(15)	1191(1)	3248(5)	825(6)	1192(0)	3239(9)
845(4)	1207(0)	3248(5)	828(0)	1205(0)	3240(10)
847(0)	1282(0)	3262(2)	834(8)	1274(0)	3251(3)

Table S39. Harmonic vibrational frequencies (in cm^{-1}) and infrared intensities (in parentheses, in km/mol) for the $C_6H_6FeC_6H_6$ structure **Fe3-66S**

B3PW91			M06-L		
15(0)	873(3)	1351(10)	13(0)	862(3)	1347(5)
123(2)	889(13)	1354(0)	119(2)	875(8)	1349(0)
165(0)	909(1)	1394(8)	166(0)	896(3)	1394(5)
240(3)	914(0)	1427(1)	246(2)	905(2)	1431(0)
291(2)	918(4)	1441(0)	302(2)	908(5)	1443(1)
309(0)	927(1)	1466(2)	329(0)	911(1)	1464(1)
353(10)	939(2)	1468(4)	377(10)	920(1)	1466(2)
386(5)	963(2)	1483(3)	404(3)	940(1)	1482(0)
416(3)	966(0)	1537(0)	423(4)	959(1)	1533(0)
429(6)	976(18)	1554(0)	435(7)	978(16)	1552(0)
429(36)	1005(7)	1658(2)	445(31)	1007(9)	1662(6)
470(1)	1011(0)	3149(16)	468(1)	1009(0)	3148(19)
513(32)	1023(3)	3152(2)	517(27)	1019(0)	3151(3)
586(2)	1025(1)	3166(15)	592(5)	1023(3)	3166(2)
622(1)	1044(4)	3177(24)	621(1)	1047(2)	3172(40)
627(0)	1057(6)	3187(0)	627(0)	1059(7)	3177(0)
642(0)	1118(0)	3187(44)	635(0)	1117(0)	3181(33)
649(1)	1123(3)	3192(20)	646(1)	1120(2)	3184(1)
672(4)	1137(1)	3193(1)	673(4)	1139(1)	3187(3)
729(43)	1159(0)	3197(3)	733(40)	1160(0)	3193(39)
802(25)	1165(0)	3207(14)	802(14)	1161(0)	3196(24)
846(1)	1165(0)	3207(16)	841(1)	1166(0)	3196(25)
849(3)	1323(0)	3217(7)			

Table S40. Harmonic vibrational frequencies (in cm^{-1}) and infrared intensities (in parentheses, in km/mol) for the $C_5H_5RuC_7H_7$ structure **Ru1-57S**

B3PW91			M06-L		
20(0)	844(5)	1346(1)	0(0)	832(6)	1347(1)
122(3)	851(2)	1388(4)	119(3)	849(2)	1391(2)
147(2)	884(9)	1394(7)	139(2)	879(7)	1396(4)
259(1)	901(5)	1400(0)	257(0)	891(1)	1405(0)
305(0)	909(0)	1408(0)	302(0)	902(3)	1412(1)
314(0)	917(0)	1433(0)	312(0)	903(2)	1435(0)
357(2)	927(1)	1448(2)	357(3)	917(0)	1448(1)
368(6)	938(18)	1460(2)	365(3)	935(14)	1461(1)
386(0)	958(5)	1485(0)	387(0)	950(8)	1484(3)
408(9)	963(0)	1486(3)	416(8)	962(0)	1490(0)
418(17)	995(0)	1727(17)	422(15)	988(0)	1727(22)
483(5)	1004(3)	3115(21)	483(6)	1000(3)	3114(21)
516(12)	1017(8)	3116(9)	527(11)	1016(2)	3115(12)
603(2)	1019(1)	3135(9)	599(1)	1020(5)	3133(8)
606(0)	1027(9)	3141(8)	605(0)	1030(6)	3135(27)
610(3)	1069(0)	3145(57)	609(3)	1070(0)	3140(50)
616(1)	1075(0)	3160(56)	617(1)	1078(0)	3164(64)
747(34)	1110(0)	3174(23)	747(28)	1112(0)	3167(26)
806(5)	1137(10)	3231(0)	800(3)	1138(15)	3224(0)
817(34)	1155(4)	3235(1)	808(9)	1158(5)	3227(1)
831(7)	1179(0)	3247(5)	815(3)	1180(0)	3241(9)
837(0)	1201(0)	3249(5)	821(0)	1200(0)	3243(9)
837(6)	1280(0)	3260(3)	828(12)	1273(0)	3253(3)

Table S41. Harmonic vibrational frequencies (in cm^{-1}) and infrared intensities (in parentheses, in km/mol) for the $C_6H_6RuC_6H_6$ structure **Ru2-66S**

B3PW91			M06-L		
26(0)	874(3)	1333(7)	28(0)	868(2)	1332(4)
115(3)	883(9)	1351(0)	111(3)	874(6)	1349(0)
139(2)	910(0)	1380(9)	134(1)	900(1)	1382(6)
249(2)	916(1)	1416(0)	246(2)	908(1)	1411(0)
303(4)	924(3)	1419(0)	301(3)	911(2)	1433(0)
328(13)	929(0)	1450(1)	331(13)	917(0)	1452(1)
347(0)	936(0)	1460(4)	354(0)	927(1)	1462(1)
373(11)	958(0)	1476(3)	375(10)	943(0)	1478(1)
401(12)	962(0)	1527(0)	400(9)	955(0)	1527(0)
418(1)	973(10)	1552(0)	422(1)	974(10)	1551(0)
428(3)	996(10)	1673(11)	429(3)	999(12)	1675(16)
470(0)	1010(2)	3146(23)	469(0)	1011(0)	3147(26)
493(17)	1018(2)	3149(1)	498(15)	1017(1)	3150(2)
581(0)	1020(0)	3166(16)	584(1)	1020(2)	3168(0)
615(1)	1037(4)	3177(22)	613(1)	1041(2)	3173(41)
626(0)	1048(10)	3188(44)	626(0)	1049(11)	3181(0)
637(0)	1101(1)	3188(0)	630(0)	1101(0)	3184(33)
645(1)	1112(2)	3193(25)	649(1)	1111(2)	3188(2)
679(4)	1133(1)	3194(1)	680(4)	1136(1)	3192(5)
731(45)	1157(0)	3198(4)	736(40)	1159(0)	3195(41)
804(32)	1157(0)	3208(13)	801(20)	1159(0)	3201(21)
852(2)	1162(0)	3208(15)	845(1)	1161(0)	3201(20)
862(2)	1316(0)	3218(7)	855(2)	1314(0)	3211(11)

Table S42. Harmonic vibrational frequencies (in cm^{-1}) and infrared intensities (in parentheses, in km/mol) for the $C_5H_5RuC_7H_7$ structure **Ru3-57T**

B3PW91			M06-L		
29(0)	830(1)	1379(0)	30(0)	813(2)	1381(0)
83(1)	846(8)	1386(0)	76(1)	843(7)	1391(0)
119(1)	864(0)	1388(0)	112(1)	864(0)	1395(0)
174(2)	883(7)	1430(8)	172(3)	884(8)	1442(3)
202(0)	902(0)	1449(2)	192(0)	884(0)	1451(1)
286(0)	904(0)	1452(5)	279(0)	886(0)	1454(1)
314(1)	906(2)	1455(1)	301(0)	900(0)	1463(0)
340(0)	914(1)	1456(0)	337(1)	903(2)	1474(1)
352(3)	921(5)	1487(6)	363(3)	926(1)	1493(4)
356(4)	929(1)	1525(0)	369(4)	927(5)	1531(0)
404(4)	944(0)	1528(0)	401(3)	937(0)	1534(0)
448(4)	977(1)	3144(4)	443(4)	965(1)	3147(1)
479(10)	1015(1)	3144(2)	482(9)	1013(1)	3149(2)
518(1)	1018(9)	3157(0)	519(1)	1021(7)	3157(5)
585(0)	1022(10)	3159(1)	584(0)	1023(8)	3161(8)
593(0)	1068(0)	3168(42)	589(0)	1070(0)	3170(45)
597(4)	1072(0)	3174(36)	601(2)	1074(0)	3171(30)
685(69)	1135(10)	3182(20)	680(58)	1137(16)	3188(21)
763(4)	1159(0)	3232(0)	757(4)	1162(0)	3225(0)
813(50)	1166(0)	3233(0)	803(0)	1173(0)	3227(0)
819(0)	1238(0)	3247(4)	809(25)	1243(0)	3242(8)
826(2)	1243(0)	3248(4)	810(1)	1250(0)	3243(7)
829(0)	1280(0)	3259(3)	812(1)	1273(0)	3253(3)

Table S43. Harmonic vibrational frequencies (in cm^{-1}) and infrared intensities (in parentheses, in km/mol) for the $C_5H_5OsC_7H_7$ structure **Os1-57S**

B3PW91			M06-L		
2(0)	845(5)	1343(1)	-13(0)	838(12)	1344(1)
120(3)	858(5)	1380(1)	118(3)	856(3)	1383(1)
141(3)	894(7)	1389(9)	135(2)	886(2)	1391(5)
264(1)	900(6)	1395(0)	262(1)	894(5)	1400(0)
305(0)	905(1)	1401(0)	302(0)	901(4)	1406(1)
320(3)	917(0)	1419(0)	321(3)	904(1)	1422(0)
343(5)	932(2)	1443(2)	338(5)	923(1)	1444(1)
371(4)	936(17)	1453(2)	376(4)	936(15)	1454(1)
393(12)	962(4)	1472(4)	403(11)	956(7)	1472(4)
397(6)	963(0)	1479(0)	407(6)	963(0)	1484(0)
409(2)	999(0)	1725(21)	412(1)	995(0)	1727(27)
509(1)	1002(3)	3110(29)	507(2)	1001(4)	3112(27)
515(7)	1015(7)	3111(8)	528(6)	1018(5)	3112(12)
601(2)	1021(6)	3138(5)	601(2)	1023(0)	3131(10)
606(0)	1027(5)	3140(10)	607(0)	1026(9)	3134(46)
609(2)	1067(0)	3144(61)	609(2)	1068(0)	3142(33)
619(0)	1075(0)	3163(56)	621(1)	1077(0)	3167(49)
749(35)	1103(0)	3174(24)	751(29)	1106(0)	3168(42)
812(4)	1131(13)	3233(0)	805(2)	1134(18)	3226(0)
825(23)	1152(4)	3237(1)	811(0)	1155(5)	3229(1)
829(0)	1171(0)	3249(4)	818(6)	1173(0)	3243(8)
835(0)	1195(0)	3250(4)	820(0)	1195(0)	3243(8)
844(12)	1277(0)	3261(3)	834(5)	1269(0)	3254(4)

Table S44. Harmonic vibrational frequencies (in cm^{-1}) and infrared intensities (in parentheses, in km/mol) for the $C_6H_6OsC_6H_6$ structure **Os2-66S**

B3PW91			M06-L		
24(0)	876(7)	1316(6)	23(0)	865(5)	1315(4)
115(3)	885(5)	1347(0)	113(3)	881(5)	1343(0)
138(3)	905(1)	1371(9)	134(2)	901(3)	1373(5)
257(2)	918(1)	1392(1)	255(2)	908(0)	1387(1)
316(7)	922(4)	1425(1)	313(6)	910(2)	1437(1)
342(9)	940(2)	1444(1)	348(9)	930(0)	1444(2)
355(12)	940(0)	1462(3)	360(11)	934(3)	1463(1)
370(1)	956(1)	1464(2)	376(1)	942(0)	1467(0)
386(5)	957(0)	1521(0)	388(5)	953(0)	1521(0)
431(2)	976(5)	1535(1)	431(1)	976(6)	1537(1)
437(0)	997(11)	1677(19)	438(0)	1000(14)	1679(26)
477(11)	1016(5)	3142(29)	480(0)	1013(3)	3145(30)
480(0)	1021(3)	3145(0)	485(10)	1023(4)	3147(1)
595(0)	1022(3)	3166(14)	597(0)	1025(1)	3167(3)
617(2)	1030(1)	3176(21)	617(1)	1031(2)	3172(37)
621(1)	1043(13)	3188(41)	621(2)	1044(15)	3183(34)
635(7)	1086(1)	3190(1)	630(7)	1087(0)	3184(2)
641(1)	1103(1)	3192(31)	646(1)	1102(2)	3189(2)
692(4)	1130(1)	3196(0)	694(4)	1133(1)	3190(9)
735(45)	1147(0)	3198(6)	736(40)	1149(0)	3196(44)
818(38)	1158(0)	3209(14)	815(23)	1157(0)	3203(18)
859(1)	1160(0)	3212(10)	847(1)	1162(0)	3206(13)
860(4)	1310(0)	3228(4)	853(2)	1307(0)	3224(6)

Table S45. Harmonic vibrational frequencies (in cm^{-1}) and infrared intensities (in parentheses, in km/mol) for the $C_5H_5OsC_7H_7$ structure **Os3-57T**

B3PW91			M06-L		
31(0)	833(1)	1371(0)	34(0)	828(20)	1373(0)
85(2)	849(7)	1383(0)	79(2)	849(6)	1387(0)
118(2)	856(1)	1387(0)	114(2)	855(0)	1393(0)
171(1)	884(5)	1418(9)	172(2)	885(5)	1430(4)
213(0)	898(2)	1435(9)	206(0)	886(0)	1444(1)
303(2)	903(2)	1441(0)	298(2)	887(2)	1445(3)
317(0)	904(0)	1446(2)	314(1)	904(1)	1448(1)
343(2)	908(3)	1447(2)	349(1)	904(1)	1457(0)
361(0)	923(2)	1483(9)	369(2)	911(5)	1486(8)
370(3)	927(3)	1514(0)	386(3)	930(3)	1522(1)
404(5)	954(1)	1521(1)	404(4)	947(0)	1525(0)
453(4)	990(0)	3138(9)	448(3)	983(0)	3144(3)
470(6)	1015(8)	3138(1)	476(5)	1016(2)	3145(2)
517(2)	1016(6)	3155(0)	517(1)	1018(6)	3155(6)
586(0)	1020(6)	3158(3)	587(0)	1019(8)	3159(6)
595(0)	1067(0)	3166(41)	591(0)	1069(0)	3168(46)
603(4)	1073(0)	3172(37)	607(3)	1074(0)	3169(34)
677(68)	1130(12)	3181(27)	672(58)	1132(19)	3186(26)
748(3)	1150(0)	3235(0)	740(4)	1152(0)	3226(0)
823(0)	1152(1)	3237(0)	805(0)	1160(0)	3230(0)
825(5)	1230(1)	3249(3)	806(0)	1235(1)	3243(7)
829(36)	1235(0)	3250(3)	818(0)	1243(0)	3244(6)
833(2)	1277(0)	3261(2)	819(1)	1269(0)	3255(3)

Table S46. Harmonic vibrational frequencies (in cm^{-1}) and infrared intensities (in parentheses, in km/mol) for the $C_6H_6CoC_6H_6$ structure **Co1-66D**

B3PW91			M06-L		
-9(0)	865(0)	1357(0)	5(0)	853(0)	1358(0)
42(0)	867(0)	1377(6)	61(0)	857(3)	1387(4)
91(0)	883(7)	1386(0)	102(0)	876(9)	1403(1)
119(0)	913(0)	1460(2)	145(0)	896(0)	1468(1)
167(0)	922(3)	1473(3)	180(0)	903(2)	1469(1)
259(0)	940(32)	1478(4)	266(1)	940(0)	1477(1)
262(1)	960(0)	1483(3)	292(1)	946(26)	1488(0)
264(1)	964(0)	1538(0)	294(2)	964(0)	1536(1)
354(26)	969(1)	1544(2)	359(16)	980(1)	1545(2)
401(0)	999(4)	1564(39)	410(0)	1001(5)	1563(18)
405(3)	1009(4)	1634(3)	411(2)	1012(1)	1631(2)
435(0)	1016(0)	3137(1)	439(0)	1014(3)	3137(1)
445(17)	1020(4)	3152(9)	440(9)	1022(4)	3152(13)
574(1)	1027(9)	3165(1)	576(2)	1026(7)	3169(5)
607(3)	1035(8)	3173(7)	610(3)	1038(5)	3175(7)
615(0)	1037(1)	3184(32)	613(0)	1043(0)	3180(0)
622(2)	1143(10)	3186(0)	617(2)	1146(7)	3186(2)
645(0)	1158(0)	3193(0)	632(0)	1158(0)	3186(0)
686(0)	1161(0)	3193(0)	688(0)	1159(0)	3189(32)
703(64)	1163(0)	3196(17)	704(55)	1165(1)	3198(17)
763(14)	1177(3)	3205(13)	761(14)	1180(1)	3200(21)
813(0)	1190(0)	3206(15)	811(1)	1192(0)	3201(15)
824(108)	1356(0)	3214(6)	827(82)	1351(0)	3206(10)

Table S47. Harmonic vibrational frequencies (in cm^{-1}) and infrared intensities (in parentheses, in km/mol) for the $C_5H_5CoC_7H_7$ structure **Co2-57D**

B3PW91			M06-L		
22(0)	844(0)	1379(4)	1(0)	829(4)	1384(1)
50(1)	845(3)	1385(0)	23(1)	830(0)	1387(2)
76(0)	865(12)	1387(4)	75(0)	868(12)	1388(1)
120(0)	896(1)	1430(3)	117(0)	882(1)	1439(1)
163(1)	897(0)	1453(6)	171(1)	884(2)	1458(0)
258(8)	901(1)	1456(2)	274(4)	888(0)	1461(2)
303(0)	904(0)	1462(0)	333(1)	903(0)	1464(0)
322(1)	906(4)	1465(0)	351(0)	911(5)	1467(0)
347(1)	930(2)	1491(1)	354(1)	935(2)	1501(2)
350(1)	945(3)	1582(1)	364(1)	952(3)	1582(2)
437(1)	978(0)	1664(1)	437(4)	976(0)	1659(0)
439(5)	983(0)	3142(1)	446(2)	985(0)	3148(0)
465(0)	993(2)	3145(2)	477(1)	995(2)	3148(4)
579(21)	1023(12)	3158(0)	594(12)	1027(9)	3160(7)
596(3)	1028(16)	3159(2)	598(3)	1028(12)	3161(0)
611(3)	1071(0)	3169(38)	611(0)	1074(0)	3175(37)
617(0)	1072(1)	3177(33)	611(10)	1075(0)	3177(27)
723(79)	1145(3)	3182(5)	723(62)	1146(8)	3188(17)
788(20)	1173(3)	3229(0)	782(4)	1178(3)	3226(0)
801(0)	1189(1)	3230(0)	795(0)	1189(1)	3226(0)
806(63)	1263(0)	3244(5)	804(44)	1265(0)	3241(7)
819(2)	1270(0)	3247(4)	809(2)	1273(1)	3245(6)
825(2)	1281(0)	3258(4)	826(0)	1276(0)	3255(4)

Table S48. Harmonic vibrational frequencies (in cm^{-1}) and infrared intensities (in parentheses, in km/mol) for the $C_6H_6CoC_6H_6$ structure **Co3-66Q**

B3PW91			M06-L		
29(0)	775(5)	1353(0)	41(0)	706(4)	1345(0)
72(0)	792(3)	1354(0)	87(0)	776(80)	1353(0)
93(0)	792(0)	1360(24)	98(0)	779(0)	1354(0)
104(0)	796(5)	1372(0)	150(107)	819(0)	1376(19)
172(0)	799(0)	1376(4)	179(0)	828(4)	1383(0)
180(0)	829(3749)	1423(2122)	184(0)	892(1490)	1463(0)
184(0)	940(6)	1466(0)	191(0)	912(0)	1463(0)
189(233)	948(0)	1469(0)	191(0)	920(0)	1465(1421)
189(0)	949(0)	1476(0)	198(1)	920(0)	1481(1)
204(0)	953(1)	1476(11)	216(0)	924(0)	1484(0)
334(0)	987(0)	1543(0)	293(0)	951(0)	1582(0)
369(0)	988(52)	3184(137)	356(31)	989(0)	3179(0)
417(5)	990(0)	3184(0)	417(0)	992(51)	3185(0)
431(0)	1018(0)	3185(11)	442(0)	993(0)	3187(0)
581(1)	1018(0)	3187(0)	579(42)	1014(1)	3187(2)
611(97)	1023(0)	3198(0)	580(0)	1015(0)	3200(48)
616(0)	1041(0)	3204(1)	593(3)	1038(0)	3201(0)
623(1)	1041(6)	3213(0)	601(0)	1039(3)	3205(0)
657(0)	1119(528)	3214(24)	637(0)	1148(328)	3205(43)
661(0)	1160(0)	3221(0)	658(262)	1159(0)	3227(15)
697(263)	1161(0)	3221(11)	669(0)	1162(0)	3227(0)
714(0)	1162(1)	3225(47)	670(0)	1182(0)	3229(40)
765(0)	1300(0)	3227(0)	701(0)	1296(0)	3231(0)

Table S49. Harmonic vibrational frequencies (in cm^{-1}) and infrared intensities (in parentheses, in km/mol) for the $C_6H_6RhC_6H_6$ structure **Rh1-66D**

B3PW91			M06-L		
11(0)	855(0)	1359(0)	8(0)	840(0)	1359(0)
57(0)	871(2)	1369(0)	72(1)	862(2)	1381(0)
88(1)	914(9)	1383(5)	88(0)	898(11)	1390(5)
136(1)	933(0)	1461(2)	135(0)	914(0)	1465(1)
144(0)	937(2)	1473(6)	136(0)	925(1)	1474(3)
195(2)	938(21)	1483(2)	199(3)	942(18)	1485(2)
208(0)	959(0)	1484(5)	212(0)	955(0)	1488(0)
231(1)	969(1)	1551(0)	212(1)	962(1)	1550(0)
314(21)	971(0)	1563(3)	315(13)	978(0)	1560(1)
381(0)	997(3)	1567(9)	383(1)	998(4)	1564(6)
396(2)	1008(4)	1641(2)	400(1)	1006(0)	1637(1)
434(0)	1008(0)	3125(0)	435(1)	1013(4)	3134(0)
436(3)	1021(4)	3142(12)	443(1)	1024(4)	3151(12)
577(2)	1023(6)	3163(1)	577(2)	1025(4)	3169(3)
602(0)	1040(6)	3171(8)	597(0)	1042(4)	3175(8)
607(1)	1041(0)	3182(33)	602(0)	1043(0)	3187(0)
610(6)	1147(8)	3190(0)	611(6)	1148(6)	3188(33)
625(0)	1163(0)	3193(19)	617(1)	1162(0)	3194(1)
683(0)	1164(0)	3197(1)	686(0)	1165(0)	3196(2)
714(82)	1167(0)	3200(0)	711(62)	1170(1)	3200(18)
750(30)	1178(0)	3210(8)	742(20)	1180(0)	3206(9)
819(0)	1190(1)	3213(8)	817(0)	1193(1)	3212(9)
843(73)	1358(0)	3220(5)	839(72)	1356(0)	3218(7)

Table S50. Harmonic vibrational frequencies (in cm^{-1}) and infrared intensities (in parentheses, in km/mol) for the $C_5H_5RhC_7H_7$ structure **Rh2-57D**

B3PW91			M06-L		
18(0)	835(2)	1370(0)	22(0)	820(2)	1371(0)
94(1)	842(10)	1377(0)	92(1)	839(6)	1381(0)
122(1)	853(0)	1383(1)	117(1)	851(0)	1391(1)
204(4)	887(9)	1393(0)	203(4)	886(3)	1405(0)
205(0)	904(2)	1430(6)	206(0)	888(9)	1433(3)
256(1)	906(3)	1438(5)	259(1)	892(0)	1446(4)
272(6)	908(0)	1450(3)	268(6)	901(0)	1452(1)
310(4)	910(1)	1462(1)	310(4)	910(2)	1465(0)
325(3)	926(2)	1483(1)	333(2)	917(3)	1487(3)
351(5)	932(2)	1522(3)	354(5)	937(2)	1524(2)
406(1)	963(1)	1543(1)	403(1)	958(1)	1547(3)
430(1)	982(0)	3131(9)	428(1)	976(1)	3133(8)
472(10)	1018(9)	3131(3)	480(9)	1021(7)	3134(4)
525(6)	1027(11)	3152(2)	526(5)	1030(4)	3153(5)
586(3)	1032(3)	3157(19)	586(2)	1032(6)	3155(8)
593(1)	1069(0)	3164(25)	590(1)	1072(0)	3160(43)
604(4)	1070(0)	3178(29)	613(4)	1073(0)	3173(27)
703(57)	1138(7)	3183(22)	701(51)	1141(10)	3190(25)
774(1)	1148(1)	3234(0)	768(1)	1149(1)	3229(1)
795(31)	1156(2)	3236(1)	780(3)	1160(2)	3231(1)
806(41)	1223(0)	3248(4)	795(42)	1230(0)	3244(6)
832(1)	1234(2)	3255(3)	818(0)	1239(2)	3253(4)
832(0)	1281(0)	3265(2)	819(0)	1276(0)	3263(2)

Table S51. Harmonic vibrational frequencies (in cm^{-1}) and infrared intensities (in parentheses, in km/mol) for the $C_6H_6RhC_6H_6$ structure **Rh3-66Q**

B3PW91			M06-L		
38(0)	819(5)	1352(0)	41(0)	796(5)	1353(0)
68(0)	829(13)	1352(0)	75(0)	802(20)	1353(0)
86(1)	842(26)	1374(38)	85(0)	823(3)	1392(26)
98(0)	859(1)	1399(3)	90(1)	834(0)	1398(12)
104(0)	902(373)	1450(8)	114(0)	862(143)	1441(12)
160(1)	941(79)	1452(4)	159(1)	930(1)	1458(2)
192(0)	942(2)	1455(3)	186(0)	932(3)	1460(1)
233(0)	946(18)	1490(0)	218(0)	939(0)	1490(54)
287(29)	954(1)	1490(47)	267(19)	939(253)	1491(0)
307(1)	968(13)	1526(387)	288(1)	958(1)	1529(337)
364(1)	973(24)	1581(1)	358(3)	971(13)	1584(1)
434(0)	982(8)	3178(12)	430(0)	977(23)	3181(9)
446(4)	1001(3)	3179(0)	438(3)	985(9)	3183(0)
577(1)	1012(0)	3185(5)	575(0)	1014(0)	3186(4)
577(0)	1023(9)	3186(1)	576(3)	1019(3)	3187(1)
599(4)	1040(0)	3195(20)	588(1)	1041(0)	3199(18)
602(2)	1044(3)	3196(1)	596(2)	1044(1)	3199(1)
612(2)	1119(7)	3203(14)	607(0)	1114(6)	3205(17)
622(1)	1157(29)	3203(20)	616(0)	1159(15)	3205(26)
678(91)	1159(1)	3212(8)	657(71)	1161(1)	3217(10)
684(2)	1164(41)	3213(11)	662(2)	1168(50)	3217(13)
755(8)	1173(1)	3221(3)	738(12)	1179(1)	3224(4)
759(3)	1331(61)	3222(2)	745(1)	1326(26)	3225(2)

Table S52. Harmonic vibrational frequencies (in cm^{-1}) and infrared intensities (in parentheses, in km/mol) for the $C_5H_5IrC_7H_7$ structure **Ir1-57D**

B3PW91			M06-L		
29(0)	844(2)	1364(0)	30(0)	831(2)	1363(0)
101(2)	850(2)	1376(0)	100(2)	844(0)	1381(0)
127(2)	852(10)	1385(1)	124(2)	853(8)	1394(1)
213(0)	891(8)	1395(0)	212(4)	893(11)	1402(0)
214(4)	892(3)	1418(6)	214(0)	894(2)	1420(3)
293(2)	910(2)	1420(5)	296(2)	895(1)	1424(3)
318(3)	915(0)	1446(3)	324(2)	899(0)	1449(1)
327(2)	915(2)	1452(2)	327(1)	914(1)	1455(1)
356(1)	930(1)	1473(3)	364(1)	916(1)	1476(5)
372(4)	931(1)	1507(2)	381(4)	941(2)	1511(1)
415(1)	982(2)	1534(3)	415(1)	980(2)	1537(6)
442(1)	1010(2)	3120(21)	441(1)	1007(0)	3124(21)
473(8)	1019(7)	3121(3)	486(7)	1021(6)	3125(4)
524(6)	1021(11)	3152(1)	528(5)	1025(8)	3154(4)
591(1)	1037(4)	3159(19)	588(1)	1037(4)	3156(1)
595(2)	1070(0)	3164(25)	594(2)	1073(0)	3161(50)
623(5)	1071(0)	3178(38)	632(5)	1074(0)	3173(31)
690(53)	1133(7)	3181(23)	687(46)	1135(1)	3187(31)
757(1)	1135(3)	3239(0)	750(1)	1137(14)	3233(0)
813(22)	1140(1)	3241(0)	801(3)	1144(1)	3235(1)
827(27)	1218(0)	3252(3)	813(0)	1222(0)	3248(5)
827(0)	1225(3)	3259(1)	814(11)	1229(2)	3256(3)
836(11)	1278(0)	3268(2)	827(21)	1272(0)	3265(2)

Table S53. Harmonic vibrational frequencies (in cm^{-1}) and infrared intensities (in parentheses, in km/mol) for the $C_6H_6IrC_6H_6$ structure **Ir2-66D**

B3PW91			M06-L		
11(0)	873(5)	1354(0)	20(0)	854(1)	1351(0)
54(1)	885(60)	1375(11)	58(1)	886(68)	1373(9)
96(1)	907(7)	1384(1)	96(1)	909(2)	1396(1)
144(1)	926(7)	1435(2)	149(1)	911(10)	1441(1)
174(0)	929(3)	1466(6)	160(0)	918(1)	1467(3)
212(1)	931(1)	1474(2)	206(2)	922(9)	1478(2)
216(0)	957(1)	1477(5)	220(0)	946(0)	1480(3)
255(1)	965(0)	1538(1)	244(1)	964(2)	1539(1)
338(6)	968(0)	1549(0)	338(4)	979(0)	1548(0)
384(4)	996(3)	1566(4)	386(0)	998(5)	1565(3)
385(0)	997(7)	1657(1)	387(3)	1001(6)	1652(1)
467(1)	1005(3)	3104(1)	467(0)	1002(0)	3118(1)
471(1)	1006(1)	3120(15)	480(1)	1010(4)	3133(16)
561(2)	1017(6)	3158(2)	562(3)	1021(4)	3164(5)
592(0)	1035(5)	3164(6)	582(2)	1037(3)	3169(7)
600(0)	1041(1)	3178(36)	594(0)	1044(1)	3184(35)
606(8)	1136(14)	3190(25)	606(8)	1138(13)	3192(0)
611(1)	1159(0)	3197(0)	611(1)	1160(0)	3197(21)
678(1)	1162(0)	3202(1)	682(1)	1161(0)	3198(3)
727(95)	1163(1)	3206(1)	727(76)	1166(1)	3198(2)
776(39)	1177(0)	3216(4)	763(24)	1179(0)	3211(6)
809(0)	1192(1)	3220(4)	812(0)	1196(1)	3218(6)
861(0)	1349(0)	3225(5)	846(0)	1351(0)	3222(6)

Table S54. Harmonic vibrational frequencies (in cm^{-1}) and infrared intensities (in parentheses, in km/mol) for the $C_5H_5IrC_7H_7$ structure **Ir3-57Q**

B3PW91			M06-L		
34(0)	831(2)	1361(0)	26(0)	817(5)	1368(0)
62(1)	833(3)	1373(10)	57(1)	821(10)	1378(12)
93(1)	841(1)	1387(0)	89(1)	829(0)	1387(0)
118(1)	854(2)	1390(5)	119(1)	854(1)	1402(4)
171(0)	879(0)	1441(6)	170(0)	875(0)	1442(4)
211(2)	899(1)	1449(3)	212(2)	889(1)	1453(1)
244(1)	902(0)	1452(1)	247(0)	890(4)	1455(1)
265(1)	903(4)	1456(3)	278(1)	908(0)	1460(1)
297(1)	914(5)	1491(0)	299(0)	919(4)	1496(0)
337(1)	932(0)	1513(23)	333(3)	931(0)	1520(19)
346(2)	949(0)	1586(40)	346(0)	943(0)	1586(40)
419(1)	955(5)	3149(1)	419(1)	954(5)	3154(2)
430(1)	991(2)	3154(2)	431(1)	984(3)	3156(3)
499(0)	1012(9)	3157(3)	502(0)	1014(7)	3159(3)
547(3)	1018(14)	3166(21)	539(5)	1021(12)	3171(17)
571(0)	1067(0)	3184(15)	569(1)	1070(0)	3187(17)
596(0)	1067(2)	3186(29)	597(0)	1070(2)	3187(33)
621(79)	1119(2)	3193(10)	619(61)	1131(2)	3195(10)
670(10)	1131(9)	3238(0)	662(8)	1133(14)	3232(0)
757(41)	1187(3)	3238(0)	723(33)	1190(3)	3233(1)
783(3)	1229(1)	3251(2)	779(3)	1234(1)	3246(3)
826(23)	1267(2)	3256(1)	811(0)	1270(2)	3254(2)
827(0)	1278(0)	3266(1)	813(4)	1272(0)	3264(1)

Table S55. Harmonic vibrational frequencies (in cm^{-1}) and infrared intensities (in parentheses, in km/mol) for the $C_6H_6NiC_6H_6$ structure **Ni1-66S**

B3PW91			M06-L		
-9(0)	863(2)	1361(0)	-16(0)	854(4)	1361(0)
64(0)	875(1)	1367(0)	62(0)	868(0)	1381(0)
93(0)	915(4)	1379(1)	93(0)	896(5)	1388(1)
134(0)	918(1)	1469(3)	136(0)	899(1)	1471(1)
154(0)	931(0)	1480(6)	160(0)	913(1)	1478(2)
177(1)	947(19)	1484(5)	196(1)	950(14)	1483(2)
205(0)	967(4)	1487(7)	231(0)	953(2)	1490(2)
241(2)	970(1)	1557(1)	248(1)	968(4)	1551(1)
332(37)	974(0)	1563(0)	336(27)	981(0)	1557(0)
384(3)	1003(1)	1572(14)	389(3)	1004(2)	1566(8)
399(0)	1010(0)	1634(2)	406(0)	1009(0)	1628(1)
432(12)	1011(3)	3144(1)	430(8)	1016(3)	3148(1)
456(0)	1030(4)	3157(9)	464(0)	1032(4)	3160(11)
592(0)	1032(6)	3165(2)	592(0)	1033(4)	3170(7)
610(3)	1038(6)	3173(13)	608(0)	1039(5)	3176(12)
612(3)	1041(2)	3182(0)	611(5)	1043(1)	3178(0)
615(0)	1152(4)	3183(33)	616(0)	1151(3)	3186(1)
640(0)	1163(0)	3189(1)	628(0)	1162(0)	3186(0)
698(0)	1165(0)	3191(0)	699(0)	1165(0)	3188(32)
708(83)	1168(0)	3195(17)	706(67)	1169(0)	3198(22)
745(30)	1177(1)	3203(16)	742(20)	1179(0)	3198(22)
830(0)	1190(0)	3203(16)	828(0)	1193(0)	3201(17)
837(70)	1360(0)	3212(9)	834(63)	1357(0)	3205(13)

Table S56. Harmonic vibrational frequencies (in cm^{-1}) and infrared intensities (in parentheses, in km/mol) for the $C_6H_6NiC_6H_6$ structure **Ni2-66T**

B3PW91			M06-L		
25(0)	809(74)	1352(26)	39(0)	790(4)	1347(0)
50(0)	834(4)	1356(13)	47(0)	798(0)	1373(28)
59(0)	845(1)	1363(2)	68(0)	842(0)	1381(0)
97(0)	864(124)	1425(3)	92(0)	845(5)	1417(0)
115(0)	925(6)	1438(2)	130(0)	880(0)	1446(2)
135(0)	932(0)	1473(6)	143(1)	909(5)	1447(0)
191(1)	946(223)	1483(17)	159(10)	933(0)	1465(0)
244(0)	959(5)	1489(37)	216(48)	933(0)	1484(56)
306(18)	966(17)	1543(185)	243(0)	943(359)	1489(0)
365(39)	977(19)	1580(300)	401(0)	971(0)	1573(271)
400(4)	980(5)	1608(9)	401(9)	977(29)	1614(0)
453(3)	1010(2)	3171(2)	457(2)	989(0)	3179(0)
457(7)	1014(1)	3172(1)	460(0)	1008(0)	3180(0)
572(0)	1019(14)	3177(15)	576(0)	1015(0)	3182(0)
589(11)	1039(5)	3179(2)	577(1)	1051(0)	3183(0)
607(1)	1046(2)	3180(6)	588(0)	1051(4)	3190(30)
609(1)	1110(1)	3186(19)	608(1)	1113(0)	3191(0)
627(2)	1147(31)	3196(14)	628(0)	1138(0)	3200(0)
682(21)	1151(18)	3196(26)	648(0)	1148(22)	3200(46)
695(48)	1166(20)	3198(7)	686(31)	1150(0)	3205(0)
709(7)	1170(2)	3200(18)	706(0)	1165(27)	3205(42)
772(8)	1185(35)	3208(4)	792(34)	1186(0)	3252(4)
800(106)	1345(2)	3208(2)	827(0)	1277(0)	3260(3)

Table S57. Harmonic vibrational frequencies (in cm^{-1}) and infrared intensities (in parentheses, in km/mol) for the $C_5H_5NiC_7H_7$ structure **Ni3-57S**

B3PW91			M06-L		
25(0)	840(1)	1283(0)	21(0)	827(9)	1282(0)
88(1)	846(9)	1342(0)	85(1)	836(4)	1343(0)
131(1)	851(6)	1379(23)	129(1)	838(9)	1381(16)
220(4)	871(5)	1387(0)	226(2)	863(5)	1392(11)
228(1)	905(0)	1391(13)	239(1)	887(0)	1393(0)
255(1)	912(1)	1431(0)	277(0)	891(1)	1410(6)
282(5)	920(15)	1439(5)	291(5)	915(21)	1429(0)
300(1)	948(1)	1454(7)	319(2)	942(2)	1455(4)
317(2)	949(15)	1485(1)	324(1)	947(12)	1486(0)
353(3)	981(2)	1650(1)	360(3)	975(2)	1652(3)
413(1)	989(2)	1661(1)	417(1)	980(3)	1663(3)
477(4)	993(0)	3098(24)	489(2)	998(1)	3102(26)
481(2)	1008(11)	3136(6)	496(1)	1010(8)	3136(4)
539(11)	1019(0)	3140(24)	520(8)	1022(0)	3141(17)
601(4)	1043(14)	3143(0)	597(9)	1045(10)	3141(1)
607(1)	1059(4)	3157(33)	602(1)	1049(7)	3154(43)
609(9)	1073(0)	3172(31)	605(3)	1075(1)	3172(35)
738(48)	1074(3)	3174(22)	738(45)	1076(0)	3174(30)
741(23)	1148(0)	3226(1)	743(16)	1149(0)	3225(2)
778(1)	1149(1)	3234(0)	765(0)	1149(3)	3230(0)
788(39)	1162(11)	3245(4)	783(28)	1161(16)	3242(6)
794(57)	1191(1)	3252(2)	792(34)	1186(0)	3252(4)
835(8)	1277(0)	3261(3)	827(0)	1277(0)	3260(3)

Table S58. Harmonic vibrational frequencies (in cm^{-1}) and infrared intensities (in parentheses, in km/mol) for the $C_6H_6PdC_6H_6$ structure **Pd1-66S**

B3PW91			M06-L		
9(0)	846(0)	1367(0)	13(0)	843(0)	1369(0)
39(0)	855(0)	1367(0)	31(0)	848(1)	1376(0)
40(0)	945(3)	1373(4)	38(0)	924(4)	1383(8)
84(0)	949(4)	1486(4)	58(0)	938(4)	1489(2)
97(0)	955(9)	1487(8)	91(0)	956(3)	1492(4)
104(0)	956(1)	1494(9)	110(0)	956(9)	1495(3)
115(0)	985(0)	1494(6)	116(0)	985(4)	1501(5)
262(0)	985(1)	1595(0)	231(19)	985(0)	1588(4)
269(63)	985(2)	1595(1)	259(28)	987(0)	1599(2)
424(9)	987(0)	1629(1)	397(0)	997(1)	1614(0)
427(0)	1017(1)	1629(2)	417(7)	1022(1)	1623(1)
437(0)	1017(0)	3162(0)	447(0)	1022(0)	3168(0)
439(0)	1043(5)	3163(0)	459(2)	1043(4)	3175(1)
602(0)	1043(3)	3174(2)	605(0)	1044(2)	3179(1)
606(0)	1046(3)	3175(0)	607(2)	1048(3)	3181(0)
610(0)	1047(3)	3175(0)	611(0)	1053(3)	3184(1)
610(6)	1162(0)	3176(0)	612(2)	1161(0)	3189(2)
699(145)	1162(0)	3187(11)	692(79)	1165(0)	3191(11)
702(2)	1181(0)	3187(10)	697(29)	1183(0)	3198(25)
703(2)	1182(0)	3195(18)	699(0)	1184(1)	3201(12)
704(0)	1191(0)	3195(21)	700(1)	1193(0)	3204(16)
838(35)	1191(0)	3203(15)	832(58)	1194(0)	3208(10)
844(14)	1365(0)	3203(5)	839(0)	1367(0)	3212(5)

Table S59. Harmonic vibrational frequencies (in cm^{-1}) and infrared intensities (in parentheses, in km/mol) for the $C_5H_5PdC_7H_7$ structure **Pd2-57S**

B3PW91			M06-L		
-17(0)	835(1)	1283(0)	-25(0)	824(0)	1280(0)
80(1)	839(4)	1342(0)	78(1)	836(11)	1344(0)
104(2)	847(6)	1377(25)	101(1)	837(4)	1381(20)
192(0)	870(7)	1379(0)	199(0)	863(6)	1386(1)
210(13)	898(0)	1388(17)	210(12)	882(0)	1392(15)
245(1)	905(1)	1417(4)	242(1)	889(0)	1406(5)
257(2)	920(19)	1425(0)	255(5)	913(22)	1427(0)
298(6)	945(1)	1452(8)	300(4)	943(2)	1457(6)
317(3)	949(13)	1483(1)	315(3)	947(10)	1486(0)
330(1)	979(1)	1653(4)	324(1)	975(1)	1655(7)
370(3)	980(2)	1667(1)	358(4)	980(4)	1668(2)
483(4)	998(2)	3105(20)	482(1)	989(0)	3108(22)
485(1)	1004(13)	3142(3)	484(3)	1008(10)	3143(3)
537(14)	1017(0)	3146(17)	516(12)	1020(0)	3147(15)
597(4)	1041(15)	3148(1)	595(8)	1044(11)	3149(1)
606(1)	1067(11)	3161(31)	605(1)	1052(13)	3161(34)
607(9)	1070(0)	3175(28)	605(3)	1074(0)	3178(29)
736(53)	1073(1)	3178(26)	734(44)	1076(3)	3180(30)
741(19)	1143(0)	3226(1)	741(14)	1146(1)	3227(1)
760(0)	1148(0)	3232(0)	744(3)	1151(0)	3230(0)
777(109)	1161(15)	3244(4)	770(85)	1161(19)	3243(7)
800(6)	1185(1)	3251(3)	789(2)	1185(1)	3253(4)
833(15)	1268(0)	3260(4)	815(12)	1276(0)	3261(4)

Table S60. Harmonic vibrational frequencies (in cm^{-1}) and infrared intensities (in parentheses, in km/mol) for the $C_5H_5PdC_7H_7$ structure **Pd3-57T**

B3PW91			M06-L		
16(0)	815(1)	1334(2)	23(0)	800(2)	1338(3)
47(0)	829(15)	1345(0)	57(0)	830(8)	1343(0)
71(0)	843(1)	1386(7)	66(0)	834(13)	1392(5)
104(1)	856(31)	1396(0)	107(1)	845(19)	1398(0)
153(0)	866(3)	1443(6)	144(0)	856(5)	1445(5)
187(0)	895(1)	1456(1)	174(0)	875(1)	1460(0)
190(5)	895(1)	1467(2)	187(6)	876(1)	1473(1)
209(1)	910(4)	1484(1)	199(2)	918(3)	1488(0)
258(5)	923(5)	1516(38)	222(3)	923(4)	1512(31)
356(0)	950(2)	1552(3)	359(0)	956(2)	1555(2)
382(1)	959(1)	1600(4)	377(0)	963(1)	1601(5)
425(7)	964(0)	3118(0)	423(4)	967(0)	3133(0)
428(1)	984(0)	3136(5)	430(1)	978(0)	3146(0)
545(0)	1004(11)	3141(8)	544(0)	1005(8)	3151(5)
572(6)	1035(12)	3144(7)	570(9)	1035(9)	3151(13)
584(0)	1059(2)	3170(9)	579(5)	1059(2)	3174(12)
588(7)	1068(2)	3173(33)	579(0)	1071(2)	3177(36)
658(90)	1142(0)	3182(20)	656(68)	1142(0)	3187(17)
756(1)	1170(4)	3232(0)	743(1)	1181(4)	3229(0)
766(1)	1202(10)	3237(0)	749(1)	1203(7)	3231(0)
772(97)	1202(3)	3247(2)	761(77)	1203(3)	3245(3)
790(3)	1263(2)	3254(1)	784(5)	1265(2)	3255(2)
813(3)	1285(0)	3264(3)	797(4)	1281(0)	3263(3)

Table S61. Harmonic vibrational frequencies (in cm^{-1}) and infrared intensities (in parentheses, in km/mol) for the $C_6H_6PtC_6H_6$ structure **Pt1-66S**

B3PW91			M06-L		
16(0)	852(62)	1364(0)	23(0)	853(72)	1366(0)
49(0)	880(0)	1377(0)	48(0)	871(5)	1379(0)
49(0)	944(4)	1386(3)	48(0)	929(7)	1391(8)
106(0)	952(8)	1469(6)	71(0)	936(4)	1470(3)
119(0)	954(0)	1472(7)	109(0)	950(10)	1484(4)
141(0)	955(4)	1487(7)	126(1)	951(4)	1490(3)
155(0)	970(0)	1487(8)	155(0)	969(1)	1497(6)
290(43)	970(0)	1578(0)	246(20)	980(0)	1569(7)
306(1)	983(0)	1578(3)	310(13)	987(0)	1591(2)
429(11)	986(0)	1640(2)	380(1)	992(1)	1610(0)
444(0)	1015(2)	1641(2)	436(14)	1018(2)	1636(1)
457(0)	1015(3)	3156(0)	475(0)	1020(1)	3157(0)
460(0)	1033(4)	3157(0)	494(0)	1034(4)	3168(3)
592(1)	1033(5)	3168(2)	596(0)	1036(2)	3175(1)
602(0)	1042(1)	3169(1)	602(4)	1044(0)	3177(1)
606(10)	1042(1)	3174(0)	607(0)	1053(3)	3185(3)
607(0)	1155(0)	3175(0)	609(4)	1155(3)	3185(5)
698(0)	1156(2)	3185(9)	691(1)	1163(0)	3191(2)
706(0)	1181(0)	3185(5)	703(0)	1182(0)	3195(27)
715(151)	1182(0)	3195(21)	710(101)	1183(1)	3203(10)
718(3)	1193(1)	3195(17)	718(21)	1194(0)	3205(16)
837(0)	1193(1)	3202(20)	837(0)	1195(0)	3206(14)
843(0)	1363(0)	3203(7)	842(0)	1361(0)	3214(7)

Table S62. Harmonic vibrational frequencies (in cm^{-1}) and infrared intensities (in parentheses, in km/mol) for the $C_5H_5PtC_7H_7$ structure **Pt2-57S**

B3PW91			M06-L		
20(0)	843(11)	1282(0)	23(0)	830(7)	1278(0)
88(1)	849(4)	1334(0)	86(1)	839(9)	1336(0)
111(2)	854(10)	1356(3)	108(2)	853(1)	1351(2)
180(6)	865(10)	1375(14)	180(12)	863(9)	1379(11)
226(11)	905(0)	1380(1)	221(7)	889(0)	1385(1)
264(0)	911(3)	1389(7)	257(0)	902(3)	1397(6)
276(0)	934(14)	1411(0)	271(0)	930(22)	1414(0)
309(5)	939(1)	1447(5)	306(5)	937(1)	1452(3)
331(2)	956(24)	1478(2)	329(2)	951(17)	1481(1)
369(1)	969(2)	1655(4)	359(1)	977(2)	1658(8)
392(1)	980(1)	1668(2)	390(1)	980(1)	1670(4)
523(6)	1009(9)	3094(24)	520(7)	1005(2)	3100(26)
534(3)	1009(2)	3138(2)	537(2)	1015(7)	3140(2)
566(4)	1027(0)	3150(2)	554(2)	1025(0)	3152(5)
595(1)	1035(14)	3153(1)	590(1)	1038(11)	3155(1)
603(11)	1072(0)	3158(45)	600(10)	1075(0)	3159(45)
613(0)	1073(2)	3180(28)	611(1)	1076(2)	3183(30)
735(54)	1100(2)	3182(34)	735(46)	1092(3)	3185(37)
747(16)	1140(1)	3234(1)	747(13)	1144(3)	3232(1)
774(4)	1142(0)	3237(0)	751(2)	1145(1)	3233(1)
792(106)	1144(10)	3248(3)	784(85)	1146(9)	3248(4)
825(1)	1182(7)	3258(1)	814(0)	1181(9)	3261(2)
837(1)	1257(0)	3266(3)	826(0)	1266(0)	3268(3)

Table S63. Harmonic vibrational frequencies (in cm^{-1}) and infrared intensities (in parentheses, in km/mol) for the $C_6H_6PtC_6H_6$ structure **Pt3-66T**

B3PW91			M06-L		
11(2)	843(4)	1348(1)	17(1)	829(1)	1349(0)
63(1)	844(0)	1348(0)	63(1)	831(3)	1359(0)
78(1)	866(0)	1361(44)	76(1)	859(0)	1374(36)
115(1)	891(0)	1405(0)	106(0)	863(5)	1400(1)
117(0)	893(12)	1437(11)	118(0)	879(6)	1439(4)
159(0)	921(71)	1439(0)	156(0)	923(23)	1442(2)
171(3)	931(3)	1449(4)	166(3)	929(45)	1453(5)
294(3)	932(0)	1488(27)	278(3)	933(0)	1489(21)
308(0)	936(15)	1490(0)	293(2)	940(1)	1494(6)
312(2)	946(0)	1548(47)	301(0)	948(0)	1554(27)
317(0)	968(18)	1577(0)	340(1)	968(18)	1583(1)
477(1)	973(0)	3176(0)	475(0)	979(4)	3179(1)
500(0)	1008(0)	3177(0)	497(0)	1011(0)	3181(1)
557(0)	1009(2)	3183(0)	556(0)	1013(1)	3184(0)
573(2)	1043(0)	3184(4)	571(2)	1042(1)	3185(0)
594(3)	1044(3)	3188(20)	595(2)	1046(2)	3191(16)
602(0)	1090(0)	3189(0)	600(0)	1093(0)	3192(1)
614(19)	1123(15)	3199(0)	618(17)	1126(20)	3201(16)
630(0)	1152(3)	3199(34)	634(0)	1152(0)	3201(26)
678(85)	1153(0)	3203(0)	666(37)	1153(1)	3205(14)
681(0)	1165(2)	3203(26)	678(31)	1168(1)	3206(19)
758(0)	1171(0)	3213(13)	743(3)	1175(0)	3214(12)
759(6)	1339(0)	3213(0)	764(3)	1347(0)	3214(2)
